# Personalised modelling of clinical heterogeneity between medium-chain acyl-CoA dehydrogenase patients

**DOI:** 10.1186/s12915-023-01652-9

**Published:** 2023-09-04

**Authors:** Christoff Odendaal, Emmalie A. Jager, Anne-Claire M. F. Martines, Marcel A. Vieira-Lara, Nicolette C. A. Huijkman, Ligia A. Kiyuna, Albert Gerding, Justina C. Wolters, Rebecca Heiner-Fokkema, Karen van Eunen, Terry G. J. Derks, Barbara M. Bakker

**Affiliations:** 1 Laboratory of Paediatrics, University of Groningen, University Medical Centre Groningen, Groningen, the Netherlands; 2 Section of Metabolic Diseases, Beatrix Children’s Hospital, University of Groningen, University Medical Centre Groningen, Groningen, the Netherlands; 3 Department of Laboratory Medicine, University of Groningen, University Medical Centre Groningen, Groningen, the Netherlands

**Keywords:** Medium-chain acyl-CoA dehydrogenase deficiency, Mitochondrial fatty acid oxidation, Personalised medicine, Metabolite partitioning, Kinetic modelling, Phenotypic heterogeneity, Inborn error of metabolism

## Abstract

**Background:**

Monogenetic inborn errors of metabolism cause a wide phenotypic heterogeneity that may even differ between family members carrying the same genetic variant. Computational modelling of metabolic networks may identify putative sources of this inter-patient heterogeneity. Here, we mainly focus on medium-chain acyl-CoA dehydrogenase deficiency (MCADD), the most common inborn error of the mitochondrial fatty acid oxidation (mFAO). It is an enigma why some MCADD patients—if untreated—are at risk to develop severe metabolic decompensations, whereas others remain asymptomatic throughout life. We hypothesised that an ability to maintain an increased free mitochondrial CoA (CoASH) and pathway flux might distinguish asymptomatic from symptomatic patients.

**Results:**

We built and experimentally validated, for the first time, a kinetic model of the human liver mFAO. Metabolites were partitioned according to their water solubility between the bulk aqueous matrix and the inner membrane. Enzymes are also either membrane-bound or in the matrix. This metabolite partitioning is a novel model attribute and improved predictions. MCADD substantially reduced pathway flux and CoASH, the latter due to the sequestration of CoA as medium-chain acyl-CoA esters. Analysis of urine from MCADD patients obtained during a metabolic decompensation showed an accumulation of medium- and short-chain acylcarnitines, just like the acyl-CoA pool in the MCADD model. The model suggested some rescues that increased flux and CoASH, notably increasing short-chain acyl-CoA dehydrogenase (SCAD) levels. Proteome analysis of MCADD patient-derived fibroblasts indeed revealed elevated levels of SCAD in a patient with a clinically asymptomatic state. This is a rescue for MCADD that has not been explored before. Personalised models based on these proteomics data confirmed an increased pathway flux and CoASH in the model of an asymptomatic patient compared to those of symptomatic MCADD patients.

**Conclusions:**

We present a detailed, validated kinetic model of mFAO in human liver, with solubility-dependent metabolite partitioning. Personalised modelling of individual patients provides a novel explanation for phenotypic heterogeneity among MCADD patients. Further development of personalised metabolic models is a promising direction to improve individualised risk assessment, management and monitoring for inborn errors of metabolism.

**Supplementary Information:**

The online version contains supplementary material available at 10.1186/s12915-023-01652-9.

## Background

Inborn errors of metabolism (IEMs) are distinct monogenetic diseases that cause pronounced systemic aberrations. While individually rare, the over 1450 different IEMs together have serious consequences in terms of morbidity and mortality, especially in children [1]. A better understanding of metabolism and its regulation, therefore, has wide implications [2–4]. Computational kinetic modelling of biochemical pathways is a promising tool for understanding and predicting complex pathway behaviour. Ultimately, coupling computational models of individual pathways could form a comprehensive representation of human biochemistry in silico*.* This concept has variously been referred to as the ‘Silicon Cell’ or the ‘Digital Twin’ [5–8].

IEMs provide a logical starting point for studying metabolism due to the presence of well-defined causative genetic variations. People with IEMs show large phenotypic heterogeneity, even between individuals carrying the same genetic variant [9]. Medium-chain acyl-CoA dehydrogenase deficiency (MCADD; OMIM #201450), the most common mitochondrial fatty acid oxidation (mFAO) disorder (mFAOD), is an illustrative example [10].

The deficient enzyme in MCADD, medium-chain acyl-CoA dehydrogenase (MCAD; EC: 1.3.8.7; UniProtKB: P11310) is one of a triad of enzymes in human cells (together with short- and very-long-chain acyl-CoA dehydrogenases, SCAD and VLCAD, respectively) that catalyse the first step of the mFAO. These three enzymes have distinct but overlapping substrate specificity [11]. Mainly before the age of 5 years, MCADD poses the risk of life-threatening metabolic decompensations elicited by fasting and/or infections [12, 13]. These metabolic decompensations are characterised by hypoketotic hypoglycaemia, metabolic acidosis and hepatic dysfunction [10]. Symptomatic patients might present with lethargy, nausea and vomiting and rapidly progress to coma or seizures. Avoidance of fasting and the implementation of an emergency regimen during illness can prevent the development of these symptoms [14–17]. In nine out of ten clinically ascertained MCADD patients [9, 18], homozygosity for the common c.985G > A *ACADM* variant (< 1% residual MCAD activity) is found. But this variant is *also* seen in many clinically asymptomatic patients [9]. The introduction of MCADD in newborn screening has substantially reduced morbidity and mortality [19, 20]. However, the newborn screening also identifies children with other *ACADM* variants, with higher residual MCAD activities, of which the clinical implications are incompletely understood. To improve individual patients’ risk assessment, management and monitoring, a better understanding of what underlies the phenotypic heterogeneity is needed [21, 22].

*C*oA *s*equestration, *to*xicity or *r*edistribution (CASTOR) has previously been posited as a possible pathogenic mechanism in MCADD and a number of other IEMs with a similar phenotype [23, 24]. A computational kinetic model of mFAO in mouse liver suggested that a loss of MCAD activity can indeed make the mFAO vulnerable to free CoA (CoASH) depletion, as well as to a decline of mFAO flux [25–27].

CoASH depletion and flux decline could impair ketogenesis and cause hypoglycaemia. Under fasted conditions, ketogenesis supplements the body’s energy needs by producing ketone bodies. To make ketone bodies, acetyl-CoA is used, of which around 85% comes from the mFAO [28]. The ketogenesis flux varies proportionally to the mFAO flux, increasing 1:1 when mFAO increases and vice versa [29–31]. Moreover, pharmacological inhibition of mFAO impairs gluconeogenesis [32]. This may be caused by the reduced availability of ATP for gluconeogenesis due to impaired mFAO flux [33, 34]. Alternatively, it may be due to a lack of gluconeogenic precursors, many of which are products or intermediates of the branched-chain amino acid degradation and tricarboxylic acid (TCA) cycle [34, 35]—both pathways that use CoASH as a substrate and would be impaired by CoASH depletion.

Here we developed a systems medicine approach to identify putative compensatory mechanisms in MCADD patients. We hypothesise that an ability to maintain sufficient mitochondrial CoASH and pathway flux might distinguish asymptomatic from symptomatic patients. Therefore, we constructed a computational, kinetic model of human liver mFAO—the first such model for human liver of which we are aware. Building on a previous model of mFAO in rat liver [26], we updated kinetic constants with human parameters, added two enzymes important to mitochondrial CoA metabolism. As an additional innovation, we made the spatial partitioning of hydrophobic acyl-CoA esters between the mitochondrial matrix and the membrane-bound enzymes explicit. The model was validated against experimental data, including the profile of even-chain saturated acylcarnitines in MCADD patient crisis urine samples. To personalise the model, targeted proteomics data were collected from fibroblasts of MCADD patients and used to adjust model parameters. The personalised model of an asymptomatic MCADD individual showed proteomic adaptations that increased CoASH and pathway flux, mainly increased SCAD.

## Results and discussion

### Construction of a human mFAO model

**Fig. 1:**
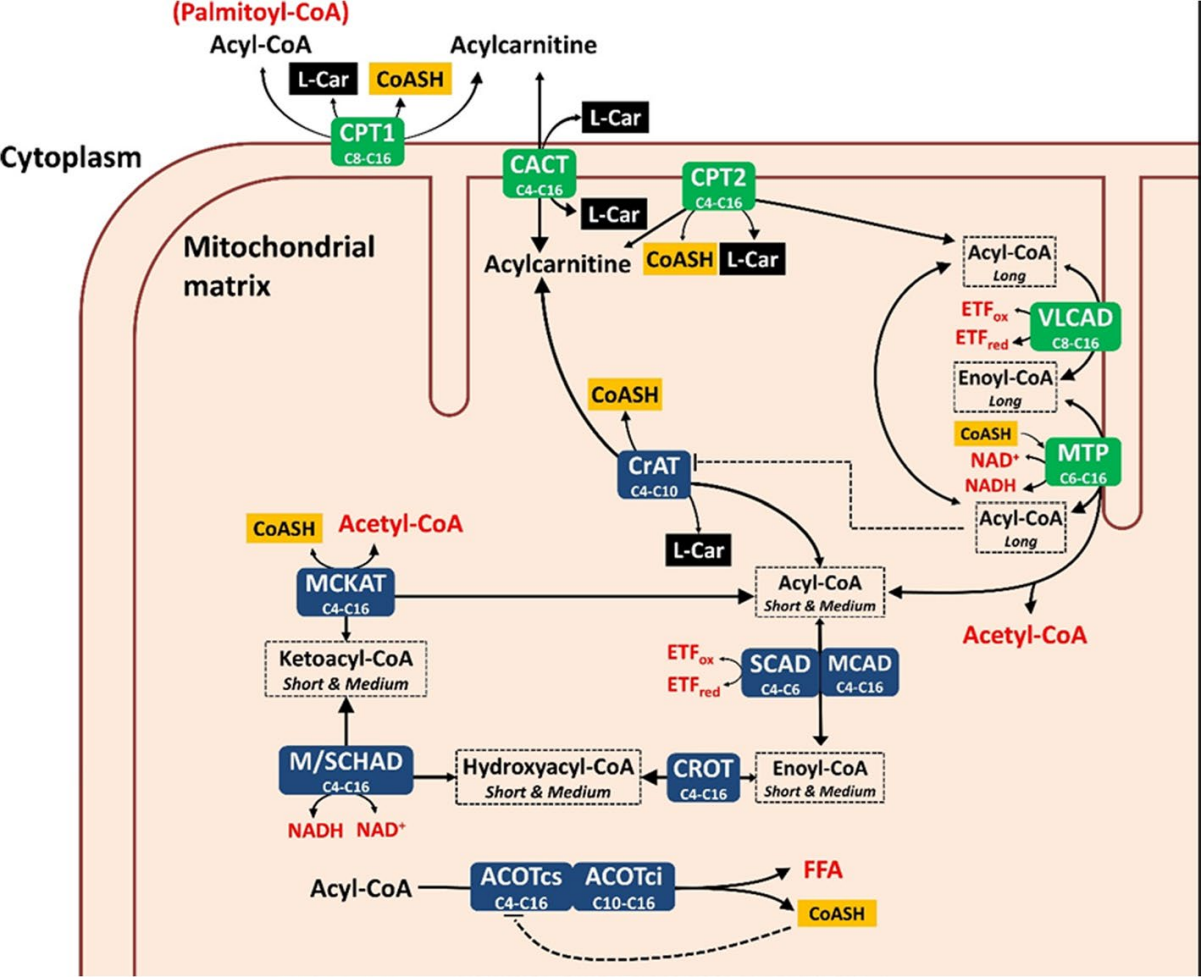
Schematic overview of the human hepatic mitochondrial fatty acid oxidation model. Enzyme names are written in white with their chain length specificity as subscript (C16, for example, for a 16-carbon acyl group). The enzymes depicted in green are membrane-bound enzymes while the blue boxes indicate soluble enzymes. These enzymes interconvert metabolites (in black text). Subscripts identify the metabolites’ chain lengths (*Long* or *Short & Medium*) and their primary localization according to their solubility. Metabolites in red are present at constant concentrations: ETF_ox_ and ETF_red_ refer to the oxidised and reduced form of the electron-transferring flavoprotein, and FFA indicates free fatty acids. Free coenzyme A (CoASH; yellow boxes) and L-carnitine (L-car; black boxes) are the non-acylated fraction of CoA and L-carnitine, of which the total pool forms a conserved moiety. The model includes the following enzymes: carnitine palmitoyltransferase 1a (CPT1; EC 2.3.1.21), carnitine/acylcarnitine translocase (CACT; PathwayCommons: O43772), carnitine palmitoyltransferase 2 (CPT2; EC 2.3.1.21), carnitine acetyltransferase (CrAT; EC 2.3.1.137), very-long-chain acyl-CoA dehydrogenase (VLCAD; EC 1.3.8.9), medium-chain acyl-CoA dehydrogenase (MCAD; EC 1.3.8.7), short-chain acyl-CoA dehydrogenase (SCAD; EC 1.3.8.1), crotonase (CROT; EC 4.2.1.17), medium- and short-chain hydroxyacyl-CoA dehydrogenase (M/SCHAD; EC 1.1.1.35), medium-chain ketoacyl-CoA thiolase (MCKAT; EC 2.3.1.16), mitochondrial trifunctional protein (MTP: EC 4.2.1.17, EC 1.1.1.211, EC 2.3.1.16), coenzyme A-insensitive acyl-CoA thioesterase (ACOTci; equivalent to ACOT2; EC 3.1.2.2), coenzyme A-sensitive acyl-CoA thioesterase (ACOTcs; equivalent to ACOT7 and ACOT13 combined; EC 3.1.2.2)

We constructed a dynamic model of human mitochondrial fatty acid β-oxidation, including the carnitine shuttle and mitochondrial acyl-CoA thioesterase activity (Fig. 1). A detailed description of the model is available in Additional File 1: Text S1 [11, 25–27, 36–152].

As a starting point, we took an existing kinetic model of mFAO in rat liver, which has been validated quantitatively with detailed acylcarnitine time-course data [26]. The kinetic parameters, where possible, were replaced by human liver-derived values. Metabolite pools were modelled as being located either in the cytosol or in the mitochondrial matrix, with distinct volumes. Long-chain acyl-CoA dehydrogenase (LCAD), which is not expressed in human liver [87, 98, 109], was omitted. The model was extended with three enzymes important to mitochondrial CoA metabolism: carnitine acetyl-CoA transferase (CrAT [120]), a CoASH-sensitive acyl-CoA thioesterase (ACOTcs, based on the kinetics of ACOT7 and ACOT13) and a CoASH-insensitive one (ACOTci, representing ACOT2) [131].

Where human liver kinetic parameters were not available, the most suitable alternatives were chosen. Values from other human tissues were privileged. If measurements from human tissues were not available, parameters from other mammals were also considered. If tissue-specific isoforms exist (e.g. carnitine palmitoyltransferase 1, or CPT1 [153]), parameters from the liver of other mammals were preferred over human parameters from other tissues. We used equilibrium constants (*K*_eq_) generated by the online tool eQuilibrator [66, 67]. Finally, all metabolites indicated in red in Fig. 1 were modelled as boundary metabolites with constant concentrations. This included acetyl-CoA, which was kept constant to allow the model to reach steady state. These constant boundary concentrations are model parameters, which can be varied in a realistic range, to assess their impact.

As a qualitative innovation, to reflect more accurately the physicochemical conditions inside the mitochondrion, we introduced partitioning of the mitochondrial metabolites between a water-soluble matrix pool and a hydrophobic mitochondrial inner-membrane pool. Longer acyl chains render metabolites less water-soluble, which causes a large fraction of the metabolite pool to localise to the membrane rather than dissolving in the aqueous matrix [55, 147, 152]. This would mean that membrane-embedded enzymes (green boxes in Fig. 1) would encounter a higher local concentration of these more hydrophobic intermediates, while soluble, matrix-localised enzymes (blue boxes in Fig. 1) would be exposed to a lower concentration of hydrophobic metabolites. Water-soluble metabolites, on the other hand, would diffuse more evenly throughout the mitochondrion, meaning that membrane-bound and soluble enzymes would be exposed to a similar, more dilute, concentration. It has been proposed that long-chain, hydrophobic acyl-CoAs ‘surface crawl’ along the mitochondrial inner membrane from active site to active site instead of diffusing into the bulk aqueous medium after each reaction [37, 38, 50, 133]. To mimic this phenomenon in the computational model, chain-length-specific ‘relative partitioning factors’ were introduced, which effectively split each mitochondrial metabolite pool into a fraction that reacts with the membrane-bound enzymes and one that reacts with matrix-localised enzymes. Enzymes were then also assigned a localisation—either membrane-bound or in the matrix. A more detailed discussion of the underlying assumptions and physical basis of metabolite partitioning is presented in the model description in Additional File 1: Text S1.

The final model contains the mitochondrial fatty acid oxidation reactions that pertain to even-chain, saturated acyl esters of 16 carbons or shorter [154]. All rate equations in the final model are reversible, except for those of ACOTci and ACOTcs, for which no reverse activity has been described [131]. Model simulations predict steady-state fluxes, metabolite concentrations, and dynamic rates. The model contains 49 variable metabolite concentrations, 75 reactions and 369 experimentally obtained parameters.

### Model validation

Steady-state fluxes and reaction rates were then simulated and compared to three sets of experimental and MCADD patient data. Details of unit conversions and simulation conditions are given in full in Additional File 2: Text S2 [28, 29, 69, 78, 84, 92, 97, 101, 155–163].

**Fig. 2:**
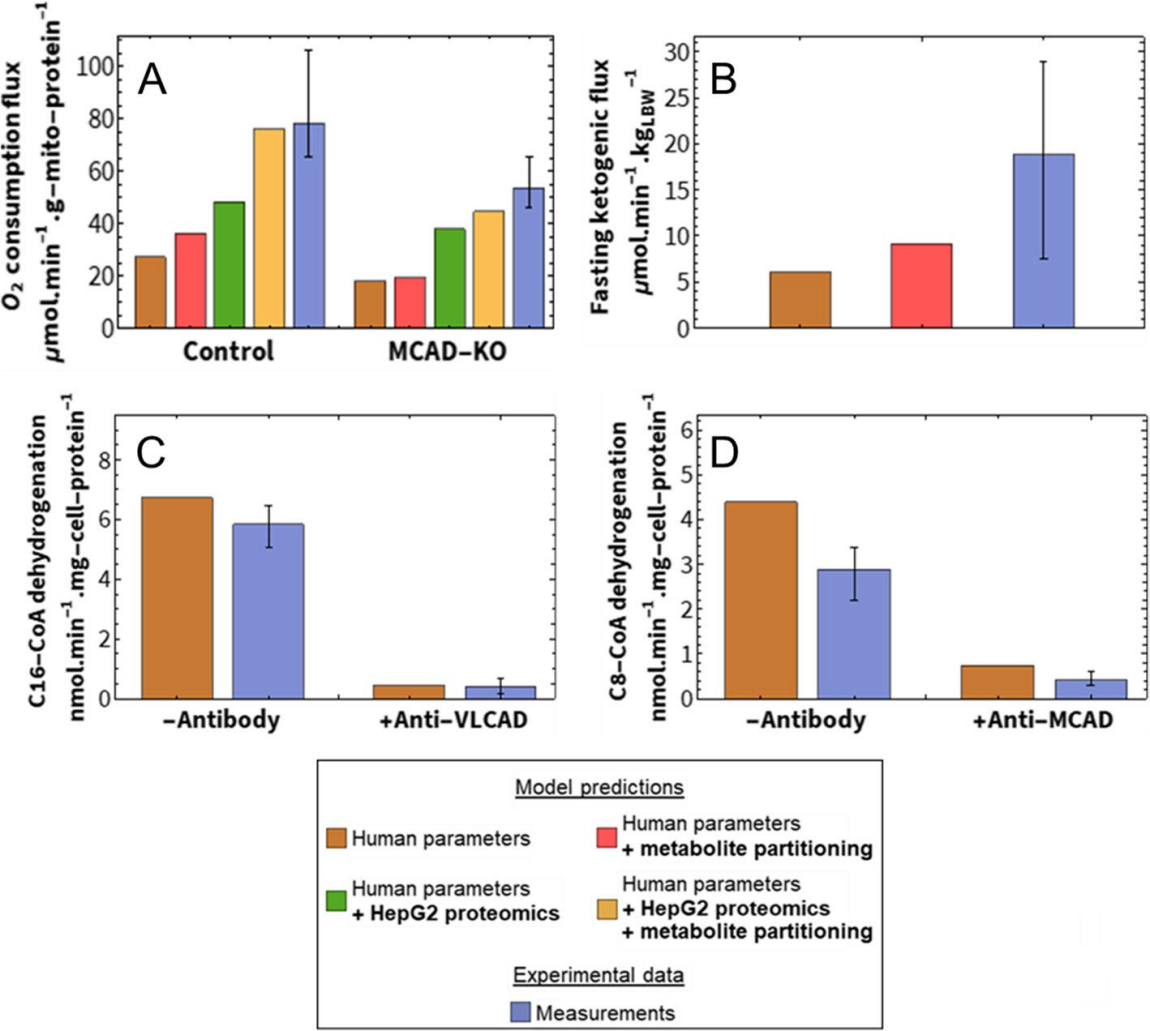
Model validation. Model predictions compared to experimentally measured data (blue). All model adjustments and conversions are described in Additional File 2: Text S2. The bars represent the mean of the different data, while error bars indicate the range of the data (minimum and maximum). **A** Simulated NADH production flux, stoichiometrically converted to O_2_ consumption, compared to the uncoupled oxygen consumption flux of permeabilised MCAD-KO and control HepG2 cells. In converting an NADH production flux to an oxygen consumption flux, the contribution of FADH_2_ and reducing equivalents from the downstream TCA cycle were also considered (Additional File 2: Text S2). The experiment and simulation contained 25 µM of palmitoyl-CoA and 2 mM of L-carnitine (blue; *n* = 4 in both groups). **B** Simulated acetyl-CoA production flux, stoichiometrically converted to a ketogenic flux, compared to measured ketogenesis in 24 h-fasted, healthy human subjects (*n* = 17). **C, D** Comparison of simulated and measured palmitoyl-CoA (C16-CoA, **C**) and octanoyl-CoA (C8-CoA, **D**) dehydrogenation rates (acyl-CoA substrate at 30 µM) in crude lysate compared to cell lysates after immunoprecipitation of VLCAD or MCAD, respectively (blue; *n* = 3 or *n* = 4)

First, oxygen consumption with palmitoyl-CoA as a substrate was measured in permeabilised control and MCAD-knockout (KO) HepG2 cells and compared to the fluxes predicted by the model (Fig. 2A). The knockouts were generated with CRISPR-Cas9 [164] and confirmed with genotyping (Additional File 3: Table S1), Western blot (Additional File 4: Fig. 1A) and proteomics (Additional File 4: Fig. 1B).

Since the reference model is based on human liver and these experiments were performed on HepG2s, the model was adapted based on literature-derived differences in protein concentration between healthy primary human hepatocytes and HepG2 cells [78, 159]. This was done by adjusting the *V*_max_ values in the model, which are linearly related to the corresponding enzyme concentrations. The effect of including metabolite partitioning and HepG2 proteomics in the model was assessed separately and in combination. The healthy control model predicted pathway flux within the range of the experimentally measured values after the inclusion of both metabolite partitioning factors and the adjustment of *V*_max_ values to HepG2 proteomics. Experimentally, the MCAD knockout reduced the mean O_2_ consumption by 30%, which was best reproduced by simulation when both metabolite partitioning and HepG2 proteomics were included in the model (Fig. 2A). The correspondence between the model and the experiment is encouraging, particularly in the light of the fact that proteomics data were from an independent study.

Second, we predicted whole-body ketogenic flux from the model simulations. Although the model does not directly predict ketogenic flux, the rate of acetyl-CoA production dictates the maximum rate of ketone body synthesis from fatty acids [29–31]. Fletcher et al. [28] measured whole-body ketogenic flux using stable isotope-labelled substrates in healthy adults after 24 h of fasting (*n* = 17). The ketogenic flux was predicted within the measured range by the human model, with the prediction from the model including metabolite partitioning more closely approximating the mean of the measurements (Fig. 2B). That the predictions are at the lower end of the measured values might be partially explained by ‘pseudoketogenesis’: a methodological issue that leads to artificially high ketogenic flux measurements [165, 166]. No suitable quantification of this phenomenon was available, to our knowledge, so we could not correct for it in the model itself; however, it does explain why our model predictions fall at the lower end of the measured range.

Third, in vitro octanoyl- and palmitoyl-CoA dehydrogenation rates were predicted. Aoyama et al. [97] measured these rates in crude human liver lysate (− antibody in Fig. 2D) and in lysate with immunologically inactivated VLCAD or MCAD (+ anti-VLCAD and + anti-MCAD, respectively). We mimicked the immunological inactivation of VLCAD and MCAD in the computational model by setting the maximum velocity (*V*_max_) of the inactivated enzyme to zero. Since the assay was performed in tissue lysate, in the absence of membranes, metabolite partitioning was not considered. The palmitoyl-CoA dehydrogenation rate and the effect of VLCAD inactivation were predicted within 5% of the measured range (Fig. 2C). The octanoyl-CoA dehydrogenation rate for both the crude and the MCAD-inactivated lysates was overpredicted by about 30%; however, both the model prediction and the measured data showed an 85% reduction of this rate upon MCAD inactivation (Fig. 2D). The discrepancy between the measured and predicted rates of octanoyl-CoA dehydrogenation might be explained by natural variation in gene expression, which can be quite large among mitochondrial enzymes [167]. Considering this natural variation, the agreement that we did find was already good.

Taken together, Fig. 2 shows that experimental data across different scales, ranging from lysates to cells to whole-body data, are reliably predicted by the model. A realistic representation of metabolite partitioning between the mitochondrial matrix and the hydrophobic space close to the membrane substantially improved the correspondence between the model and the experiments.

### Modelling a metabolic decompensation in MCADD patients

An important question was how accurately, and under which conditions, the model mimics the characteristics of MCADD patients experiencing metabolic decompensation. The model predicts acyl-CoA concentrations in the liver, but these are not accessible in patients. It is often assumed, however, that acylcarnitine patterns in blood or urine qualitatively reflect the acyl-CoA profile in the mitochondria [26, 45]. Therefore, acylcarnitine concentrations were quantified in urine from clinically asymptomatic and symptomatic patients, collected under *fed*, *fasted* or *metabolic decompensation* conditions. All patients were diagnosed before MCADD was included in the neonatal screening and symptomatic patients had not undergone any treatment or observation prior to the crisis, making this a precious and unique dataset. Asymptomatic patients were identified during proband follow-up after their siblings had been admitted to hospital with hypoketotic hypoglycaemia. Under fed and overnight fasted conditions, symptomatic and asymptomatic MCADD patients showed similar urinary acylcarnitine concentrations (Table 1 and Additional File 5: Table S2), with a characteristically increased C8 compared to other metabolites [168]. In healthy controls, C8 is at comparable levels to other acylcarnitines [169]. Acylcarnitine accumulation also seemed unaffected by an overnight fast. During a metabolic decompensation, the total concentration of acylcarnitines increased and the C8/C10 ratio reached its highest levels. The increased acylcarnitines mainly comprised free carnitine, acetyl-, hexanoyl- and octanoylcarnitine (Table 1).

**Table 1:** Patient urine acylcarnitine profiles

Parameter in µmol/mmol creatinine	Asymptomatic. *n* = 4 Median (min.–max.)		Symptomatic. *n* = 3 Median (min.–max.)		
	**Fed**	**Fasted**	**Fed**	**Fasted**	**Decompensation**
**Total acylcarnitine**	3.19 (2.93–37.49)	2.72 (2.04–4.84)	5.83 (2.16–30.92)	1.87 (1.56–9.94)	141.78 (28.03–198.87)
**C0**	0.95 (0.60–17.44)	0.47 (0.34–1.39)	2.36 (1.05–5.74)	0.63 (0.42–0.92)	27.15 (2.27–77.20)
**C2**	0.24 (0.18–8.77)	0.14 (0.09–0.94)	0.76 (0.11–18.68)	0.09 (0.02–5.08)	75.60 (8.11–98.28)
**C4**	0.22 (0.12–0.39)	0.14 (0.06–0.20)	0.14 (0.02–0.30)	0.03 (0.02–0.12)	1.17 (0.60–2.46)
**C6**	0.065 (0.04–0.74)	0.06 (0.04–0.09)	0.08 (0.01–0.50)	0.02 (0.01–0.23)	3.84 (0.58–4.06)
**C8**	0.295 (0.20–6.72)	0.26 (0.20–0.74)	0.67 (0.01–3.22)	0.16 (0.13–0.93)	8.34 (7.12–24.35)
**C10:1**	0.11 (0.07–0.55)	0.07 (0.05–0.11)	0.11 (0.03–0.52)	0.06 (0.03–0.15)	0.93 (0.38–1.57)
**C10**	0.04 (0.02–0.10)	0.04 (0.03–0.05)	0.05 (0.01–0.11)	0.02 (0.02–0.15)	0.53 (0.42–0.97)
**C12**	0.015 (0.01–0.04)	0.02 (0.01–0.02)	0.02 (0.01–0.03)	0.01 (0.01–0.03)	0.12 (0.07–0.25)
**C14**	n.d (n.d.–0.01)	n.d (n.d.)	n.d (n.d.–0.01)	n.d (n.d.–0.01)	0.15 (0.15–0.28)
**C16**	n.d (n.d.)	n.d (n.d.)	n.d (n.d.)	n.d (n.d.)	0.03 (0.01–0.03)
**C8/C2**	1.14 (0.77–1.29)	1.76 (0.79–2.89)	0.17 (0.09–0.88)	1.78 (0.18–6.50)	0.32 (0.08–0.88)
**C8/C10**	8.83 (7.20–67.20)	7.67 (6.25–14.80)	13.40 (1.00–29.27)	6.50 (6.20–8.00)	19.86 (13.43–25.10)

*n.d.* not detectable, i.e. < 0.01 µmol/mmol creatinine

**Fig. 3:**
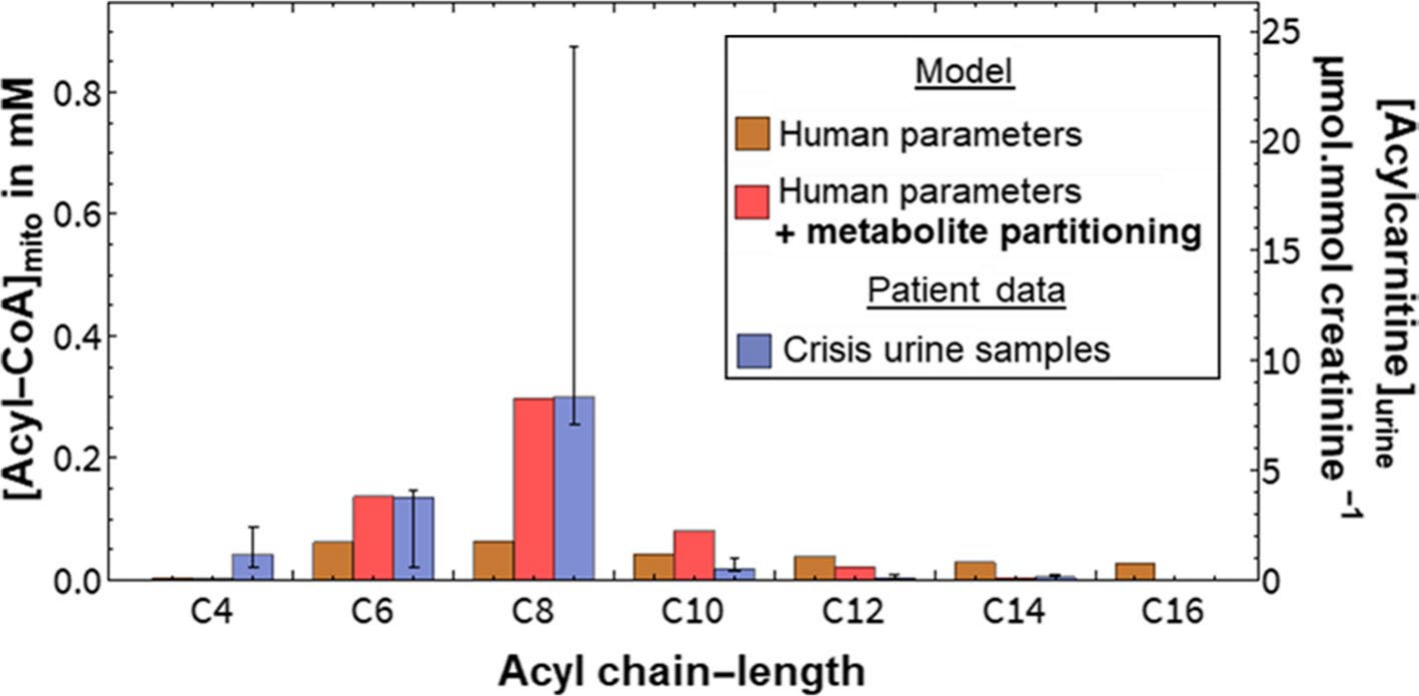
Patient crisis modelling. Simulated acyl-CoA accumulation in the mitochondrial compartment at 150 µM cytosolic palmitoyl-CoA compared to measured acylcarnitines in the urine of MCADD patients during hypoketotic hypoglycaemia (*n* = 3). The blue bars (measured data, *y*-axis on the right) represent the median and the range of the data (minimum and maximum)

We then compared the patterns of measured urinary acylcarnitines to liver mitochondrial acyl-CoAs predicted by model simulation under stress. To mimic stress, the cytosolic palmitoyl-CoA concentration was set to 150 µM: this is a realistic high-fat concentration in liver cells, such as under fasting conditions [150, 170]. A large influx of fat to the liver is characteristic of the stress conditions that might precipitate in metabolic decompensation in MCADD [10]. The chain-length distribution of mitochondrial acyl-CoAs at different cytosolic palmitoyl-CoA increments can be seen in Additional File 6: Fig. S2. Simulated patterns of mitochondrial acyl-CoA concentrations recapitulated those of hexanoyl- and octanoylcarnitines in patient crisis urine (Fig. 3). Interestingly, if the metabolite partitioning attribute was removed from the model, the accumulation of C8 and C6 was no longer apparent (Additional File 7: Fig. S3). This suggests that metabolite solubility is important to our understanding of pathogenicity in MCADD.

### MCADD could cause severe CoASH depletion

The isoenzymes SCAD and VLCAD catalyse the same reaction as MCAD, but with a preference for shorter or longer acyl-chain lengths, respectively. To get insight into the effects of these different acyl-CoA dehydrogenases (ACADs) on mFAO flux and CoA sequestration, we compared MCAD deficiency to SCAD and VLCAD deficiency.

**Fig. 4:**
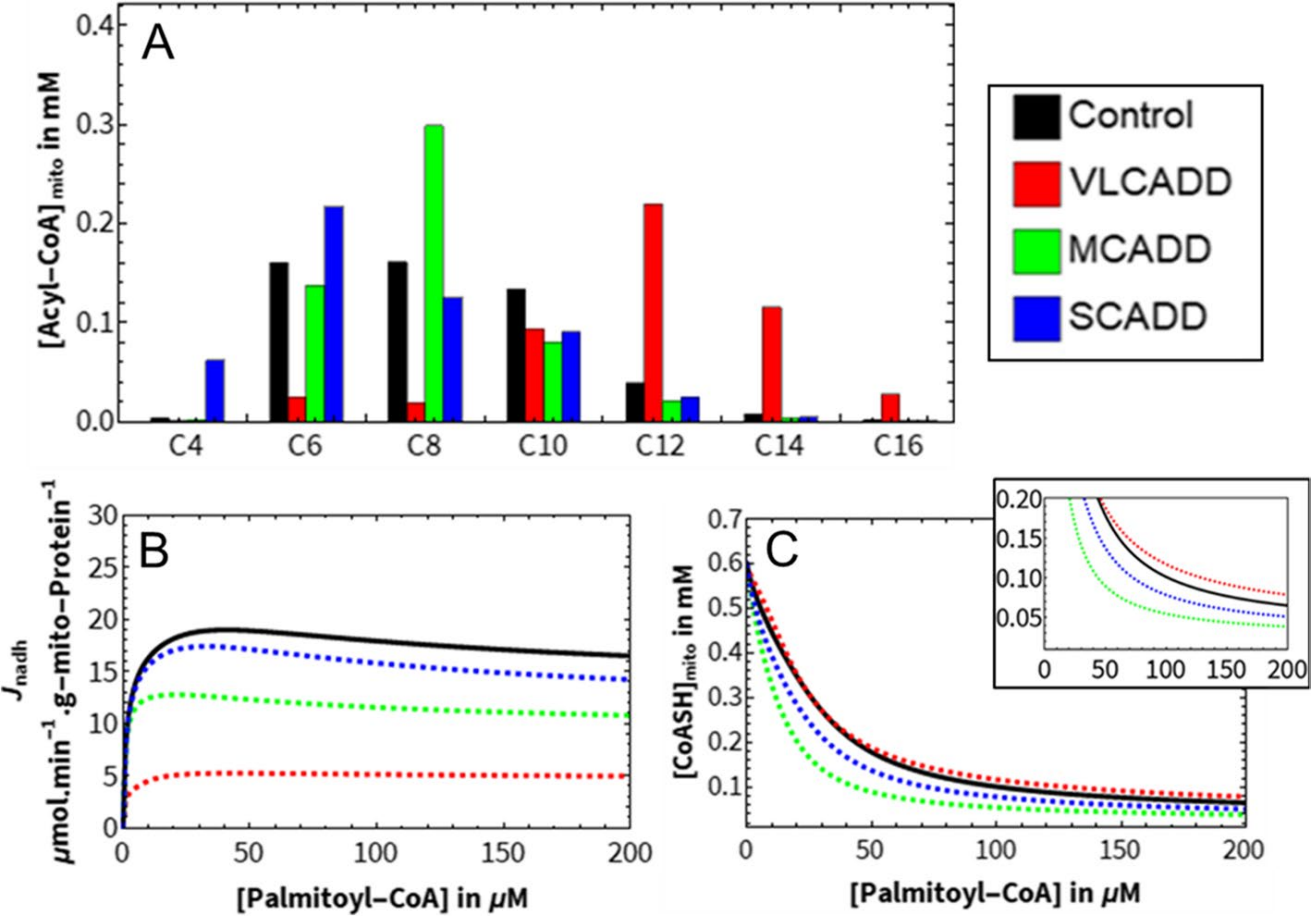
Comparison of different ACAD deficiencies in silico. The behaviour of one control and three different ACADD computational models of mFAO are shown. The residual activity reflects typical symptomatic patients: 0% for MCADD and SCADD, and 10% for VLCADD. All other ACADs have 100% of control activity. **A** Model mitochondrial acyl-CoA profile at 150 µM cytosolic palmitoyl-CoA. **B** NADH production flux at 150 µM cytosolic palmitoyl-CoA. **C** Steady-state mitochondrial CoASH. Inset gives the same data with a smaller *y*-axis

First, the acyl-CoA profiles of the three human acyl-CoA dehydrogenase deficiencies (ACADDs) were simulated at a high substrate concentration (Fig. 4A). Typical residual activities for symptomatic patients with the respective deficiencies were chosen: 0% activity for MCADD and SCADD [171, 172], and 10% for VLCADD [173]. The acyl-CoA species that accumulated in each disease, matched the substrate preferences of the deficient enzymes: C12, C14 and C16 in VLCADD, C8 in MCADD and C6 and C4 in SCADD. These chain lengths are all known to accumulate as blood acylcarnitines in patients with the corresponding ACAD deficiency [19, 174–178]. mFAO flux was quantified as the rate of production of one of its end products, NADH (Fig. 4B). The SCADD model maintained a residual flux of more than 90% of the control. In contrast, the MCADD model reached a maximum flux of about 60%, and the VLCADD model no more than 30% of control. This reflects the relative severity of the corresponding deficiencies [179–181]. It also highlights one of the strengths of the model, namely that the ACAD isoenzymes with overlapping substrate specificity in part compensate for each other. Consequently, a full ablation of SCAD or MCAD did not entirely block the flux through the pathway.

Figure 4C shows the free, non-esterified mitochondrial CoASH concentration as a function of the concentration of the cytosolic substrate palmitoyl-CoA. All models started with the same amount of CoASH, which decreased sharply as a function of an increasing palmitoyl-CoA concentration. MCADD caused the sharpest decline of CoASH and led to the lowest free CoASH concentration, about 50% of the control model at high palmitoyl-CoA. In the VLCADD model, CoASH remained even higher than in the control model. In VLCADD, the sequestration of CoASH is limited by the fact that the blockage occurs at the beginning of the pathway. First, VLCAD is upstream of MCAD and SCAD. Second, the long-chain acyl-CoA esters that are still formed (Additional File 6: Fig. S2B) cause a strong product inhibition (Additional File 1: Text S1) of the carnitine shuttle, thus limiting the further entry of substrate into the pathway. These effects also contribute to the low flux in VLCADD.

The combined results point to a qualitative difference between SCADD, MCADD and VLCADD. SCADD and MCADD lead to the accumulation of short- and medium-chain acyl-CoAs, which only weakly inhibit the carnitine shuttle. This permits substrates to continue entering the mitochondrion even at very high acyl-CoA concentrations and thereby allows more extensive sequestration of the CoA pool. One might interpret these results as indicating that MCADD and SCADD are acute CASTOR diseases, MCADD the more severe, in which an increase in substrate concentration can lead to a depletion of CoASH [23]. In MCADD, we see a combination of two effects: a substantial reduction in pathway flux and severe CoASH depletion. In this model, the flux was decreased even if CoASH was set to a constant concentration (Additional File 8: Fig. S4), indicating that the reduced flux is not a consequence of CoASH depletion but simultaneous with it. A model version without metabolite partitioning exhibited no CoASH depletion, milder acyl-CoA accumulations and smaller differences in pathway flux (Additional File 7: Fig. S3).

MCADD poses a threat to mitochondrial metabolism because it simultaneously causes the loss of about 35% of pathway flux relative to the control, as well as a 40% drop in CoASH. This is in contrast to VLCADD, which substantially decreases flux by 70%, but not CoASH. SCADD lowers both, but less strongly than MCADD (10% flux reduction and 20% CoASH reduction). In our subsequent analyses, we further investigated both of these effects.

### Metabolic control analysis identifies possible rescues for MCADD

**Fig. 5:**
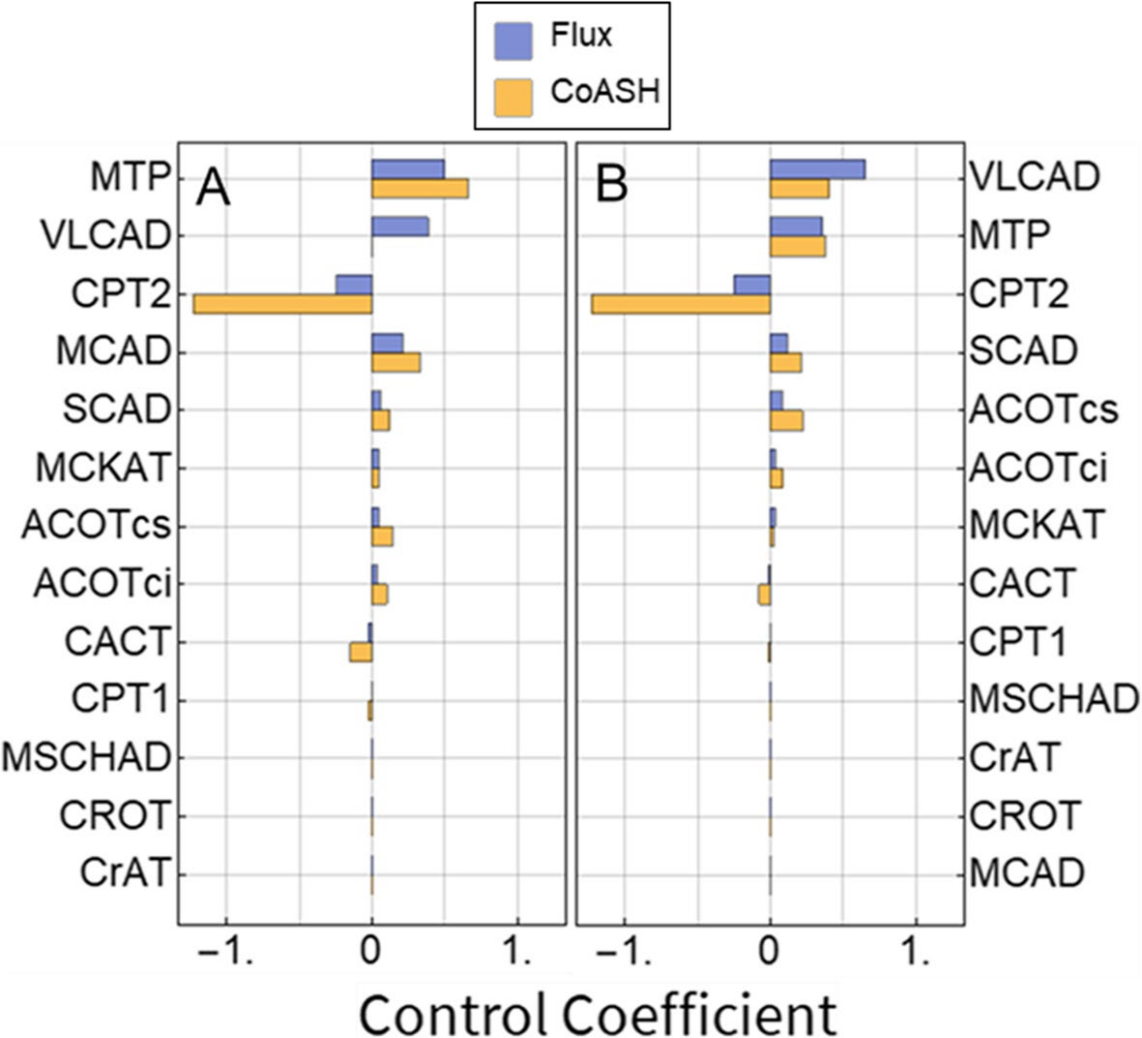
Metabolic control analysis. Flux and mitochondrial CoASH concentration control coefficients (blue and yellow, respectively) in a control (**A**) and an MCADD (**B**) model. Enzymes are displayed in descending order according to absolute flux control. Flux was defined as the sum of all NADH-producing reactions. Simulations were carried out at 150 µM cytosolic palmitoyl-CoA

We hypothesised that clinically asymptomatic MCADD patients may implement compensatory mechanisms that increase pathway flux and CoASH concentration. To identify these, we analysed which enzymes exert a large control over these two model readouts (Fig. 5).

Metabolic control analysis is a theoretical framework that quantifies the sensitivity of the steady-state outputs of a metabolic network to changes in the underlying parameters [139]. Flux control coefficients (Fig. 5A) quantify the percentage change in the flux in response to a 1% increase in enzyme concentration. The flux, again, refers to the rate of NADH production. A positive flux control coefficient means that the flux increases in response to an increased concentration of a given enzyme, while a negative value indicates that flux goes down as the concentration of that enzyme goes up [139]. MTP, VLCAD and MCAD had the largest positive flux control coefficients in the healthy control model, while CPT2 had a large negative flux control coefficient, and the third-highest absolute value (Fig. 5A). In the MCADD model, this pattern was the same, except that no flux control coefficient exists for MCAD, leaving SCAD in fourth place and ACOTcs not far behind (Fig. 5A).

Interestingly, CPT1, which is canonically considered the dominant flux-controlling enzyme of the mitochondrial β-oxidation [11], has a very low flux control coefficient in our model. This is in line with other studies, which show that depending on the physiological conditions, control may shift from CPT1 to downstream enzymes [27, 182, 183]. As expected, under conditions of high malonyl-CoA (100 μM), low cytosolic palmitoyl-CoA (2 μM) and abundant free mitochondrial CoA (no sequestration outside mFAO assumed), mitochondrial acetyl-CoA at 120 μM as in fed rats the CPT1 has a much higher FCC (Additional File 9: Fig. S5).

Concentration control coefficients (Fig. 5B) quantify the percentage change in specific steady-state metabolite concentration in response to a 1% increase in enzyme concentration. CPT2 had a large negative concentration control coefficient with respect to the mitochondrial CoASH concentration (Fig. 5B), in both the healthy control and MCADD models. Other enzymes with large flux control coefficients all had large positive concentration control coefficients with respect to the CoASH concentration, except for VLCAD, which exerted no control at all on CoASH in the healthy control. At a low mitochondrial acetyl-CoA concentration (120 µM; [184]), the concentration control coefficient of VLCAD towards CoASH even became negative (Additional File 10: Fig. S6). Response coefficients (which are calculated in the same way as control coefficients but pertain to all parameters) were also computed for the control model (Additional File 11: Table S3). VLCAD, MTP, CPT2 and MCAD parameters often showed strong responses, as did parameters that directly increase or sequester free CoASH (e.g. total CoA and acetyl-CoA concentration). This sensitivity analysis is consistent with the flux and concentration control coefficients.

Taken together, these results indicate a sensitivity of the pathway flux and CoASH to changes in the levels of CPT2, VLCAD, SCAD, MTP and the ACOTs. For most of these enzymes, increased levels would result in increased CoASH and flux. For CPT2, the relationship is inverted. Most interestingly, VLCAD, which always has a positive flux control, can have either positive or negative control over the CoASH concentration, depending on the conditions at which the analysis is performed. These suggested rescue mechanisms could now serve as candidates for explaining some of the phenotypic heterogeneity between MCADD patients.

### Increased SCAD, MTP and ACOT could attenuate CoASH depletion and flux decline

**Fig. 6:**
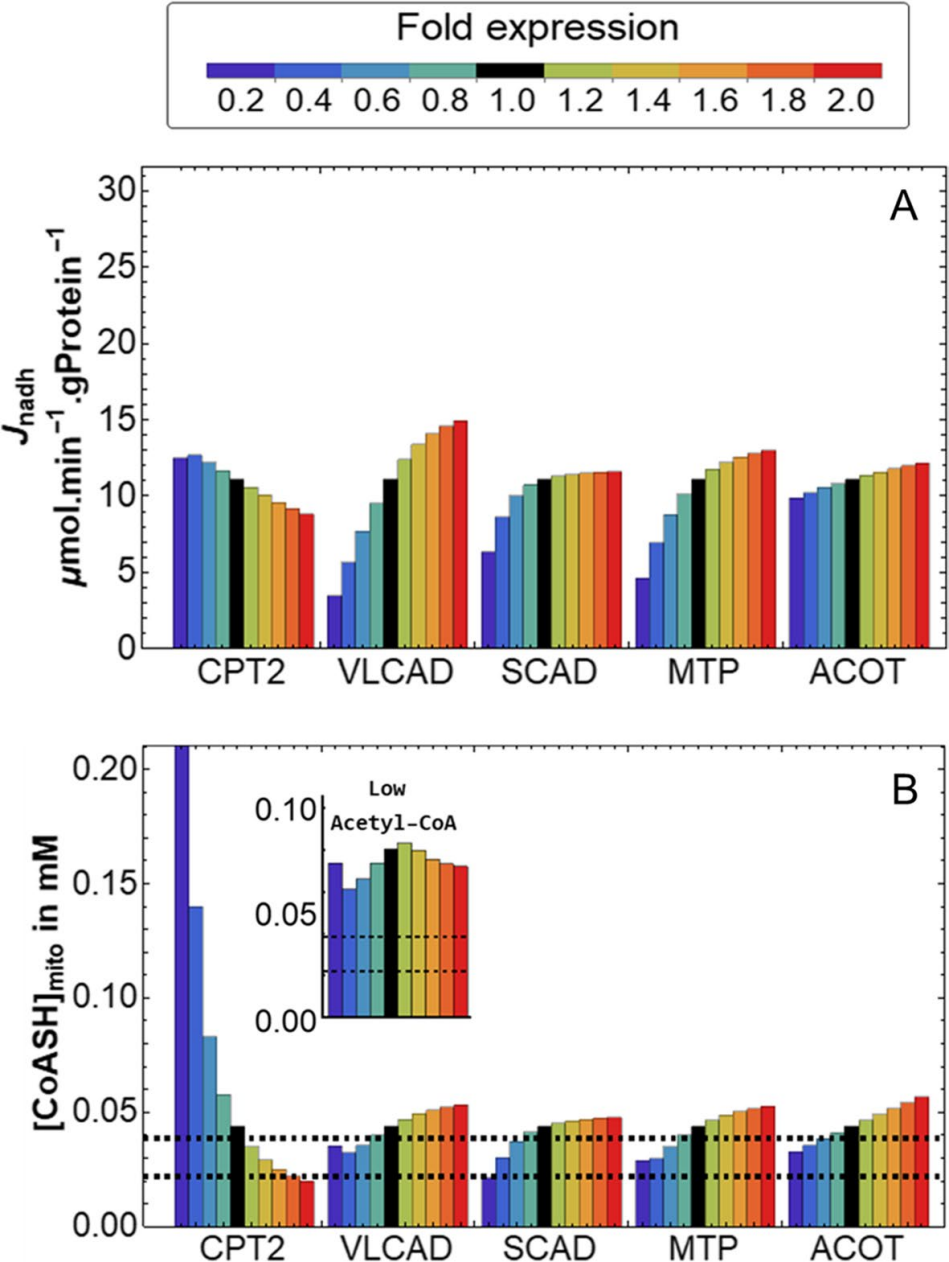
Possible rescues of CoASH and steady-state mFAO flux in an MCADD model. An MCADD computational mFAO model was simulated at different expression levels of the enzymes identified as possible rescues by metabolic control analysis. The reference value (onefold expression) is given in black. All simulations were performed at 150 µM cytosolic palmitoyl-CoA. The dashed lines in **B** indicate the *K*_m_ values of various mitochondrial enzymes that require CoASH as substrate (Table 2). CPT2, VLCAD, SCAD, MTP and ACOT were varied between 20 and 200% of basal expression levels. ACOT was varied by simultaneously increasing the expression of both ACOTs in the model. The inset shows the trajectory of CoASH concentration when VLCAD is incrementally varied with the mitochondrial acetyl-CoA concentration set to 120 µM (lowest value retrieved from literature), compared to the default concentration of 700 µM. **A** NADH production flux. **B** Steady-state mitochondrial CoASH

A limitation of metabolic control analysis is that it only considers small changes in enzyme activities, while larger changes are required for a substantial rescue. Therefore, six enzymes with high absolute flux or CoASH concentration control in the MCADD model (VLCAD, CPT2, MTP, SCAD, ACOTcs and ACOTci) were varied incrementally from 20 to 200% of their default levels (Fig. 6). The two ACOTs were varied together for simplicity. In agreement with its negative flux control (Fig. 5), decreasing CPT2 increased the pathway flux, while the other enzyme concentrations needed to be increased to get this effect (Fig. 6A). At the higher SCAD or MTP levels, the flux increase plateaued, as other enzymes took over control. In agreement with its negative CoASH concentration control, decreasing CPT2 increased CoASH, while increasing SCAD, MTP and ACOT, which had positive CoASH concentration control, indeed did increase mitochondrial CoASH (Fig. 6B). In the MCADD model, CoASH (Fig. 6B) was always lower than in the control model (Additional File 12: Fig. S7).

To see what would be the impact of increasing CoASH in the MCADD model, we compared it to the *K*_m_ values of mitochondrial enzymes that consume CoASH. These included enzymes of the mFAO but also from the TCA cycle (0.0022–0.0888 mM; Table 2 and dashed lines in Fig. 6B). A *K*_m_ is an indication of the metabolite concentrations at which an enzyme is most sensitive to concentration changes, so it serves as a guide to where CoASH might become limiting. In the MCADD model, the CoASH concentration varied in the range of the *K*_m_ values, implying that even a small increase should have a positive impact on the activity of CoASH-dependent pathways. In comparison, in the control model CoASH concentrations were much higher than the *K*_m_ values at any condition (Additional File 12: Fig. S7), implying that changes in the CoASH concentration would have little impact on the activity of these CoASH-consuming enzymes.

**Table 2:** *K*_m_ values for CoASH of CoASH-requiring mitochondrial enzymes

Enzyme	Pathway	*K*_m_ (mM)	Reference
CrAT	Carnitine shuttle	0.038–0.0888	[86, 91]
MCKAT	mFAO	0.0022–0.0384	[108, 185]
αKDH	TCA cycle	0.0025	[186]
PDH	TCA cycle / glycolysis	0.013–0.025	[186, 187]

In the MCADD model, increasing VLCAD leads to an increase of CoASH (Fig. 6B). However, if the concentration of acetyl-CoA is low (120 µM [184]), increases in VLCAD can cause a reduction in CoASH (inset in Fig. 6B). This may be realistic because a reduced mFAO flux might reduce the acetyl-CoA concentration [29–31]. This suggests a scenario in which increased VLCAD could aggravate CoASH depletion (Table 2).

It may seem surprising that increased ACOT led to consistent flux increases, in both an MCAD (Fig. 6B) and a control (Additional File 12: Fig. S7B) model. Given that ACOTs siphon off pathway intermediates from the β-oxidation, one would expect lower, not higher, NADH production. This suggests that at our chosen simulation conditions CoASH may be limiting or CoA esters might be inhibitory. If CoASH is set to constant levels, increased ACOT decreases flux in the control model but increases it in the MCADD model (Additional File 13: Fig. S8). In the control model, the negative effect of losing pathway intermediates via ACOT seems to dominate. In the MCADD model, however, ACOTs still have a positive effect on pathway flux, although more modest, even if ACOTs do not affect CoASH anymore. We conclude that not only depleted CoASH but also accumulating CoA esters inhibit pathway flux in MCADD. ACOTs alleviate both of these. This is in agreement with the idea that ACOTs modulate acyl-CoA levels and free up CoASH during periods of high substrate influx into the mitochondrion [131, 188].

In summary, the upregulation of SCAD, MTP and the ACOTs, as well as the downregulation of CPT2, were found to have a positive effect on flux and CoASH concentration in MCADD under overload conditions. This renders them potential compensatory mechanisms for mFAO function. A patient with one of these adaptations would be less at risk of a disastrous CoASH depletion than one without them. This is potentially useful information for risk stratification.

### Increased SCAD and MTP in a clinically asymptomatic MCADD patient lead to higher flux and CoASH

Proteome remodelling is known to occur in response to enzyme deficiencies [167, 189]. As shown above, certain changes in protein levels could theoretically lead to an increased flux and CoASH. To test whether the corresponding adaptations were recapitulated in real patients, targeted proteomics were measured on fibroblasts from 5 control and 10 MCADD patients [109].

The MCADD patients belonged to different phenotypic groups. The members of the ‘control’ group are not suspected to have a metabolic disease (*n* = 5). The MCADD patients were all diagnosed before neonatal screening for MCADD was implemented. They were subdivided into three phenotypic subgroups: ‘symptomatic’ (*n* = 4), i.e. patients that were clinically diagnosed upon admission to hospital with hypoglycaemia; ‘early diagnosis’ (‘ED’, *n* = 5), patients that were diagnosed at a young age (0–11 years) as siblings of the symptomatic patients and then put on preventative dietary regimens; and one ‘asymptomatic’ patient, who was discovered as an adult (30 years) and never exhibited clinical symptoms nor received treatment (*n* = 1).

**Fig. 7:**
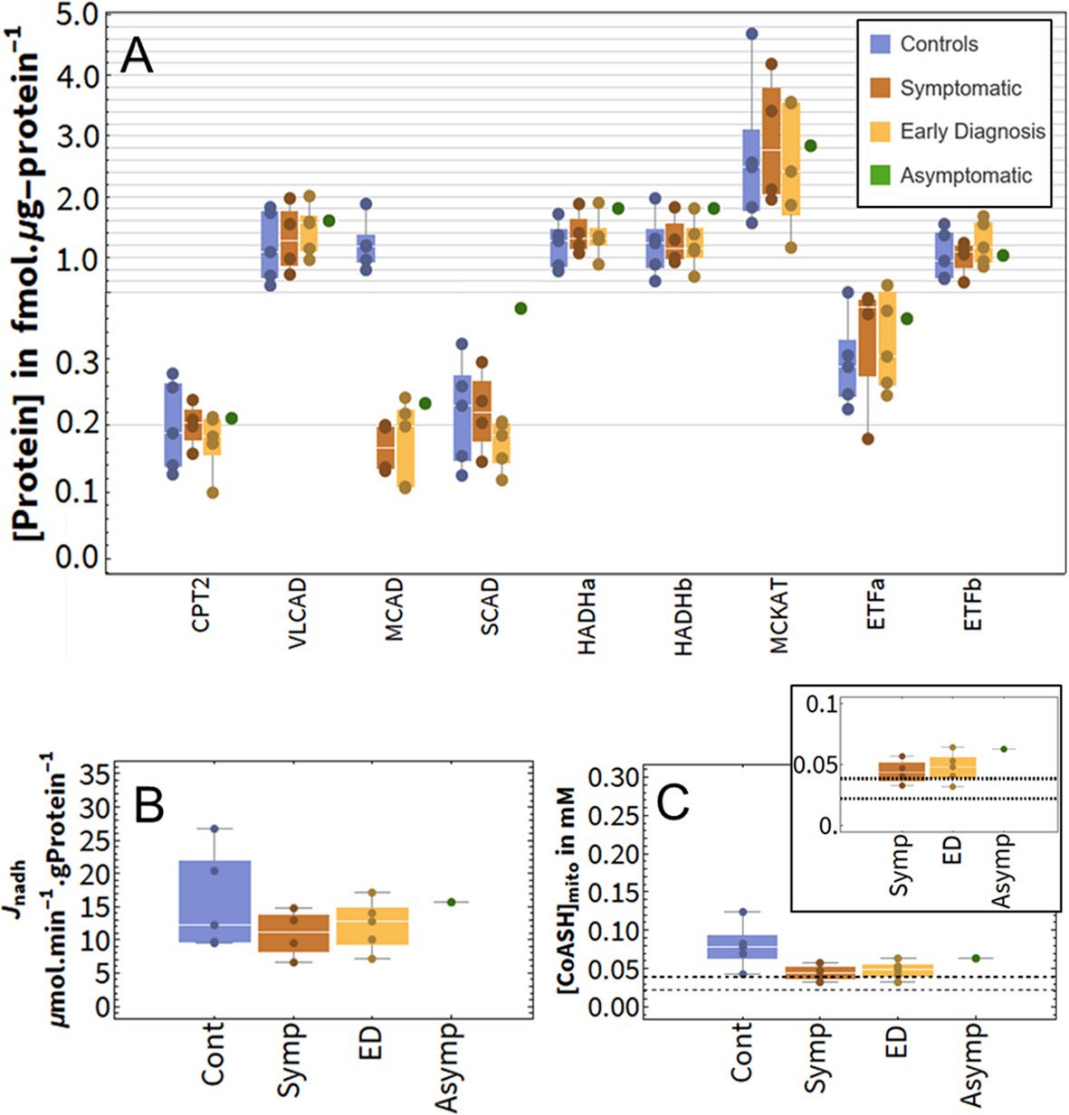
Steady-state mFAO flux and mitochondrial CoASH in personalised patient models. Fibroblasts proteomics from four phenotypic groups were used to personalise the kinetic model of mFAO: *Controls* (*n* = 5), *symptomatic* (*n* = 4), *early diagnosis*, (*ED*, *n* = 5) and *asymptomatic* (*n* = 1). All simulations were done at 150 µM cytosolic palmitoyl-CoA. **A** Normalised, measured expression of key β-oxidation proteins per clinical group. Individual people are averages of at least three technical replicates and are represented as data points in the distribution bars. HADHa and HADHb are the two subunits of MTP. **B, C** Simulation results from personalised computational models: flux and mitochondrial CoASH concentration. Dashed lines indicate the region of the *K*_m,CoASH_ values of key mitochondrial enzymes: PDH, αKDH, MCKAT and CrAT (Table 2). Control and MCADD were compared using a *t*-test

As expected, cells from MCADD patients had undetectable MCAD protein levels, in contrast to those from the control group (Additional File 14: Fig. S9, original data available at [190]). The other protein concentrations of symptomatic and ED patients did not differ significantly from each other, nor from the control group. Table 3 shows the average protein concentrations of the asymptomatic patient, of all groups together, and of the MCADD groups together (all excluding controls). The asymptomatic patient had the highest average SCAD level out of any of the subjects (Fig. 7A). Moreover, the asymptomatic patient’s SCAD was above the upper limit of the 95% confidence interval when all subjects were combined into one group (Table 3). This was not the case for any other peptide. The two subunits of MTP (HADHa and HADHb) were at the higher end of the concentration range in the asymptomatic patient (Fig. 7A), but still within the confidence interval. CPT2’s concentration did not differ between the asymptomatic patient and the other subjects in the cohort. ACOT was not included in the applied proteomics panel.

**Table 3:** Peptide concentrations per group (fmol.μg-total-protein^−1^)

Peptide	Asymptomatic	All subjects (*n* = 14)	All MCADD (*n* = 10)
	**Mean** **(95% confidence intervals, based on t distribution)**		
**CPT2**	0.21	0.19 (0.09–0.29)	0.19 (0.10–0.28)
**VLCAD**	1.60	1.33 (0.30–2.36)	1.41 (0.44–2.38)
**MCAD**	0.23	0.52 (-0.65–1.68)	0.18 (0.06–0.29)
**SCAD**	0.38	0.21 (0.06–0.37)	0.21 (0.04–0.38)
**ETFa**	0.36	0.32 (0.14–0.50)	0.34 (0.13–0.54)
**ETFb**	1.03	1.09 (0.37–1.81)	1.12 (0.37–1.81)
**MCKAT**	2.84	2.68 (0.53–4.83)	2.71 (0.58–4.83)
**HADHa**	1.80	1.34 (0.57–2.10)	1.41 (0.64–2.17)
**HADHb**	1.80	1.26 (0.35–2.18)	1.29 (0.39–2.20)

The computational mFAO model was then personalised using the proteomics data and fluxes and CoASH concentrations simulated per person. To this end, the *V*_max_ of each of the enzymes was multiplied by the corresponding enzyme concentration in an individual relative to that of the average control. This yielded a model of the liver mFAO in each individual patient. First and foremost, the asymptomatic patient model had a higher pathway flux than all the symptomatic MCADD patient models. None of the other groups differed significantly from each other in terms of flux, including the control group. The mitochondrial CoASH of all MCADD models differed from that of the control models (*p* = 0.080 with the asymptomatic patient model included; *p* = 0.061 when excluded), suggesting a trend that might be validated with a larger sample size. In addition, the asymptomatic patient model showed higher CoASH than all symptomatic MCADD models (Fig. 7B, C), even reaching the range seen in the healthy control models. Since the CoASH concentrations remain close to the *K*_m_ values of CoASH-dependent enzymes in the mitochondrion (Table 2), this elevated CoASH concentration in the asymptomatic patient is likely to have a stimulatory effect on CoASH-dependent reactions.

If we hypothesise that flux decline and CoASH depletion contribute to the development of hypoketotic hypoglycaemia, then our results suggest that altered expression of genes encoding for SCAD and MTP has contributed to the lack of symptoms in the asymptomatic patient. The ED patients did not show such adaptations. Of course, the model is blind to adaptations outside its included reactions, such as in gluconeogenesis or ketogenesis, and to changes in the pathway kinetics due to post-translational modifications like acetylation [191]. TCA cycle enzymes and selected respiratory chain subunits were included in the proteomics, but were not altered in any of the MCAD groups compared to the controls (Additional File 14: Figure S9). Alternatively, the lack of symptoms in ED patients may be due to their early detection and subsequent dietary advice, which might have interfered with the natural disease development.

Finally, the availability of fibroblast proteomics from only a single clinically certain asymptomatic individual complicates the statistical interpretation of the personalised model predictions. The limited availability of patients in this category is due to the rarity of the disease and the early detection by newborn screening, which was introduced in 2007 [19]. We are dependent on historical samples, as interventions introduced after detection by newborn screening can interfere with natural disease progression. This is in line with the development of so-called ‘N of 1’ trials for rare diseases [192], which may in the future also benefit from computational models. We could partially address the lack of asymptomatic replicates by comparing the results of the asymptomatic individual to the confidence intervals belonging to other clinical groups.

### Towards the digital twin

We have shown how a kinetic model can be used to investigate the pathophysiology of an inborn error of metabolism (IEM). It can not only be used to identify theoretical sources of phenotypic heterogeneity but—together with phenotypical knowledge—it can also be personalised to predict individual phenotypes. In this respect, kinetic models are complementary to genome-scale metabolic models (GEMs; [193, 194]). In contrast to the latest version of the GEM Recon3D [154], which maps more than 230 IEMs, kinetic models focus on specific pathways and contain fewer enzymes and reactions. For this study, however, a kinetic model is better suited, as it allows the explicit simulation of metabolite concentrations and the phenomenon of sequestration of CoA in acyl-CoA esters [25, 27]. Metabolite partitioning can also be intuitively included into a model with explicit metabolite concentrations. Also, the compensatory role of SCAD in MCADD is better represented by a kinetic model, since it depends on the relative affinities of the enzymes for their substrates, as quantified by the *K*_m_ values. Steps have been taken towards enzyme-constrained GEM models, incorporating some of the detail of kinetic models into large networks [193].

The use of personalised computational models to stratify patients requires that these models become increasingly accurate and comprehensive. This highlights the importance of systematically measuring and reporting enzyme kinetics—an endeavour which is facilitated by databases and consortia such as SABIO-RK and STRENDA DB [195, 196]. Advances in enzymology will help to further establish accurate kinetic descriptions of individual reactions, leading to more clinically relevant computational models.

A limitation of this study is that the model only comprises the mitochondrial fatty acid oxidation. An extension of the model with gluconeogenesis, TCA cycle and ketogenesis would introduce an explicit link to the clinical phenotype of hypoketotic hypoglycaemia [29–31, 33, 34]. In an extended model, we expect that the decline of the fatty acid oxidation flux reduces the production of ATP as an important source of Gibbs energy for gluconeogenesis and the production of acetyl-CoA as a precursor for ketogenesis. Moreover, the depletion of CoASH would directly impact other enzymes that require this cofactor. Another important extension could be fatty acid oxidation in peroxisomes and microsomes [197]. The peroxisome can take over the oxidation of some straight long- and medium-chain fatty acids when the mFAO is impaired [198]. Moreover, this extension would make a direct link to diagnostic markers of MCADD, such as dicarboxylic acids [19, 168] and glycine conjugates of fatty acids [19, 168].

Many factors may contribute to phenotypic heterogeneity among people with mFAO disorders, including genetic, post-translational, and environmental differences [2, 3, 199–202]. We considered one of these sources of heterogeneity—changes in enzyme concentrations—and incorporated it into a computational model. This type of personalisation relies on the availability of patient-specific proteomics. Improved measurement techniques and patient-specific in vitro models are important for this. Recent advances in targeted proteomics have allowed for increasingly accurate quantification of the proteome, also from human cells [203–205]. Here we used the QConCAT technology to generate ^13^C-lysine and -arginine labelled peptide standards [109]. The advantage of this method is not only the efficient generation of many standards together but also that it allows to correct for the loss of protein during sample treatment. The limitation is, however, that the set of measured proteins is predetermined and cannot be flexibly adapted. Here, for instance, analysis of the various ACOTs would have been of interest, but these are not present in the applied mitochondrial panel. With respect to in vitro models, this study was based on fibroblast proteomics, but ideally, a computational model of liver metabolism would be personalised with liver proteomics. Since direct biopsies from patient livers are too invasive, more representative in vitro models of MCADD patients and other IEM patients have to be generated. One promising way forward is the generation of induced pluripotent stem cells (iPSCs) from cells collected in less invasive ways, like fibroblasts from skin grafts [206]. These iPSCs can be differentiated into liver organoids, which, if cultured under appropriate physiological conditions, would be a better representation of a native liver [207–210]. A limitation of current hepatic organoid cultures is, however, that they produce little or no glucose, and therefore lack an important clinical readout [211–213]. Finally, the patient fibroblasts used in this study were historical, which complicated phenotypical assessment, since the conditions that triggered metabolic crisis were obviously not controlled. Controlled clinical human studies, such as the Fasting Tolerance in MCADD Infants study [214], are necessary to generate (patho)physiologically relevant patient data under standardised conditions and to increase the statistical power of personalised modelling.

Eventually, advances in personalised kinetic modelling hold the promise of increasingly accurate in silico representations of individual patients. Such ‘digital twins’ of increasing scope and complexity [5, 7, 8] would capture the interaction of a pathogenic mutation embedded within the biochemical networks that determine the trajectory of disease development [3, 215]. This would allow researchers to non-invasively simulate patient outcomes under a variety of perturbations. These simulations would aid in diagnosis, prognosis and predictions of treatment efficacy in a precise and person-specific way.

## Conclusions

In this paper, we presented—to our knowledge—the first computational, kinetic model of mFAO in human liver, and certainly, the first in which differential metabolite localisation due to differences in water solubility has been explicitly addressed. Model simulations identified the risk of a simultaneous reduction in pathway flux and a depletion of CoASH in MCADD. In theory, upregulation of SCAD, MTP and/or the ACOTs are possible rescues to preserve pathway flux and CoASH. Indeed, proteomics analysis of individual patients’ cells revealed the upregulation of SCAD and MTP in an asymptomatic patient. This was also the first time that patient data were directly employed to personalise computational models for the investigation of MCADD. Increased SCAD as a rescue for MCADD has also not been explicitly reported before. Altogether, this study shows the potential and direction of personalised computational modelling for unravelling disease evolution, stratifying risk among patients, and, in future, testing interventions.

## Methods

### Generation of HepG2 MCAD-KO cell line

ACADM was knocked out in HepG2 cells (order number: ATCC-HB-8065, ATCC, Manasses, Virginia, USA) by CRISPR-Cas9, with guide sequence 5′-GGGGTTCGGGCGATGCTGCA-3′, which was designed using the online CRISPR Design Tool [216]. The sequence of the T7 promoter is added 5′ of the guide sequence and additional nucleotides are included for cloning into a pX459 vector (pSpCas9(BB)-2A-Puro) [164]. These oligonucleotides were ordered from Invitrogen (Waltham, Massachusetts, USA). Correct insertion was confirmed by Sanger sequencing (GATC Biotech, Ebersberg, Germany). The HepG2 cells were transfected with the sgRNA-coding plasmids using Lipofectamine 3000 according to the manufacturer’s instructions [217].

### Human fibroblasts

Fibroblasts of patients without a documented heritable metabolic disease (*n* = 5; *control*) and fibroblasts of patients with MCADD were obtained from the Department of Genetics of the University Medical Centre Groningen. All patients with MCADD were homozygous for c.985A > G missense mutation in the *ACADM* gene and were born before neonatal screening for MCADD was implemented in the Netherlands (< 2007). Three subgroups were distinguished. *Symptomatic* (*n* = 4), patients that suffered from at least one recorded metabolic crisis leading to hospitalisation with hypoglycaemia (< 2.6 mmol/L), coma and/or seizures (*n* = 4). *Early diagnosis* individuals (*n* = 5; abbreviated *ED*), siblings of the symptomatic patients who were diagnosed during proband follow-up (ages 0–11 years), were detected and subsequently received preventative dietary advice. *Asymptomatic* (*n* = 1) was discovered well into adulthood (30 years of age), without having ever had noticeable symptoms. Case characteristics are given in Additional File 15: Table S4.

### Genotyping

Genomic DNA was isolated and part of the MCAD gene was amplified by PCR using forward (CTGGCAGCTCTTCTCAAAGC) and reverse (TTCAAGGAGTAGCTGCTC) primers. The PCR product of 350 bp was subcloned into pZERO-blunt and at least 7 colonies per cell line were genotyped by Sanger sequencing.

### Immunoblotting

Western blotting was performed according to a previously described method [218]. GAPDH was used as a positive control. Commercially available antibodies were used to detect GAPDH (AB8245, Abcam, Cambridge, UK) and MCAD (AB92461, Abcam, Cambridge, UK). The original images can be found in Additional File 16: Fig. S10.

### Proteomics

Concentrations of mFAO enzymes and carrier proteins were quantified by liquid chromatography coupled to mass spectrometry in selected reaction monitor mode (LC–MS-SRM), with ^13^C-lysine and -arginine labelled QConCATs targeting a panel of mitochondrial proteins according to [109].

### Acylcarnitine analysis in MCADD patients’ urine

Seven MCADD patients were included, all of whom were homozygous for the c.985A > G missense mutation in the *ACADM* gene and identified before the introduction of MCADD to the Dutch newborn screening. The median age at inclusion was 29 years (range 14–76). The MCADD patients were retrospectively categorised as symptomatic (*n* = 3) or asymptomatic (*n* = 4) according to the same criteria used for the patient fibroblasts. For all patients, acylcarnitines were measured according to Derks et al. [176] in urine obtained after an overnight fast (*fasting*), and in the first urine after breakfast (*fed*). For the symptomatic patients, historic urine samples taken during a first metabolic derangement (at ages 11–13 months) (*crisis*) were retrieved from storage (stored at − 80 °C) and measured.

### Informed consent

For the use of patient urine for the measurement of acylcarnitines, written consent was obtained.

For the use of historical patient fibroblasts, the Medical Ethical Committee of the University Medical Centre Groningen confirmed that according to Dutch law, the Medical Research Involving Human Subjects Act (WMO) does not apply to this study and that an official approval by the Ethical Committee was not required (METc 2016/590).

### Cell culture

Cell cultures were kept at 37 °C and 5% CO_2_. Wild-type and MCAD-KO HepG2 cells were cultured in Dulbecco’s modified Eagle’s medium with 1 g.L^−1^ glucose, 3.7 g.L^−1^ NaHCO_3_, 0.11 g.L^−1^ sodium pyruvate, and amino acids (catalogue number P04-01500, PAN Biotech™, Aidenbach, Germany) with glutamine and NaCl added to a final concentration of 3 mM, supplemented with 10% foetal calf serum (FCS, Gibco™). Assays were performed only on cells that had been passaged fewer than 20 times. Human fibroblasts were cultured in Ham’s F-10 Nutrient Mix (Thermo Fisher Scientific 11550,043), supplemented with 10% foetal calf serum (FCS, Gibco™), 1% penicillin/streptomycin (PenStrep, Gibco™). Seventy-two hours before harvesting the cells, the medium was changed and supplemented with 0.4 mmol.L^−1^ L-carnitine.

### High-resolution respirometry

HepG2 cells were detached by trypsinisation (0.25% Trypsin–EDTA, Gibco™). After 10 min, the trypsin was inactivated by washing with cell culture medium containing 10% FCS. O_2_ consumption flux was then measured in a miRO5 buffer containing 25 μM palmitoyl-CoA, 2 mM L-carnitine and 2 mM malate as the substrates, using an Oroboros Oxygraph-2 k (O2k, Oroboros Instruments, Austria) as described by Van Zutphen et al. [219]. Instead of an ADP-generating system, we directly added 1 mM of ADP. For normalisation to cellular protein, cells were sonicated twice at 20 kHz, 40% amplitude for 30 s (VCX130, Sonics & Materials Inc., Newton, CT., USA). Protein was quantified in the lysate with the BCA protein assay kit (Pierce, Thermo Fisher Scientific Inc., Rockford, IL., USA). Each point in the data set represents one biological repeat.

### Computational model

Model construction and analysis were performed in Wolfram Mathematica version 12.1. Ordinary differential equations (ODEs) were solved with the NDSolve function. Steady states were then calculated by the Newton–Raphson method as the point at which the time derivatives in all ODEs equalled zero (FindRoot function with *MaxIterations—*> *Infinity*), with the end point of the time evolution as an initial guess. All inspected steady states fulfilled the criterion that time derivatives were close to zero (< 10^−10^). Varying the initial metabolite concentrations did not result in alternative steady states. Where metabolic control analysis (MCA) was performed, the summation theorem was satisfied in all cases, confirming the internal consistency of the analysis. Thus, the sum of all flux control coefficients equals unity and the sum of all concentration control coefficients equals zero [139]:

$$\sum_i\mathrm{Flux}\;\mathrm{control}\;{\mathrm{coefficient}}_i=1$$

$$\sum_i\mathrm{Concentration}\;\mathrm{control}\;{\mathrm{coefficient}}_i=0$$

A detailed model description is included in Additional File 1: Text S1. In keeping with FAIR data practices, the model is available on the repository JWS Online Biological Systems Modelling [220]. The model can be viewed, downloaded and simulated directly via on JWS Online [221].

To simulate metabolic stress that could lead to a metabolic crisis, the cytosolic palmitoyl-CoA concentration was set to 150 µM, which is approximately the highest concentration of liver cytosolic long-chain acyl-CoAs reached in vivo [150]. All other parameters were set to their default values (Additional File 1: Text S1) unless otherwise noted.

### Personalised models of individual patients

To transform the generic model of human mFAO into personalised models, we made use of the property that the maximal catalytic activity (*V*_max_) of a protein is proportional to its concentration. The *V*_max_ values in the model were retrieved directly from the literature, while the underlying protein concentration was not explicitly quantified. In model personalisation, the mean protein concentration of the control fibroblasts was chosen as the reference value, effectively equal to the implicit protein concentration underlying the model’s *V*_max_ values. The measured protein concentration of each enzyme in an individual’s fibroblasts was normalised to the corresponding reference (${}^{{[E]}_{\mathrm{person}}}\!\left/ \!{}_{{[E]}_{\mathrm{control mean}}}\right.$). The default *V*_max_ values (and the ETF concentration) in the default model were multiplied by this relative fibroblast protein concentration to yield the personalised parameters (Table 4). Proteins that could not be quantified were set to control levels.

**Table 4:** Personalised parameters based on fibroblast proteomics data

**Phenotype**	**Person**	***V***_**max,VLCAD**_	***V***_**max,MCAD**_^**a**^	***V***_**max,SCAD**_^**b**^	***V***_**max,MCKAT**_	***V***_**max,MTP**_	**Total ETF**
**Unit**		**μmol.min**^**−1**^**.mg-mitochondrial-Protein**^**−1**^					**μM**
**Default**		0.076	0.038	0.01668	2.98	0.167	46.0
**Control**	**C103**	0.042	0.037	0.01668	2.82	0.178	27.4
	**C104**	0.066	0.025	0.01668	1.78	0.088	39.8
	**C105**	0.104	0.059	0.01668	5.32	0.243	65.2
	**C106K**	0.110	0.030	0.01668	2.92	0.187	57.5
	**C106W**	0.032	0.038	0.01668	2.07	0.122	28.0
**Symptomatic**	**P1**	0.044	0	0.01668	2.41	0.132	24.7
	**P5**	0.118	0	0.01668	2.22	0.145	48.0
	**P7**	0.059	0	0.01668	4.74	0.181	44.5
	**P8**	0.094	0	0.01668	3.88	0.258	52.5
**Early diagnosis**	**P2**	0.069	0	0.01668	2.73	0.159	39.4
	**P3**	0.058	0	0.01668	1.32	0.097	35.7
	**P4**	0.121	0	0.01668	4.02	0.252	65.7
	**P6**	0.094	0	0.01668	4.03	0.193	70.8
	**P9**	0.096	0	0.01668	2.12	0.166	48.9
**Asymptomatic**	**P10**	0.096	0	0.02776	3.22	0.257	43.6

^a^*V*_max,MCAD_ activity was set to zero in all MCADD patients

^b^*V*_max,SCAD_ was set to the default value for all patients except P10, since the measurements were too close to the limit of detection to be precisely quantified. For P10, however, the SCAD concentration could be concluded to differ from the other persons in the cohort and was adapted

### Statistical methods

The grouped fluxes and mitochondrial CoASH concentrations from the personalised models were compared using Student’s two-tailed *t*-test. Before performing the test, the normality of the data was inspected  using a quantile–quantile plot. This was done for all phenotypic groups, as well as for the phenotypically grouped MCADD models grouped.

### Supplementary Information

**Additional file 1: Text S1.** Model description. Description of the model, its underlying assumptions, a list of its variables, rate equations, ordinary differential equations, parameters, conserved moieties, and initial conditions.

**Additional file 2: Text S2.** Model validation assumptions and calculations. The assumptions and calculations applied to relate the model predictions to measured data for validation purposes.

**Additional file 3: Table S1.** Sanger Sequencing HepG2. Results of genotyping WT HepG2 cells and four MCAD-knockouts.

**Additional file 4: Figure S1.** Confirmation of MCAD-knockout. Western blotting and targeted proteomics.

**Additional file 5: Table S2.** Full patient urine acylcarnitine profiles. Historical urine acylcarnitine data from symptomatic and asymptomatic MCADD patients.

**Additional file 6: Figure S2.** The simulated effect of MCADD on the acylation of CoA in the mitochondrion. Representation of the amount of the CoA pool that is free, or sequestered as acyl-CoAs of various chain lengths.

**Additional file 7: Figure S3.** The effect of different ACAD deficiencies in silico without metabolite partitioning. NADH production flux and mitochondrial CoASH concentration as a function of cytosolic palmitoyl-CoA concentration with metabolite partitioning removed.

**Additional file 8: Figure S4.** The effect of different ACAD deficiencies in silico with fixed mitochondrial CoASH. NADH production flux and mitochondrial CoASH concentration as a function of cytosolic palmitoyl-CoA concentration with a constant CoASH of 600 μM.

**Additional file 9: Figure S5.** Control analysis at low substrate, high malonyl-CoA concentrations, control MCAD activity. Metabolic control analysis showing a case where CPT1 has the highest flux control.

**Additional file 10: Figure S6.** Control analysis at low acetyl-CoA. Metabolic control analysis showing high VLCAD flux control but negative CoASH concentration control.

**Additional file 11: Table S3.** Full sensitivity analysis. Flux and CoASH concentration response coefficients for all model parameters at [palmitoyl-CoA] = 10 µM and 150 µM.

**Additional file 12: Figure S7.** Effect of possible rescues of mitochondrial CoASH and steady-state mFAO flux in a control model. Effect of incremental changes in VLCAD, SCAD, MTP, ACOT, and CPT2 in a control model activity on NADH flux and CoASH concentration.

**Additional file 13: Figure S8.** Possible rescues of steady-state mFAO flux in an MCADD model with fixed mitochondrial CoASH.  Effect of incremental changes in VLCAD, SCAD, MTP, ACOT, and CPT2 activity with a constant CoASH of 600 μM on NADH flux in a control and MCADD model.

**Additional file 14: Figure S9.** Protein concentration per technical replicate. Concentration of mFAO proteins in MCADD and control fibroblasts.

**Additional file 15: Table S4.** Overview of patient characteristics. ID, sex, categorisation, age of onset/diagnosis, and description of MCADD and control individuals from whom fibroblasts were taken.

**Additional file 16: Figure 10.** Western blots for confirming MCAD-KO. Unedited, uncropped Western blots used to confirm the absence of MCAD in the MCAD-KO cell lines.

## Data Availability

All data generated or analysed during this study are included in this published article, its supplementary information files, and publicly available repositories. The datasets supporting the conclusions of this article are available on the FAIRDOM Hub repository [190]. SBML files of models can be downloaded from and simulated on the JWS Online model repository [160, 161, 221].

## Abbreviations

IEM: Inborn error of metabolism
mFAO: Mitochondrial fatty acid β-oxidation
CASTOR: Coenzyme A sequestration, toxicity and redistribution
CoASH: Free coenzyme A
MCAD: Medium-chain acyl-CoA dehydrogenase
MCADD: MCAD deficiency
ED: Early diagnosis
CPT1: Carnitine palmitoyltransferase 1
CPT2: Carnitine palmitoyltransferase 2
CACT: Carnitine acylcarnitine translocase
VLCAD: Very-long-chain acyl-CoA dehydrogenase
SCAD: Short-chain acyl-CoA dehydrogenase
ACAD: Acyl-CoA dehydrogenase
CROT: Crotonase
M/SCHAD: Medium-/short-chain hydroxyacyl-CoA dehydrogenase
MCKAT: Medium-chain ketoacyl-CoA thiolase
MTP: Mitochondrial trifunctional protein
ACOTcs: Acyl-CoA thioesterase (CoA-sensitive)
ACOTcs: Acyl-CoA thioesterase (CoA-insensitive)
ETF: Electron-transferring flavoprotein
