# Supplementary material for: Personalised modelling of clinical heterogeneity between medium-chain acyl-CoA dehydrogenase patients
Source: BMC Biol. 2023 Sep 4;21:184. doi: 10.1186/s12915-023-01652-9 (PMC10478272; doi:10.1186/s12915-023-01652-9)
Supplement: Supplementary file 1 — Additional file 1: Text S1. Model description. Description of the model, its underlying assumptions, a list of its variables, rate equations, ordinary differential equations, parameters, conserved moieties, and initial conditions. [file 12915_2023_1652_MOESM1_ESM.pdf]

Text S1: Model description

Contents

Text S1: Model description ... 1

0 Major changes ... 2

1 Ordinary differential equations ... 2

2 Kinetic rate equations ... 8

3 Moiety conservation expressions ... 21

4 Kinetic parameters ... 22

5 Metabolite partitioning ... 48

References ... 56

0 Major changes

An existing, validated model of rat liver mFAO was taken as starting point (26). We turned to the literature to look for human kinetic parameters for the reactions in the rate model. Long-chain acyl-CoA dehydrogenase (LCAD), which is not expressed in human liver (87,98,109), was omitted. We included three enzymes that were not in the rat model: carnitine acetyl-CoA transferase (CrAT (120)) and two acyl-CoA thioesterase isoenzymes (ACOT (131)). The two ACOTs included a CoASH-sensitive one (ACOTcs, based on the kinetics of ACOT7 and ACOT13) and a CoASH-insensitive (ACOTcs, equivalent to ACOT2). We also included substrate-specificity for CACT and CPT1 for chain-lengths other than C16, to reflect what we know from the enzymology literature (36,142). The latter adjustment also meant that we had to introduce a few new cytosolic metabolites to the model: acyl-CoAs and acylcarnitines shorter than C16.

1 Ordinary differential equations

Fig. 1 represents the reaction scheme of the model. Mathematically, the model constitutes a set of ordinary differential equations (ODEs) that describe the time-dependent change in metabolite concentrations. At a steady state, all time derivatives in the ODEs equal zero.

All enzymes in the pathway catalyse reactions pertaining to multiple chain-lengths. For instance, CPT1 catalyses the reversible transferase reaction of acyl-CoA to acylcarnitine for metabolites with acyl chains containing 16, 14, 12, 10, and 8 carbon atoms. Many metabolites participate in multiple reactions catalysed by different enzymes. For instance, the C16 acylcarnitine (palmitoylcarnitine) in the cytosol can be converted via the reverse CPT1 reaction to palmitoyl-CoA or can be translocated into the mitochondrion by CACT. The ODE for a given metabolite is the sum of the rates of all reactions producing that metabolite in the forward direction minus the rates of all reactions consuming it. We omit stoichiometric factors from the model description for simplicity. All rates are corrected for compartment volume in the ODEs. The solutions to all these differential equations have units of $\mu M.min^{-1}$.

List of ODEs:

Acyl-CoA (cytosol)

$$C14AcylCoACYT'[t] = -\frac{1}{VCYT} \cdot v_{cpt1C14}$$

$$C12AcylCoACYT'[t] = -\frac{1}{VCYT} \cdot v_{cpt1C12}$$

$$C10AcylCoACYT'[t] = -\frac{1}{VCYT} \cdot v_{cpt1C10}$$

$$C8AcylCoACYT'[t] = -\frac{1}{VCYT} \cdot v_{cpt1C8}$$

Acylcarnitine (cytosol)

$$C16AcylCarCYT'[t] = \frac{1}{VCYT} \cdot (v_{cpt1C16} - v_{cactC16})$$

$$C14AcylCarCYT'[t] = \frac{1}{VCYT} \cdot (v_{cpt1C14} - v_{cactC14})$$

$$C12AcylCarCYT'[t] = \frac{1}{VCYT} \cdot (v_{cpt1C12} - v_{cactC12})$$

$$C10AcylCarCYT'[t] = \frac{1}{VCYT} \cdot (v_{cpt1C10} - v_{cactC10})$$

$$C8AcylCarCYT'[t] = \frac{1}{VCYT} \cdot (v_{cpt1C8} - v_{cactC8})$$

$$C6AcylCarCYT'[t] = -\frac{1}{VCYT} \cdot (v_{cactC6})$$

$$C4AcylCarCYT'[t] = -\frac{1}{VCYT} \cdot (v_{cactC4})$$

Acylcarnitine (mitochondrion)

$$C16AcylCarMAT'[t] = \frac{1}{VMAT} \cdot (v_{cactC16} - v_{cpt2C16})$$

$$C14AcylCarMAT'[t] = \frac{1}{VMAT} \cdot (v_{cactC14} - v_{cpt2C14})$$

$$C12AcylCarMAT'[t] = \frac{1}{VMAT} \cdot (v_{cactC12} - v_{cpt2C12})$$

$$C10AcylCarMAT'[t] = \frac{1}{VMAT} \cdot (v_{cactC10} + v_{cratC10} - v_{cpt2C10})$$

$$C8AcylCarMAT'[t] = \frac{1}{VMAT} \cdot (v_{cactC8} + v_{cratC8} - v_{cpt2C8})$$

$$C6AcylCarMAT'[t] = \frac{1}{VMAT} \cdot (v_{cactC6} + v_{cratC6} - v_{cpt2C6})$$

$$C4AcylCarMAT'[t] = \frac{1}{VMAT} \cdot (v_{cactC4} + v_{cratC4} - v_{cpt2C4})$$

Acyl-CoA (mitochondrion)

$$C16AcylCoAMAT'[t] = \frac{1}{VMAT} \cdot (v_{cpt2C16} - v_{vlcadC16} - v_{mcadC16} - v_{acotcsC16} - v_{acotciC16})$$

$$C14AcylCoAMAT'[t]$$
$$= \frac{1}{VMAT} \cdot (v_{cpt2C14} + v_{mtpC16} - v_{vlcadC14} - v_{mcadC14} - v_{acotcsC14} - v_{acotciC14})$$

$$C12AcylCoAMAT'[t]$$
$$= \frac{1}{VMAT} \cdot (v_{cpt2C12} + v_{mtpC14} + v_{mckatC14} - v_{vlcadC12} - v_{mcadC12} - v_{acotcsC12}$$
$$- v_{acotciC12})$$

$$C10AcylCoAMAT'[t] = \frac{1}{VMAT} \cdot (v_{cpt2C10} + v_{mtpC12} + v_{mckatC12} - v_{cratC10} - v_{vlcadC10} - v_{mcadC10} - v_{acotcsC10} - v_{acotciC10})$$

$$C8AcylCoAMAT'[t] = \frac{1}{VMAT} \cdot (v_{cpt2C8} + v_{mtpC10} + v_{mckatC10} - v_{cratC8} - v_{vlcadC8} - v_{mcadC8} - v_{acotcsC8})$$

$$C6AcylCoAMAT'[t] = \frac{1}{VMAT} \cdot (v_{cpt2C6} + v_{mtpC8} + v_{mckatC8} - v_{cratC6} - v_{mcadC6} - v_{scadC6} - v_{acotcsC6})$$

$$C4AcylCoAMAT'[t] = \frac{1}{VMAT} \cdot (v_{cpt2C4} + v_{mckatC6} - v_{cratC4} - v_{mcadC4} - v_{scadC4} - v_{acotcsC4})$$

Enoyl-CoA (mitochondrion)

$$C16EnoylCoAMAT'[t] = \frac{1}{VMAT} \cdot (v_{vlcadC16} + v_{mcadC16} - v_{mtpC16} - v_{crotC16})$$

$$C14EnoylCoAMAT'[t] = \frac{1}{VMAT} \cdot (v_{vlcadC14} + v_{mcadC14} - v_{mtpC14} - v_{crotC14})$$

$$C12EnoylCoAMAT'[t] = \frac{1}{VMAT} \cdot (v_{vlcadC12} + v_{mcadC12} - v_{mtpC12} - v_{crotC12})$$

$$C10EnoylCoAMAT'[t] = \frac{1}{VMAT} \cdot (v_{vlcadC10} + v_{mcadC10} - v_{mtpC10} - v_{crotC10})$$

$$C8EnoylCoAMAT'[t] = \frac{1}{VMAT} \cdot (v_{vlcadC8} + v_{mcadC8} - v_{mtpC8} - v_{crotC8})$$

$$C6EnoylCoAMAT'[t] = \frac{1}{VMAT} \cdot (v_{mcadC6} + v_{scadC6} - v_{mtpC6} - v_{crotC6})$$

$$C4EnoylCoAMAT'[t] = \frac{1}{VMAT} \cdot (v_{mcadC4} + v_{scadC4} - v_{mtpC4} - v_{crotC4})$$

Hydroxyacyl-CoA (mitochondrion)

$$C16HydroxyacylCoAMAT'[t] = \frac{1}{VMAT} \cdot (v_{crotC16} - v_{mschadC16})$$

$$C14HydroxyacylCoAMAT'[t] = \frac{1}{VMAT} \cdot (v_{crotC14} - v_{mschadC14})$$

$$C12HydroxyacylCoAMAT'[t] = \frac{1}{VMAT} \cdot (v_{crotC12} - v_{mschadC12})$$

$$C10HydroxyacylCoAMAT'[t] = \frac{1}{VMAT} \cdot (v_{crotC10} - v_{mschadC10})$$

$$C8HydroxyacylCoAMAT'[t] = \frac{1}{VMAT} \cdot (v_{crotC8} - v_{mschadC8})$$

$$C6HydroxyacylCoAMAT'[t] = \frac{1}{VMAT} \cdot (v_{crotC6} - v_{mschadC6})$$

$$C4HydroxyacylCoAMAT'[t] = \frac{1}{VMAT} \cdot (v_{crotC4} - v_{mschadC4})$$

Ketoacyl-CoA (mitochondrion)

$$C16KetoacylCoAMAT'[t] = \frac{1}{VMAT} \cdot (v_{mschadC16} - v_{mckatC16})$$

$$C14KetoacylCoAMAT'[t] = \frac{1}{VMAT} \cdot (v_{mschadC14} - v_{mckatC14})$$

$$C12KetoacylCoAMAT'[t] = \frac{1}{VMAT} \cdot (v_{mschadC12} - v_{mckatC12})$$

$$C10KetoacylCoAMAT'[t] = \frac{1}{VMAT} \cdot (v_{mschadC10} - v_{mckatC10})$$

$$C8KetoacylCoAMAT'[t] = \frac{1}{VMAT} \cdot (v_{mschadC8} - v_{mckatC8})$$

$$C6KetoacylCoAMAT'[t] = \frac{1}{VMAT} \cdot (v_{mschadC6} - v_{mckatC6})$$

$$C4KetoacylCoAMAT'[t] = \frac{1}{VMAT} \cdot (v_{mschadC4} - v_{mckatC4})$$

2 Kinetic rate equations

Enzymes catalyse the conversion of multiple substrates according to the same rate equation. The kinetic parameters in these equations define the specific characteristics of the reaction of each chain length. Chain length-specificity is denoted by a subscript *n*. Most reactions are modelled as reversible, random-order Michaelis-Menten equations (47). The only exceptions are the reactions catalysed by CACT, which are modelled as ping-pong reactions, and ACOTci and ACOTcs, which are modelled as irreversible Michaelis-Menten reactions. The free fatty acid product of the ACOT reactions does not interact with any of the enzymes in the model and is omitted. In all reactions, competition between different substrates and products of the same enzyme was included as described previously (26), according to the rate equations below.

One important addition is the parameter *contactSiteCPT*, a parameter referring to the fraction of CPT1 and CPT2 that was found at contact sites (58). Although the enrichment of the CACT transporter at these sites is controversial, the role of contact sites in other transport systems (60) and the enrichment of both CPT1 and CPT2 at the contact sites is interpreted by Eaton (11) to suggest a spatial separation of long-chain acylcarnitine entry at the contact sites from acetylcarnitine export elsewhere. This would make sense also in the context of metabolite partitioning (see 5 Metabolite partitioning in this supplement), whereby longer, less soluble compounds tend to move along membranes or diffuse only short distances between consecutive proteins, while shorter, more soluble compounds, like acetylcarnitine, can more easily diffuse over larger intermembrane spaces. Contact sites are dynamic structures dependent on nutritional and environmental conditions (61,62), which is why we chose to represent the fraction of CPT1 and CPT2 located at contact sites as a parameter that modifies the V_{max} of CPT1 and CPT2 and can be varied dynamically. This parameter functionally reduces the expression level of these enzymes to the fraction of them that are located at contact sites, under the

assumption that only this fraction can import β-oxidation substrates and is therefore relevant to the model. The rates calculated using these kinetic equations have units of μmol.min^{-1}.mg-mitochondrial-protein^{-1}.

List of kinetic rate equations:

CPT1 (random-order, reversible, bi-bi Michaelis-Menten reaction with competitive inhibition of acyl-CoA binding by malonyl-CoA)

For n = {8, 10, 12, 14, 16}:

$$vcpt1C_n = \frac{sfcpt1C_n \cdot Vcpt1 \cdot contactSiteCPT \cdot \left(\frac{C_nAcylCoACYT[t] \cdot CarCYT[t]}{K_mcpt1C_nAcylCoACYT \cdot K_mcpt1C_nCarCYT} - \frac{C_nAcylCarCYT[t] \cdot CoACYT[t]}{K_{eq}cpt1C_n \cdot K_mcpt1C_nAcylCoACYT \cdot K_mcpt1CarCYT}\right)}{\left(1 + \frac{CarCYT[t]}{K_icpt1CarCYT} + \frac{CoACYT[t]}{K_mcpt1CoACYT}\right) \cdot \left(1 + \frac{MalCoACYT}{K_iMalCoACYT} + \sum_{n=8}^{n=16}\left(\frac{C_nAcylCoACYT[t]}{K_mcpt1C_nAcylCoACYT} + \frac{C_nAcylCarCYT[t]}{K_mcpt1C_nAcylCarCYT}\right)\right)}$$

CACT (reversible, bi-bi ping-pong reaction)

For n = {4, 6, 8, 10, 12, 14, 16}:

$$vcactC_n = \frac{sf_{cact} \cdot V_{max,f} \cdot \left(C_nAcylCarCYT[t] \cdot CarMAT[t] - \frac{C_nAcylCarMAT[t] \cdot CarCYT[t]}{K_{eq}cact}\right)}{C_nAcylCarCYT[t] \cdot CarMAT[t] + K_{m,C_nAcylCarCYT} \cdot CarMAT[t] \cdot \boldsymbol{A} + K_{m,CarMAT} \cdot C_nAcylCarCYT[t] \cdot \boldsymbol{B} + \frac{V_f}{V_r \cdot K_{eq}cact} \cdot \left(K_{m,CarCYT} \cdot C_nAcylCarCYT[t] \cdot \boldsymbol{C} + CarCYT[t] \cdot \boldsymbol{D}\right)}$$

<u>where:</u>

$$\boldsymbol{A} = \left(1 + \frac{CarCYT[t]}{K_{m,CarCYT}} + \sum_{n=4}^{n=16}\left(\frac{C_nAcylCarCYT[t]}{K_{m,C_nAcylCarCYT}}\right) - \frac{C_nAcylCarCYT[t]}{K_{m,C_nAcylCarCYT}}\right)$$

$$\boldsymbol{B} = \left(1 + \sum_{n=4}^{n=16}\left(\frac{C_nAcylCarMAT[t]}{K_{m,C_nAcylCarMAT}}\right)\right)$$

$$\boldsymbol{C} = \left(1 + \sum_{n=4}^{n=16}\left(\frac{C_nAcylCarCYT[t]}{K_{m,C_nAcylCarCYT}}\right)\right)$$

$$\boldsymbol{D} = \left(K_{m,AcylCarMAT} \cdot \left(1 + \frac{CarMAT[t]}{K_{m,CarMAT}} + \sum_{n=4}^{n=16}\left(\frac{C_nAcylCarMAT[t]}{K_{m,C_nAcylCarMAT}}\right) - \frac{C_nAcylCarMAT[t]}{K_{m,C_nAcylCarMAT}}\right) + C_nAcylCarMAT[t]\right)$$

CPT2 (random-order, reversible, bi-bi Michaelis Menten reaction)

For n = {4, 6, 8, 10, 12, 14, 16}:

$$vcpt2C_n = \frac{sfcpt2C_n \cdot Vcpt2 \cdot contactSiteCPT \cdot \left(\frac{C_nAcylCarMAT[t] \cdot pfC_nAcylCarMAT \cdot CoAMAT[t]}{K_mcpt2C_nAcylCarMAT \cdot K_mcpt2CoAMAT} - \frac{C_nAcylCoAMAT[t] \cdot pfC_nAcylCoAMAT \cdot CarMAT[t]}{K_{eq}cpt2C_n \cdot K_mcpt2C_nAcylCarMAT \cdot K_mcpt2CoAMAT}\right)}{\left(1 + \frac{CoAMAT[t]}{K_mcpt2CoAMAT} + \frac{CarMAT[t]}{K_icpt2CarMAT}\right) \cdot \left(1 + \sum_{n=4}^{n=16}\left(\frac{C_nAcylCarMAT[t] \cdot pfC_nAcylCarMAT}{K_mcpt2C_nAcylCarMAT} + \frac{C_nAcylCoAMAT[t] \cdot pfC_nAcylCoAMAT}{K_mcpt2C_nAcylCoAMAT}\right)\right)}$$

CrAT (random-order, reversible, bi-bi Michaelis Menten reaction with competitive inhibition of acyl-CoA and L-carnitine binding by long-chain acyl-CoAs)

For n = {4, 6, 8, 10}:

$$vcratC_n = \frac{sfcratC_n \cdot Vcrat \cdot \left(\frac{\frac{C_nAcylCoAMAT[t]}{pfC_nAcylCoAMAT} \cdot CarMAT[t]}{K_mcratC_nAcylCoAMAT \cdot K_mcratC_nCarMAT} - \frac{\frac{C_nAcylCarMAT[t]}{pfC_nAcylCarMAT} \cdot CoAMAT[t]}{K_{eq}cratC_n \cdot K_mcratC_nAcylCoAMAT \cdot K_mcratC_nCarMAT}\right)}{\boldsymbol{A} \cdot \boldsymbol{B}}$$

where:

$$\boldsymbol{A} = \left(1 + \frac{CoAMAT[t]}{K_mcratCoAMAT} + \frac{CarMAT[t]}{K_mcratC_nCarMAT} + \sum_{n=10}^{n=16} \frac{\frac{C_nAcylCoAMAT[t]}{pfC_nAcylCoAMAT}}{K_{i2}cratC_nAcylCoAMAT}\right)$$

$$\boldsymbol{B} = \left(1 + \sum_{n=12}^{n=16} \frac{\frac{C_nAcylCoAMAT[t]}{pfC_nAcylCoAMAT}}{K_{i1}cratC_nAcylCoAMAT} + \sum_{n=4}^{n=10} \left(\frac{\frac{C_nAcylCarMAT[t]}{pfC_nAcylCarMAT}}{K_mcratC_nAcylCarMAT} + \frac{\frac{C_nAcylCoAMAT[t]}{pfC_nAcylCoAMAT}}{K_mcratC_nAcylCoAMAT}\right)\right)$$

VLCAD (random-order, reversible, bi-bi Michaelis Menten reaction)

For n = {8, 10, 12, 14, 16}:

$$vvlcadC_n = \frac{sfvlcadC_n \cdot Vvlcad \cdot \left(\frac{C_nAcylCoAMAT[t] \cdot pfC_nAcylCoAMAT \cdot ETF_{ox}}{K_mvlcadC_nAcylCoAMAT \cdot K_mvlcadETF_{ox}} - \frac{C_nEnoylCoAMAT[t] \cdot pfC_nEnoylCoAMAT \cdot ETF_{red}}{K_{eq}vlcadC_n \cdot K_mvlcadC_nAcylCoAMAT \cdot K_mvlcadETF_{ox}}\right)}{\left(1 + \frac{ETF_{ox}}{K_mvlcadETF_{ox}} + \frac{ETF_{red}}{K_mvlcadETF_{red}}\right) \cdot \boldsymbol{A}}$$

<u>where:</u>

$$\boldsymbol{A} = \left(1 + \sum_{n=8}^{n=16}\left(\frac{C_nAcylCoAMAT[t] \cdot pfC_nAcylCoAMAT}{K_mvlcadC_nAcylCoAMAT} + \frac{C_nEnoylCoAMAT[t] \cdot pfC_nEnoylCoAMAT}{K_mvlcadC_nEnoylCoAMAT}\right)\right)$$

$$ETF_{ox} = \left(\frac{ETF_tMAT}{ETF_{\frac{ox}{red}} + 1}\right) \cdot ETF_{\frac{ox}{red}}$$

$$ETF_{red} = \frac{ETF_tMAT}{ETF_{\frac{ox}{red}} + 1}$$

MCAD (random-order, reversible, bi-bi Michaelis Menten reaction)

For n = {4, 6, 8, 10, 12, 14, 16}:

$$vmcadC_n = \frac{sfmcadC_n \cdot Vmcad \cdot \left(\frac{\frac{C_nAcylCoAMAT[t]}{pfC_nAcylCoAMAT} \cdot ETF_{ox}}{K_mmcadC_nAcylCoAMAT \cdot K_mmcadETF_{ox}} - \frac{\frac{C_nEnoylCoAMAT[t]}{pfC_nEnoylCoAMAT} \cdot ETF_{red}}{K_{eq}mcadC_n \cdot K_mmcadC_nAcylCoAMAT \cdot K_mmcadETF_{ox}}\right)}{\left(1 + \frac{ETF_{ox}}{K_mmcadETF_{ox}} + \frac{ETF_{red}}{K_mmcadETF_{red}}\right) \cdot \left(1 + \sum_{n=4}^{n=16}\left(\frac{\frac{C_nAcylCoAMAT[t]}{pfC_nAcylCoAMAT}}{K_mmcadC_nAcylCoAMAT} + \frac{\frac{C_nEnoylCoAMAT[t]}{pfC_nEnoylCoAMAT}}{K_mmcadC_nEnoylCoAMAT}\right)\right)}$$

<u>where:</u>

$$ETF_{ox} = \left(\frac{ETF_tMAT}{ETF_{\frac{ox}{red}} + 1}\right) \cdot ETF_{\frac{ox}{red}}$$

$$ETF_{red} = \frac{ETF_tMAT}{ETF_{\frac{ox}{red}} + 1}$$

SCAD (random-order, reversible, bi-bi Michaelis Menten reaction)

For n = {4, 6}:

$$vscadC_n = \frac{sfscadC_n \cdot Vscad \cdot \left(\frac{\frac{C_nAcylCoAMAT[t]}{pfC_nAcylCoAMAT} \cdot ETF_{ox}}{K_mscadC_nAcylCoAMAT \cdot K_mscadETF_{ox}} - \frac{\frac{C_nEnoylCoAMAT[t]}{pfC_nEnoylCoAMAT} \cdot ETF_{red}}{K_{eq}scadC_n \cdot K_mscadC_nAcylCoAMAT \cdot K_mscadETF_{ox}}\right)}{\left(1 + \frac{ETF_{ox}}{K_mscadETF_{ox}} + \frac{ETF_{red}}{K_mscadETF_{red}}\right) \cdot \left(1 + \sum_{n=4}^{n=6}\left(\frac{\frac{C_nAcylCoAMAT[t]}{pfC_nAcylCoAMAT}}{K_mscadC_nAcylCoAMAT} + \frac{\frac{C_nEnoylCoAMAT[t]}{pfC_nEnoylCoAMAT}}{K_mscadC_nEnoylCoAMAT}\right)\right)}$$

<u>where:</u>

$$ETF_{ox} = \left(\frac{ETF_tMAT}{ETF_{\frac{ox}{red}} + 1}\right) \cdot ETF_{\frac{ox}{red}}$$

$$ETF_{red} = \frac{ETF_tMAT}{ETF_{\frac{ox}{red}} + 1}$$

MTP (random-order, reversible, tri-tri Michaelis Menten reaction)

For n = {6, 8, 10, 12, 14, 16}:

$$vmtpC_n = \frac{sfmtpC_n \cdot Vmtp \cdot (\boldsymbol{A} - \boldsymbol{B})}{\boldsymbol{C} \cdot \boldsymbol{D} \cdot \boldsymbol{E}}$$

<u>where:</u>

$$\boldsymbol{A} = \frac{C_nEnoylCoAMAT[t] \cdot pfC_nEnoylCoAMAT \cdot NADMAT \cdot CoAMAT[t]}{K_mmtpC_nEnoylCoAMAT \cdot K_mmtpNADMAT \cdot K_mmtpCoAMAT}$$

$$\boldsymbol{B} = \frac{AcetylCoAMAT \cdot pfAcetylCoAMAT \cdot C_{n-2}AcylCoAMAT[t] \cdot pfC_{n-2}AcylCoAMAT \cdot NADHMAT}{K_{eq}mtpC_n \cdot K_mmtpC_nEnoylCoAMAT \cdot K_mmtpNADMAT \cdot K_mmtpCoAMAT}$$

$$\boldsymbol{C} = (1 + \frac{AcetylCoAMAT[t] \cdot pfAcetylCoAMAT}{K_mmtpAcetylCoAMAT} + \frac{CoAMAT[t]}{K_mmtpC_nCoAMAT})$$

$$\boldsymbol{D} = (1 + \frac{NADMAT}{K_mmtpNADMAT} + \frac{NADHMAT}{K_mmtpNADHMAT})$$

$$\boldsymbol{E} = (1 + \sum_{n=6}^{n=16}(\frac{C_nEnoylCoAMAT[t] \cdot pfC_nEnoylCoAMAT}{K_mmtpC_nEnoylCoAMAT} + \frac{C_{n-2}AcylCoAMAT[t] \cdot pfC_{n-2}AcylCoAMAT}{K_mmtpC_{n-2}AcylCoAMAT}))$$

$$NADMAT = (\frac{NAD_tMAT}{\frac{NAD^+}{NADH} + 1}) \cdot \frac{NAD^+}{NADH}$$

$$NADHMAT = (\frac{NAD_tMAT}{\frac{NAD^+}{NADH} + 1})$$

CROT (random-order, reversible, uni-uni Michaelis Menten reaction with competitive inhibition of product and substrate binding by acetoacetyl-CoA)

For n = {4, 6, 8, 10, 12, 14, 16}:

$$vcrotC_n = \frac{sfcrotC_n \cdot Vcrot \cdot (\frac{\frac{C_nEnoylCoAMAT[t]}{pfC_nEnoylCoAMAT}}{K_mcrotC_nEnoylCoAMAT} - \frac{\frac{C_nHydroxyacylCoAMAT[t]}{pfC_nHydroxyacylCoAMAT}}{K_{eq}crotC_n \cdot K_mcrotC_nEnoylCoAMAT})}{(1 + \frac{\frac{AcetoacetylCoAMAT[t]}{pfC_4KetoacylCoAMAT}}{K_icrotAcetoacetylCoAMAT} + \sum_{n=4}^{n=16}(\frac{\frac{C_nEnoylCoAMAT[t]}{pfC_nEnoylCoAMAT}}{K_mcrotC_nEnoylCoAMAT} + \frac{\frac{C_nHydroxyacylCoAMAT[t]}{pfC_nHydroxyacylCoAMAT}}{K_mcrotC_nHydroxyacylCoAMAT}))}$$

M/SCHAD (random-order, reversible, bi-bi Michaelis Menten reaction)

For n = {4, 6, 8, 10, 12, 14, 16}:

$$vmschadC_n = \frac{sfmschadC_n \cdot Vmschad \cdot \boldsymbol{A}}{\boldsymbol{B} \cdot \boldsymbol{C}}$$

where:

$$\boldsymbol{A} = (\frac{\frac{C_nHydroxyacylCoAMAT[t]}{pfC_nHydroxyacylCoAMAT} \cdot NADMAT}{K_mmschadC_nHydroxyacylCoAMAT \cdot K_mmschadNADMAT} - \frac{\frac{C_nKetoacylCoAMAT[t]}{pfC_nKetoacylCoAMAT} \cdot NADHMAT}{K_{eq}mschadC_n \cdot K_mmschadC_nKetocylCoAMAT \cdot K_mmschadNADHMAT})$$

$$\boldsymbol{B} = (1 + \frac{NADMAT[t]}{K_mmschadNADMAT} + \frac{NADHMAT[t]}{K_mmschadNADHMAT})$$

$$\boldsymbol{C} = (1 + \sum_{n=4}^{n=16} (\frac{\frac{C_nHydroxyacylCoAMAT[t]}{pfC_nHydroxyacylCoAMAT}}{K_mmschadC_nHydroxyacylCoAMAT} + \frac{\frac{C_nKetoacylCoAMAT[t]}{pfC_nKetoacylCoAMAT}}{K_mmschadC_nKeotacylCoAMAT}))$$

$$NADMAT = (\frac{NAD_tMAT}{\frac{NAD^+}{NADH} + 1}) \cdot \frac{NAD^+}{NADH}$$

$$NADHMAT = (\frac{NAD_tMAT}{\frac{NAD^+}{NADH} + 1})$$

MCKAT (random-order, reversible, bi-bi Michaelis Menten reaction)

For n = {4, 6, 8, 10, 12, 14}:

$$vmckatC_n = \frac{sfmckatC_n \cdot Vmckat \cdot \boldsymbol{A}}{\left(1 + \frac{CoAMAT[t]}{K_m mckatC_n CoAMAT} + \frac{\frac{AcetylCoAMAT}{pfAcetylCoAMAT}}{K_m mckatAcetylCoA}\right) \cdot \left(1 + \sum_{n=2}^{n=14}\left(\frac{\frac{C_n KetoacylCoAMAT[t]}{pfC_n KetoacylCoAMAT}}{K_m mckatC_n KetoacylCoAMAT} + \frac{\frac{C_{n-2}AcylCoAMAT[t]}{pfC_{n-2}AcylCoAMAT}}{K_m mckatC_{n-2}AcylCoAMAT}\right)\right)}$$

<u>where:</u>

$$\boldsymbol{A} = \left(\frac{\frac{C_n KetoacylCoAMAT[t]}{pfC_n KetoacylCoAMAT} \cdot CoAMAT[t]}{K_m mckatC_n KetoacylCoAMAT \cdot K_m mckatC_n CoAMAT} - \frac{\frac{C_{n-2}AcylCoAMAT[t]}{pfC_{n-2}AcylCoAMAT} \cdot \frac{AcetylCoAMAT}{pfAcetylCoAMAT}}{K_{eq} mckatC_n \cdot K_m mckatC_n KetoacylCoAMAT \cdot K_m mckatC_n CoAMAT}\right)$$

ACOTcs (random-order, irreversible, uni-bi Michaelis Menten reaction with competitive inhibition of acyl-CoA binding by free CoASH)

For n = {4, 6, 8, 10, 12, 14, 16}:

$$vacotcsC_n = \frac{sfacotcsC_n \cdot Vacotcs \cdot \frac{\frac{C_nAcylCoAMAT[t]}{pfC_nAcylCoAMAT}}{K_macotcsC_nAcylCoAMAT}}{(1 + \frac{CoAMAT[t]}{K_iacotcsCoAMAT} + \sum_{n=4}^{n=16} \frac{\frac{C_nAcylCoAMAT[t]}{pfC_nAcylCoAMAT}}{K_macotcsC_nAcylCoAMAT})}$$

ACOTci (random-order, irreversible, uni-bi Michaelis Menten reaction)

For n = {10, 12, 14, 16}:

$$vacotci = \frac{sfacotciC_n \cdot Vacotci \cdot \frac{\frac{C_nAcylCoAMAT[t]}{pfC_nAcylCoAMAT}}{K_macotciC_nAcylCoAMAT}}{(1 + \sum_{n=10}^{n=16} \frac{\frac{C_nAcylCoAMAT[t]}{pfC_nAcylCoAMAT}}{K_macotciC_nAcylCoAMAT})}$$

3 Moiety conservation expressions

CoA is not transported across the membrane. L-carnitine crosses the mitochondrial inner membrane via the CACT antiporter, which exchanges a free L-carnitine for every acylcarnitine it imports, and *vice versa* (63,64). Therefore, the total sum of CoA- or carnitine-containing compounds does not change in either compartment. As a result, four moiety conservation expressions could be constructed: cytosolic and mitochondrial L-carnitine, and cytosolic and mitochondrial CoA. No metabolite in this model contains more than one CoA or L-carnitine so these expressions equal the total pool minus the concentration of each sequestering metabolite.

<u>List of moiety conservation expressions:</u>

$$CoACYT[t] = CoACYTt - \sum_{n=8}^{n=16} C_nAcylCarCoA[t]$$

$$CarCYT[t] = CarCYTt - \sum_{n=4}^{n=16} C_nAcylCarCYT[t]$$

$$CarMAT[t] = CarMATt - \sum_{n=4}^{n=16} C_nAcylCarMAT[t]$$

$$CoAMAT[t] = CoAMATt - CoASHseq - AcetylCoAMAT - \sum_{n=4}^{n=16} (C_nAcylCoAMAT[t] + C_nEnoylCoAMAT[t] + C_nHydroxyacylCoAMAT[t] + C_nKetoacylCoAMAT[t])$$

Note: CoASHseq *is the amount of CoA sequestered outside the mFAO, e.g. in succinyl-CoA and propionyl-CoA. This is a constant value in each model which can be varied between models. In most cases, we selected 1.8 mM (see kinetic parameter list).*

4 Kinetic parameters

All simulation results shown in the main text originate from the parameter set detailed in **Table S1.1.** CPT1 to **Table S1.14.** Boundary conditions below. Some parameters were varied in certain simulations, but this is always explicitly indicated in the corresponding figure legend.

For some parameters, satisfactory values could not be found; in that case, the kinetic parameters from related enzymes were used: due to the unavailability of measured K_m values from human VLCAD for its acyl-CoA substrate, these were assumed equivalent to human LCAD, as previously done (25); also, the K_m of MTP for acetyl-CoA and CoASH were assumed equivalent to that of MCKAT. The K_m values of MTP and MCKAT towards their acyl-CoA products were calculated according to the Haldane relation (65–67). One parameter (the V_{max} of SCAD) was not taken from a single literature source, but from within the range of values from different sources.

Previous models of the mFAO used a single equilibrium constant (K_{eq}) for each enzyme (26,68,69). Since K_{eq} values depend on the acyl chain lengths of the metabolites, which changes as they are shortened in each successive β-oxidation cycle, K_{eq} values were specific for each chain length. To this end, we calculated the K_{eq} value using eQuilibrator, an online tool (66,67). However, eQuilibrator could not calculate the K_{eq} for the transport reaction, CACT, so we opted for a different database, Von Bertalanffy 2.0 (70). For the ACAD enzymes, one of the substrate-product pairs is the $FAD^+/FADH_2$-prosthetic group attached to a large carrier protein (ETF), which is not explicitly contained in eQuilibrator; in that case, measured equilibrium constants were used (71). All K_{eq} values were corrected for temperature based on the Van ‘t Hoff relation (72). In all cases, the criterion of microscopic reversibility was satisfied (73), i.e. equivalent reactions, or sequences of reactions, had the same equilibrium constant (for example, $K_{eq,MTP} = K_{eq,CROT} \cdot K_{eq,MSCHAD} \cdot K_{eq,MCKAT}$). Noteworthy also is that the thermodynamic hurdle between MSCHAD (low K_{eq}) and MCKAT (high K_{eq}) that we previously observed, remained intact, albeit with altered values (27).

For certain parameters, a chain length-specificity is given even though the relevant metabolite has no acyl chain. For example, the K_m of CPT1a when interacting with cytosolic L-carnitine has a unique value related to the chain length of the second substrate participating in the reaction, acyl-CoA. This is because the enzyme follows an ordered binding mechanism by which the acyl-CoA binds first (74–76); the presence of this acyl-CoA in the active site affects the chemistry of L-carnitine's binding to the catalytic pocket, hence leading to chain length-specific K_m values for L-carnitine as well. We do not explicitly model the ordered binding mechanism of these reactions, as previous work has shown that simplification to a generic, random-order bi-bi Michaelis-Menten equation generates equivalent pathway behaviour to more precise rate equations (77). The assumption of random-order binding also allows us to circumvent to use of dissociation constants for these reactions which would be required for modelling an ordered binding reaction accurately and which are nearly totally absent from the literature (47). Wherever ordered binding merits the use of chain length-specific kinetics for free cofactors, a note and a reference will be provided.

Table S1.1. CPT1

Parameter	Chain-length	Value	Unit	Source
**sfcpt1**	C16	1.0	-	(82)
	C14	1.29		
	C12	2.72		
	C10	0.81		
	C8	1.74		
**Vcpt1**		0.872	$\mu mol.min^{-1}.mg\text{-}mitochondrial\text{-}Protein^{-1}$	(81) ***, ****
**Kmcpt1 AcylCoACYT**	C16	12.2	µM	(142)
	C14	30.8		
	C12	11		
	C10	16.7		
	C8	22.9		
**Kmcpt1 CarCYT *** **	C16	85	µM	(81)
	C14	85		Assumed equal to C16
	C12	85		
	C10	85		
	C8	85		
**Kicpt1 CarCYT**		85	µM	Assumed equivalent to K_m from (81)
**Kmcpt1 AcylCarCYT**	C16	123	µM	(142)
	C14	377		
	C12	631		
	C10	885		
	C8	1139		
**Kmcpt1 CoACYT**		40	µM	(75)
**Kmcpt1 MalCoACYT**		9.1	µM	(80)

**Keqcpt1**	C16	0.473	-	Calculated using eQuilibrator (66)
	C14	0.473		
	C12	0.473		
	C10	0.473		
	C8	0.473		
**** Converted from fibroblast protein to hepatocyte protein: (10Log(iBAQ) – liver) / (10Log(iBAQ) – fibroblast) for CPT1a expression yielded a ratio of 100:1*** (79)<br>***** Converted to mitochondrial protein by multiplying by a factor 4*** (78)<br>****** Ordered binding mechanism with acyl-CoA binding first*** (74–76)				

Table S1.2. CACT

Parameter	Chain-length	Value	Unit	Source
**sfcact**	C16	0.63	-	Value for C16 (83)
	C14	0.63		
	C12	0.63		
	C10	1.0		Value for C8 (83)
	C8	1.0		
	C6	1.0		
	C4	0.87		Value for C2 (83)
**Vfcact**	C8	2.34	$\mu mol.min^{-1}.mg$-mitochondrial-Protein^{-1}	(83)
**Vrcact**		2.34		Calculated using Haldane relation
**Kmcact AcylCarCYT**	C16	15	µM	(68)
	C14	15		
	C12	15		
	C10	15		
	C8	15		
	C6	15		
	C4	15		
**Kmcact CarMAT**		130	µM	
**Kmcact AcylCarMAT**	C16	15	µM	
	C14	15		
	C12	15		
	C10	15		
	C8	15		
	C6	15		

	C4	15		
**Kmcact CarCYT**		130	µM	
**Keqcact**	C16	1.0	-	Estimated using Von Bertalanffy 2.0 (70)
	C14	1.0		Assumed equal to C16 value (70)
	C12	1.0		
	C10	1.0		
	C8	1.0		
	C6	1.0		
	C4	1.0		

Table S1.3. CPT2

Parameter	Chain-length	Value	Unit	Source
**sfcpt2**	C16	1.0	-	(82)
	C14	0.66		
	C12	1.26		
	C10	0.70		
	C8	1.07		
	C6	0.16		
	C4	0.01		Assumed near-zero value
**Vcpt2**		0.28	μmol.min^{-1}.mg-mitochondrial-Protein^{-1}	(142) ***, ****
**Kmcpt2 AcylCarMAT**	C16	67	µM	(86)
	C14	42		
	C12	91		
	C10	100		
	C8	1180		
	C6	2000		Assumed an increasing trend as chain-lengths shorten
	C4	5000		
**Kmcpt2 CoAMAT**		1400	µM	(85)
**Kmcpt2 AcylCoAMAT**	C16	12.2	µM	(142)
	C14	30.8		
	C12	11		
	C10	16.7		
	C8	22.9		
	C6	151.5		
	C4	300		Inferred as 2x the value for C6
**Kmcpt2 CarMAT ***	C16	386	µM	(142)
	C14	593		
	C12	400		

	C10	1000		
	C8	5014		
	C6	8000		Arbitrary high value.
	C4	8000		
**Kicpt2 CarMAT**		1486	µM	(80)
**Keqcpt2**	C16	1.928	-	Calculated using eQuilibrator (66)
	C14	1.928		
	C12	1.928		
	C10	1.928		
	C8	1.928		
	C6	1.928		
	C4	1.928		

**** Converted to forward activity by multiplying by a factor 10*** (84)
***** Converted to mitochondrial protein by multiplying by a factor 4*** (78)
****** Ordered binding mechanism with acyl-CoA binding first*** (74)

Table S1.4. CrAT

Parameter	Chain-length	Value	Unit	Source
**sfcrat**	C10	0.33	-	(120)
	C8	0.50		
	C6	1.2		
	C4	1.0		
**Vcrat**		0.06	$\mu mol.min^{-1}.mg\text{-}mitochondrial\text{-}Protein^{-1}$	(120)
**Kmcrat AcylCoAMAT**	C10	64.0	µM	(120)
	C8	50.3		
	C6	54.9		
	C4	43.5		
	C2	21.3		
**Kmcrat CarMAT ***		130	µM	(120) **
**Kmcrat AcylCarMAT**	C10	670	µM	(86)
	C8	1270		
	C6	2510		
	C4	600		
	C2	660		
**Kmcrat CoAMAT ***		88.8	µM	(91)
**Ki1crat AcylCoAMAT**	C16	0.43	µM	(89)
	C14	1.5		
	C12	3.4		
**Ki1crat AcylCoAMAT**	C16	8.0	µM	(90)
	C14	4.2		(89)
	C12	10		
	C10	12		
**Keqcrat**	C10	0.519	-	Calculated using eQuilibrator (66)

	C8	0.519		
	C6	0.519		
	C4	0.519		

**** Random-order binding mechanism, so no chain length-specific KmcratCoAMAT or KmcratCarMAT*** (88,89)
***** Average of chain length-specific values reported in*** (120)

Table S1.5. VLCAD

Parameter	Chain-length	Value	Unit	Source
**sfvlcad**	C16	1.0	-	(93)
	C14	0.511		
	C12	0.2		(97)
	C10	0.2		
	C8	0.1		
**Vvlcad**		0.076	µmol.min^{-1}.mg-mitochondrial-Protein^{-1}	(97)
**Kmvlcad AcylCoAMAT**	C16	14	µM	(96)
	C14	10		
	C12	7		
	C10	10		
	C8	8		
**Kmvlcad ETFox**		0.83	µM	(95)
**Kmvlcad EnoylCoAMAT**	C16	0.377	µM	Average from (93,94)
	C14	0.377		
	C12	0.377		
	C10	0.377		
	C8	0.377		
**Kmvlcad ETFred**		24.2	µM	(69)
**Keqvlcad**	C16	8.27	-	(71)
	C14	8.27		
	C12	8.27		
	C10	8.27		
	C8	8.27		

Table S1.6. MCAD

Parameter	Chain-length	Value	Unit	Source
**sfmcad**	C16	0.17	-	(92)
	C14	0.22		
	C12	0.58		
	C10	0.59		
	C8	1.00		
	C6	1.05		
	C4	0.20		
**Vmcad**		0.038	μmol.min^{-1}.mg-mitochondrial-Protein^{-1}	(97) *, **
**Kmmcad AcylCoAMAT**	C16	23.8	μM	(92)
	C14	10		
	C12	9.3		
	C10	9.1		
	C8	8		
	C6	21.6		
	C4	71.4		
**Kmmcad ETFox**		3.4	μM	(92)
**Kmmcad EnoylCoAMAT**	C16	0.153	μM	(94)
	C14	0.153		
	C12	0.153		
	C10	0.153		
	C8	0.153		
	C6	0.153		
	C4	0.153		
**Kmmcad ETFred**		24.2	μM	(69)
**Keqmcad**	C16	8.27	-	(71)

	C14	8.27		
	C12	8.27		
	C10	8.27		
	C8	8.27		
	C6	0.728		
	C4	0.728		

**** Converted to mitochondrial protein by multiplying by a factor 4*** (78)
***** PalCoA/OctCoA dehydrogenase activity = 2.0*** (97)

Table S1.7. SCAD

Parameter	Chain-length	Value	Unit	Source
**sfscad**	C6	0.542	-	(100)
	C4	1.0		
**Vscad**		0.01668	μmol.min^{-1}.mg-mitochondrial-Protein^{-1}	Chosen from the range 0.00834 – 0.213 (92,101–103) *, **, ***, ****.
**Kmscad AcylCoAMAT**	C6	4.4	μM	(100)
	C4	0.38		
**Kmscad ETFox**		4.1	μM	(92)
**Kmscad EnoylCoAMAT**	C6	0.5	μM	(99)
	C4	0.5		
**Kmscad ETFred**		24.2	μM	(69)
**Keqscad**	C6	0.728	-	(71)
	C4	0.728		

**** Value from*** (92) ***converted to mitochondrial protein by multiplying by a factor 4*** (78)
***** Value from*** (92) ***multiplied by 3.5 according to the purification factor of the enzyme during ammonium sulphate precipitation*** (97)
****** Value from*** (92) ***divided by 2: specific activity with PMS as primary electron acceptor gives about 2x the activity of what you see with the natural primary acceptor, ETF.***
******* The final parameter was the reported value from*** (92) ***multiplied by 2.***

Table S1.8. MTP

Parameter	Chain-length	Value	Unit	Source
**sfmtp**	C16	1.00	-	(114) **, ***
	C14	0.97 *		
	C12	0.93		
	C10	0.81 *		
	C8	0.68		
	C6	0.34 *		
**Vmtp**		0.1656	$\mu mol.min^{-1}.mg\text{-}mitochondrial\text{-}Protein^{-1}$	(113) *, ****, *****, ‡
**Kmmtp EnoylCoAMAT**	C16	102.05	µM	(112)
	C14	102.05		Assumed same as C16
	C12	102.05		
	C10	102.05		
	C8	102.05		
	C6	110		(111)
**Kmmtp NADMAT**	C16	60	µM	(110)
**Kmmtp CoAMAT ‡‡**	C16	28.6	µM	(108)
	C14	33.5		
	C12	38.4		
	C10	35.7		
	C8	35.5		
	C6	18.9		
**Kmmtp AcylCoAMAT**	C14	12288.1	µM	Calculated using Haldane equation (107) ‡‡‡
	C12	14627.7		
	C10	20020.1		
	C8	19550.9		
	C6	22703.3		
	C4	33931.3		

**Kmmtp NADHMAT**		50	µM	(110)
**Kmmtp AcetylCoAMAT**		250	µM	(106)
**Keqmtp**	C16	1280	-	Calculated using eQuilibrator (66)
	C14	1280		
	C12	1280		
	C10	1280		
	C8	1280		
	C6	840		

***** Interpolated as the mean of surrounding values.***

***** MTP has three activities: enoyl-CoA hydratase (ECH), hydroxyacyl-CoA dehydrogenase (HACD), and ketoacyl-CoA thiolase (KACT) each with its own substrate specificity*** (59,110,114)***. Hydroxyacyl-CoA dehydrogenase activity is the lowest*** (56,57,110,114)***. Substrate inhibition can occur within MTP*** (105)***, so we assume the lowest activity is rate-limiting, as also stated by other authors*** (115)***. Therefore, we use HACD's substrate specificity and*** V_{max} ***as for MTP.***

****** These specificities were determined based on reverse HACD activity. Though there have been reports of differing substrate specificities in the forward and reverse direction*** (104)***, we assume they are the same. The agreement in trends between forward and reverse specificities seen in the table is taken as confirmation of the approximate validity of our assumption.***

******* Converted to mitochondrial protein by multiplying by a factor 4*** (78)

******** Converted to forward activity by dividing by 10*** (115)***, assuming HACD is the rate-limiting activity***

‡ Multiplied by 2 according to the observation that contact with its anchoring protein increases HACD activity two-fold

‡‡ Ping-pong binding mechanism for KACT with ketoacyl-CoA binding first, which renders the binding affinity of the free CoASH dependent on the ketoacyl-CoA chain length that bound first (106)***.***

‡‡‡ Assume a V_r/V_f ratio for KACT = 0.095 (106)**, formula:** $K_{m,MTP,C_{n-2}AcylCoA} = K_{eq,MTP} \cdot (\frac{V_{MTP,r}}{V_{MTP,f}})^2 \cdot \frac{K_{m,MTP,C_nKetoacylCoA} \cdot K_{m,MTP,C_nCoA}}{K_{m,MTP,AcetylCoA}}$

Table S1.9. CROT

Parameter	Chain-length	Value	Unit	Source
**sfcrot**	C16	0.01	-	(119)
	C14	0.065		(111)
	C12	0.091		
	C10	0.16		
	C8	0.17		
	C6	0.23		
	C4	1.00		
**Vcrot**		10	µmol.min^{-1}.mg-mitochondrial-Protein^{-1}	(118) *
**Kmcrot EnoylCoAMAT**	C16	5	µM	*Assumed equal to C14*
	C14	5		(111)
	C12	5		
	C10	7		
	C8	9		
	C6	15		
	C4	30		
**Kmcrot HydroxyacylCoAMAT**	C16	0.35	µM	(117)
	C14	0.35		*Assumed same as C16*
	C12	0.35		
	C10	0.35		
	C8	0.35		
	C6	18.68		*Mean of C16 and C4*
	C4	37		(117)
**Kicrot AcetoacetylCoAMAT**		14	µM	(116)
**Keqcrot**	C16	1.33	-	Calculated using eQuilibrator (66)
	C14	1.33		
	C12	1.33		

	C10	1.33		
	C8	1.33		
	C6	1.49		
	C4	5.21		
***** Converted to mitochondrial protein by multiplying by a factor 4** (78)				

Table S1.10. M/SCHAD

Parameter	Chain-length	Value	Unit	Source
**sfmschad**	C16	0.5	-	(104)
	C14	0.575		
	C12	0.6		
	C10	0.55		
	C8	0.55		
	C6	0.95		
	C4	1.0		
**Vmschad**		2.31	μmol.min^{-1}.mg-mitochondrial-Protein^{-1}	(126) *, **
**Kmmschad HydroxyacylCoAMAT**	C16	1.5	μM	(104)
	C14	1.5		
	C12	1.8		
	C10	1.9		
	C8	1.9		
	C6	15		
	C4	75		
**Kmmschad NADMAT**		34.2	μM	(125)
**Kmmschad KetoacylCoAMAT**	C16	1.3	μM	(104)
	C14	1.3		
	C12	1.8		
	C10	2.3		
	C8	3.1		
	C6	5.7		
	C4	13.8		(125)
**Kmmschad NADHMAT**		0.93	μM	(125)
**Keqmschad**	C16	0.00388	-	Calculated using eQuilibrator (66)

	C14	0.00388		
	C12	0.00388		
	C10	0.00388		
	C8	0.00388		
	C6	0.000825		
	C4	0.00767		

**** Converted to mitochondrial protein by multiplying by a factor 4*** (78)

***** Reverse activity converted to forward activity according to the ratio Vf/Vr = 400/720*** (104)

Table S1.11. MCKAT

Parameter	Chain-length	Value	Unit	Source
**sfmckat**	C16	0.0	-	(110)
	C14	0.41		
	C12	0.78		
	C10	1.33		
	C8	1.65		
	C6	2.04		
	C4	1.00		
**Vmckat**		2.98	μmol.min^{-1}.mg-mitochondrial-Protein^{-1}	(124) *
**Kmmckat KetoacylCoAMAT**	C16	1.1	μM	(108)
	C14	1.2 **		
	C12	1.3		
	C10	2.1		
	C8	3.2		
	C6	6.7		
	C4	12.4		
**Kmmckat CoAMAT**	C16	28.6	μM	(108)
	C14	33.5		
	C12	38.4		
	C10	35.7		
	C8	35.5		
	C6	18.9		
	C4	2.2		
**Kmmckat AcylCoAMAT**	C14	313.3	μM	Calculated using Haldane equation (107) ***
	C12	400.4		
	C10	497.2		
	C8	746.7		
	C6	1131.5		

	C4	3540.6		
	C2	25.5		
**Kmmckat AcetylCoAMAT**		250	µM	(106)
**Keqmckat**	C16	249000	-	Calculated using eQuilibrator (66)
	C14	249000		
	C12	249000		
	C10	249000		
	C8	249000		
	C6	699000		
	C4	23400		

**** Converted to mitochondrial protein by multiplying by a factor 4*** (78)

***** Interpolated: average of adjacent values***

****** Assume a V_r/V_f ratio for KACT = 0.095*** (106)***, formula:*** $K_{m,MCKAT,C_{n-2}AcylCoA} = K_{eq,MCKAT} \cdot (\frac{V_{MCKAT,r}}{V_{MCKAT,f}})^2 \cdot \frac{K_{m,MCKAT,C_nKetoacylCoA} \cdot K_{m,MCKAT,C_nCoA}}{K_{m,MCKAT,AcetylCoA}}$

Table S1.12. ACOTcs *

Parameter	Chain-length	Value	Unit	Source
**sfacotcs**	C16	1.0	-	**ACOT7:** (123)
	C14	0.965		
	C12	0.93		
	C10	0.92		
	C8	0.91		
	C6	0.47		**ACOT13:** (122)
	C4	1.0		
**Vacotcs**		0.002	µmol.min^{-1}.mg-mitochondrial-Protein^{-1}	**ACOT7 + ACOT13:** (131)
**Kmacotcs AcylCoAMAT**	C16	16	µM	**ACOT13:** (122)
	C14	9		
	C12	5		
	C10	4.9		
	C8	26		
	C6	18.2		**ACOT7:** (121)
	C4	26.7		
**Kiacotcs CoAMAT**		9.0	µM	**ACOT13:** (122)

***** Sensitive to inhibition by CoASH. Combination of ACOT7 and ACOT13 activity. From these two enzymes, the largest V_{max} and the smallest K_m is chosen each time. For instance, if ACOT13's K_m for C4-acyl-CoA is lower and ACOT7's K_m for C16-acyl-CoA, then the ACOTcs will have the a $K_{m,C4AcylCoA}$ from ACOT13 and a $K_{m,C16AcylCoA}$ from ACOT7.***

Table S1.13. ACOTci *

Parameter	Chain-length	Value	Unit	Source
**sfacotci**	C16	1.0	-	(128)
	C14	0.99		
	C12	0.8 **		
	C10	0.8 **		
**Vacotci**		0.001	$\mu mol.min^{-1}.mg\text{-}mitochondrial\text{-}Protein^{-1}$	(131)
**Kmacotci AcylCoAMAT**	C16	6	µM	(127)
	C14	3		
	C12	5		
	C10	16		

**** Insensitive to inhibition by CoASH. Equivalent to ACOT2 activity.***

***** Interpolated as mean of C16 and C8 values from*** (128).

Table S1.14. Boundary conditions

Type	Parameter	Value	Unit	Source
**contactSiteCPT**		0.4	**-**	(58)
**ETF**	Total *	46	µM	(137)
	Redox ratio **	7		(136)
**NAD(H)**	Total *	250	µM	(135)
	Redox ratio **	1/7.5		(134)
**Constant metabolites**	C16AcylCoACYT	Varied between 0 and 200	µM	(133)
	C2AcylCoAMAT	700	µM	(132)
	MalCoACYT	10	µM	
**Conserved moieties**	CoACYTt	500	µM	(130)
	CoAMATt ***	3600		(130)
	CoAseq ***	2300		Estimated
	CarCYTt	2000		(130) ‡
	CarMATt	2000		(36)
**Compartment volumes**	VMAT	2.33×10^{-6}	$L.mg\text{-}mitochondrial\text{-}Protein^{-1}$	(129) ****
	VCYT	6.64×10^{-6}		

**** [red] + [ox]***
***** [red] / [ox]***

****** Total CoASH in the mitochondrial mFAO model is calculated as the total mitochondrial CoA minus an estimated amount of CoA that would be sequestered in other pathways, e.g. as propionyl-CoA, succinyl-CoA, etc. The real total CoA available to the model is, therefore, the difference between the parameters CoAMATt – CoAseq***

******* Converted to mitochondrial protein by multiplying by a factor 4*** (78)

‡ Total hepatic L-carnitine, compartment not specified

5 Metabolite partitioning

Theoretical basis

Collision theory states that catalysis depends on the likelihood of an enzyme physically interacting, or *colliding*, with its substrate (138). The likelihood of these collisions depends on the localisation of the metabolite and the enzyme. Since enzymes can be either membrane-bound (CPT1, CACT, CPT2, VLCAD, and MTP) or soluble (SCAD, MCAD, CROT, MSCHAD, MCKAT, both ACOTs, and CrAT), the probability of interaction would be impacted by the frequency with which metabolite escapes from the hydrophobic environment of the mitochondrial inner-membrane into the bulk aqueous matrix.

A tacit assumption in most kinetic models of metabolism is that metabolites are distributed homogeneously within each compartment and, therefore, over the enzymes that act on them (139). In these models – for example (140,141,143,144) – as well as in previous versions of the mFAO model that were based on rat or mouse kinetics (25–27,145), the metabolite concentration that each enzyme "sees" is equal to the total concentration in the compartment, e.g. the mitochondrion or cytosol. This assumption might hold for the soluble phosphate sugars of glycolysis, for instance, but is not necessarily appropriate in mFAO: fatty acids, and their acyl-CoA and acylcarnitine derivatives, can have very different physicochemical properties associated with their acyl-chain length.

Esterified fatty acids are amphipathic molecules with a hydrophobic acyl tail and a charged head group, such as carnitine or coenzyme A (146). The longer the acyl chain, the less water-soluble the molecule. Long-chain acyl-CoAs, furthermore, are known to partition readily into membranes (147–149). This can contribute to non-homogeneous distributions of metabolites in a membraneous compartment like a mitochondrion. Knudsen *et al.* (150) calculated that, if allowed to partition freely into membranes, only ≈1 μM from a total pool of 150 μM of palmitoyl-CoA would remain in solution. This is below palmitoyl-CoA's CMC of 42 μM (151), so for the purposes of our model, we assume that partitioning into the membrane will keep concentrations low enough that micelles are not formed.

The interaction between esterified fatty acids and membranes is driven by the association of their hydrophobic acyl groups: longer-chain acyl-CoAs and acylcarnitines have a greater tendency to membrane-associate than shorter ones (147,152). Requero *et al.* (147) argue that metabolites that are anchored to the membrane are more available for transmembrane transport, as their membrane association prevents them from simply "diffusing back into the cytoplasmic aqueous phase". Similar models have been proposed by which both inner- and outer-membrane-bound acyl-CoAs diffuse laterally from active site to active site, essentially "walking" along the membrane instead of diffusing into the bulk cytoplasmic or mitochondrial aqueous phase (37,38,133). As these long-chain acyl-CoAs are sequentially shortened in the mitochondrion, they become increasingly soluble, predisposing them more and more to diffusion into the matrix. The shortest of them would behave indistinguishably from the soluble phosphate sugars that have been successfully modelled under the homogeneous distribution assumption (139). Based on this, we can imagine a chain length-dependent tendency to partition into the membrane, with a larger fraction of longer-chain metabolites associating with the membrane while shorter chains diffuse more readily into the solvent. The enzymes of the mFAO are also either membrane-bound or soluble (Fig. 1), suggesting a possible mechanism by which local concentrations of metabolites can be different depending on the localisation of the enzyme.

Indeed, the existence of an efficient channel for long-chain mFAO has been demonstrated, and one which is fully localised at the inner-membrane surface. Structural evidence shows physical interaction between the VLCAD, MTP, and some electron transport chain complexes (37,39,115). Moreover, Kerner and Bieber (40) found mFAO enzyme activities contaminating their CPT1 isolates, suggesting a close physical association between CPT1 and the mFAO which could not be completely broken by their purification procedure. The formation of a CACT-CPT2 supercomplex has also been demonstrated, probably with cardiolipin playing an important role (41). Interestingly, MTP (105,112) and the electron transport chain (42) are also

known to interact and colocalise with cardiolipin, further strengthening the argument for a tightly organised mFAO metabolon.

Functional evidence includes a lack of long-chain intermediates during or after oxidation of long-chain acyl-CoAs (43–46), reduced flux and the accumulation of long-chain intermediates when the physical interaction between membrane proteins is interrupted (39,48), the total abrogation of palmitoylcarnitine oxidation by the inhibition of MTP (even though, hypothetically, other enzymes could take over its function (49)), and the strong upstream inhibitory effect on CPT1 of downstream metabolite accumulations in the mFAO (81). All of this suggests the existence of some efficient channel starting with CPT1 and ending with the ETC.

Such a long-chain metabolon would retain the intermediates of multiple rounds of oxidation being “transferred between active sites either by channelling or by ‘surface crawling’ along the matrix-facing side of the inner mitochondrial membrane” (50). This cycle would continue, the intermediate becoming more soluble with each truncation, until “the chain length has decreased far enough for the CoA ester to become a better substrate for the matrix ‘soluble’ β-oxidation system.” One could borrow Stanley and Tubbs’ (51) *leaky hosepipe* metaphor, in which the appearance of intermediates from an otherwise closed channel stems from occasional leakage when the channel is under high substrate pressure. In β-oxidation, the hosepipe becomes increasingly leaky as the substrates are shortened until the acyl group becomes fully soluble.

Compelling evidence of similar medium- and short-chain mFAO metabolons has not been forthcoming, despite suggestions that there may be transient associations between the enzymes responsible for these processes (39,45). This fits with the expectation that smaller, more soluble acyl esters are not localising at a particular spot in the mitochondrion, perhaps due to the ease with which they diffuse into the aqueous matrix.

Based on these insights from the literature, we overlaid a range of metabolite solubilities on top of the kinetics and structure of the model. The solubilities are considered equivalent to a tendency of less soluble mitochondrial metabolites to form pockets of increased concentration in the vicinity of membrane-bound enzymes – and, correspondingly, lower concentrations near soluble enzymes.

Limitations

The partitioning factor described in the main text is an admittedly coarse-grained representation of the effects of metabolite partitioning. We do not, for instance, explicitly distinguish the effect of increased membrane partitioning from the effect of substrate channelling. It might be that enzyme-to-enzyme channelling of pathway intermediates has its own kinetics which does not correspond functionally to the simulated effect of increased local metabolite concentrations. Similarly, increased membrane partitioning of less soluble metabolites does not necessarily equate to increased catalysis, as these localised pockets of increased concentration might not always be co-localised with the enzymes of the metabolon, or might not effectively enter this channel. Moreover, there might be variety in the type of channel between each of the enzymes in the long-chain mFAO metabolon: the VLCAD-MTP interaction (39), for instance, might be qualitatively different from the mechanism that causes CPT1 inhibition by downstream metabolite accumulations (45,81). These, among other things, are complexities that remain to be fully elucidated before they can be modelled with accuracy. What we have done, however, is to include something of the heterogeneity in the chemical characteristics of different fatty acid derivatives. We also showed that this improves some of our model predictions, suggesting that it does capture something of the true effect of differing solubilities.

The multifactorial way in which fatty acid derivatives' behaviour is determined, is further complicated in the cytosol. In the mitochondrion, for instance, the mitochondrial inner membrane, which houses the long-chain metabolon, is the only membrane to which acyl groups can associate. In the cytosol, metabolites might partition into multiple organelles that

compete for substrates with the mitochondrion. The added effect of cytosolic binding proteins – including the acyl-CoA binding protein (ACBP) and fatty acid-binding protein (FABP) – adds another layer of complexity: these proteins have (chain length-specific) nanomolar K_d values for binding to fatty acids, acyl-CoAs, and acylcarnitines (133,150); they compete with each other as well as with other enzymes such as acyl-CoA synthetase (52), and are thought to donate acyl-CoAs directly to CPT1 (53). All of this suggests a multi-faceted picture of fatty acid processing in the cytosol which, for simplicity, we do not attempt to resolve. The apparent absence of binding proteins from the mitochondrion (52,133) allows us to make the simplifying assumption that heterogeneity in mitochondrial concentrations will be purely a function of membrane-association due to insolubility. Therefore, only the rate equations of reactions that are fully mitochondrial (i.e., all except CPT1 and CACT) are modified with metabolite partitioning in our model.

Application

First, we obtained the canonical SMILES specification by searching the trivial name of our model metabolites in PubChem (54). The SMILES specification was then used as input to generate an octanol/water partitioning coefficient using an online tool, ALOGPS (55). We then calculated a *relative partitioning factor* for all mitochondrial acylcarnitines and acyl-CoAs by normalising to that of acetyl-CoA, assuming full water solubility of acetyl-CoA:

$$pf = \frac{P_{solute}}{P_{acetylCoA}}$$

Any compound that was more hydrophilic than acetyl-CoA was assigned a pf of 1, giving it full water solubility.

In the kinetic equations, it can be seen that we used this relative partitioning factor to modify rates by multiplying with concentrations in the rate equation of membrane-bound enzymes (increasing the effective local concentration) and dividing concentrations of soluble/matrix enzymes (decreasing effective local concentrations):

$$[s]_{membrane} = [s] \cdot pf_s$$

$$[s]_{matrix} = \frac{[s]}{pf_s}$$

In this way, the concentration in each compartment remains unaltered, but the rates are determined as if they were exposed to local concentrations. This helps us circumvent the problem of defining the precise volumes within which the local concentrations are located, which would be necessary if we changed the real concentrations in the model, to ensure that mass is conserved. Instead, the relative partitioning factor expresses changes in local concentration only in terms of fold changes in local concentrations when calculating reaction rates.

Coefficient table

Table S1.15. Calculating the relative partitioning factor of L-carnitine- and CoA-esters.

Metabolite	LogP from AloGPS	Partitioning factor $P = \frac{[solute]_{octanol}}{[solute]_{water}}$	Relative partitioning factor $pf = \frac{P_{solute}}{P_{acetylCoA}}$	***Final value*** ***Any pf below 1 is assumed equal to 1 (i.e. fully water-soluble) and all values rounded to one decimal.***
**C2AcylCoA**	-0.58	0.26	1.00	1
**C4AcylCoA**	-0.22	0.60	2.29	2.3
**C4AcylCar**	-2.1	0.01	0.03	1
**C4EnoylCoA**	-0.11	0.78	2.95	3
**C4HydroxyAcylCoA**	-0.62	0.24	0.91	1
**C4KetoacylCoA**	-0.06	0.87	3.31	3.3
**C6AcylCoA**	0.07	1.17	4.47	4.5
**C6AcylCar**	-1.68	0.02	0.08	1
**C6EnoylCoA**	0.2	1.58	6.03	6
**C6HydroxyAcylCoA**	-0.28	0.52	2.00	2
**C6KetoacylCoA**	-0.04	0.91	3.47	3.5
**C8AcylCoA**	0.45	2.82	10.72	10.7
**C8AcylCar**	-1.22	0.06	0.23	1
**C8EnoylCoA**	0.55	3.55	13.49	13.5
**C8HydroxyAcylCoA**	-0.03	0.93	3.55	3.5
**C8KetoacylCoA**	0.23	1.70	6.46	6.5
**C10AcylCoA**	0.87	7.41	28.18	28.2
**C10AcylCar**	-0.85	0.14	0.54	1
**C10EnoylCoA**	0.92	8.32	31.62	31.6
**C10HydroxyAcylCoA**	0.31	2.04	7.76	7.8
**C10KetoacylCoA**	0.55	3.55	13.49	13.5
**C12AcylCoA**	1.35	22.39	85.11	85.1

**C12AcylCar**	0.03	1.07	4.07	4.1
**C12EnoylCoA**	1.41	25.70	97.72	97.7
**C12HydroxyAcylCoA**	0.68	4.79	18.20	18.2
**C12KetoacylCoA**	0.98	9.55	36.31	36.3
**C14AcylCoA**	1.84	69.18	263.03	263
**C14AcylCar**	0.86	7.24	27.54	27.5
**C14EnoylCoA**	1.9	79.43	302.00	302
**C14HydroxyAcylCoA**	1.19	15.49	58.88	58.9
**C14KetoacylCoA**	1.48	30.20	114.82	114.8
**C16AcylCoA**	2.35	223.87	851.14	851.1
**C16AcylCar**	1.77	58.88	223.87	223.9
**C16EnoylCoA**	2.38	239.88	912.01	912
**C16HydroxyAcylCoA**	1.68	47.86	181.97	182
**C16KetoacylCoA**	1.96	91.20	346.74	346.7

References

Refer to reference list in main text.
