# Supplementary material for: Personalised modelling of clinical heterogeneity between medium-chain acyl-CoA dehydrogenase patients
Source: BMC Biol. 2023 Sep 4;21:184. doi: 10.1186/s12915-023-01652-9 (PMC10478272; doi:10.1186/s12915-023-01652-9)
Supplement: Supplementary file 2 — Additional file 2: Text S2. Model validation assumptions and calculations. The assumptions and calculations applied to relate the model predictions to measured data for validation purposes. [file 12915_2023_1652_MOESM2_ESM.pdf]

Text S2: Model validation assumptions and calculations

Contents

Text S2: Model validation assumptions and calculations 1

1 HepG2 O_2 consumption 2

2 Whole-body ketogenic flux 4

3 ACAD activity partitioning 5

4 Including HepG2 proteomics 8

References 11

1 HepG2 O_2 consumption

The model was adjusted to mimic experimental conditions and the simulated NADH production flux was converted to an oxygen consumption flux for direct comparison to respirometry experiments. The resultant model, odendaal2, can be viewed and simulated on JWS Online (160). All model outputs shown are at steady state.

The following steps were taken:

1. Cytosolic volume was replaced by the extramitochondrial volume in the respirometry chamber (55.7 x 10^{-3} L.mg-mitochondrial-protein^{-1}). To estimate this parameter, the average cellular protein measured for the HepG2 cells used in the respirometry experiments was first converted to a mitochondrial protein concentration according to Wiśniewski *et al.*'s (78) observation that mitochondria contain 12% of the total cellular protein in HepG2 cells. Dividing the volume of the experimental chamber, 500 µL (O2k, Oroboros Instruments, Austria) by the mitochondrial protein yields the final chamber volume. An underlying assumption of this conversion is that the mitochondria comprise only a negligible portion of the chamber volume during an assay.

2. Extramitochondrial palmitoyl-CoA was set to 25 µM as in the experiment. The terminal substrate and product concentrations were kept constant to yield a steady-state model, as a tacit assumption of measuring steady-states in an Oroboros is that substrate is in excess and that its concentration changes only negligibly over time.

3. Since ADP is added in excess, reducing equivalent pools would be very oxidised. To mimic this, the most oxidised values retrieved during our parameter search are chosen for the NAD^+/NADH and ETF pools. The NAD^+:NADH ratio is set to 120:1 (162) and the ETF_{ox}:ETF_{red} ratio to 1.7:1 (69).

4. Extramitochondrial L-carnitine was set to a final concentration of 2000 µM as per the experimental protocol.

5. The simulated NADH production flux under these conditions was then recalculated to an oxygen consumption flux. The electron transport due to one mitochondrial NADH consumes 0.5 O_2 and due to one $FADH_2$,0.3 O_2 (cf. the production of 2.5 ATP molecules per NADH and 1.5 ATP molecules per $FADH_2$ consumed, and a P:O ratio of 2.5:0.5) (163).

<u>Mitochondrial β-oxidation:</u>

- For every NADH produced in the mFAO, one $FADH_2$ is also produced;
  - = 0.5 O_2 + 0.3 O_2
  - = 0.8 O_2 consumed per NADH produced in the mFAO

<u>TCA cycle:</u>

- each produced acetyl-CoA can be further oxidised in the TCA cycle to 3 NADH and 1 $FADH_2$;
- 8 acetyl-CoAs are produced by the 7 rounds of oxidation of palmitoyl-CoA;
  - = 8/7 acetyl-CoA are produced alongside every NADH in the mFAO
- only 15% of the acetyl-CoA from the β-oxidation goes on to enter the TCA cycle (29);
- thus, the O_2 consumed due to oxidative phosphorylation linked to the TCA cycle comprises:
  - = (1.5 O_2 + 0.3 O_2)*0.15*8/7
  - = 0.31 O_2 consumed per NADH produced in the mFAO

<u>In total:</u>

- = 0.8 O_2 + 0.31 O_2
- = 1.11 O_2 total consumed per NADH produced in the mFAO

<u>Conversion:</u>

This can be used as a conversion factor for an NADH production flux with units of $\mu mol.min^{-1}.g$-mitochondrial-protein^{-1} to an O_2 consumption flux with the same units.

$$\mathbf{O_2\ \ production\ flux = NADH\ production\ flux\ by\ mFAO\ \times 1.11}$$

2 Whole-body ketogenic flux

Fletcher and colleagues (28) determined the ketone body production flux in 24h-fasted human adults based on stable isotope dilution, normalised to lean body weight (kg_{LBW}). The values were rounded to the nearest multiple of 0.5 (**Table S2.1. Ketogenic flux.**).

Table S2.1. Ketogenic flux.

Set of measured values in $\mu mol.kg_{LBW}^{-1}.min^{-1}$	**Reference**
**{7.5, 13, 15, 15.5, 17, 18, 18, 18, 18, 18, 18, 21, 21, 22.5, 25, 26, 29}**	(28)

We simulated the acetyl-CoA production flux at stress conditions in $\mu mol.min^{-1}.mg$-mitochondrial-protein^{-1}. The result was converted to a ketone-body production flux in $\mu mol.kg_{LBW}^{-1}.min^{-1}$ for comparison to the measured data:

1. First the simulated flux was multiplied by the mitochondrial protein of one hepatocyte (150 pg) to get a rate of acetyl-CoA production in $\mu mol.min^{-1}$ per hepatocyte (78);

2. this was multiplied to liver hepatocellularity to scale the flux to the ketogenesis of all hepatocytes in an adult human liver: 1.5 kg average liver weight (155) x 139 million hepatocytes.g-Liver^{-1} (156) ≈ 200 billion hepatocytes.Liver^{-1};

3. 85% of acetyl-CoA from mFAO in normal liver is condensed to ketone bodies. Multiply the flux by 0.85 to reflect this (29);

4. 4.4% of ketone bodies in 3h-fasted rats came from leucine and much lower fractions from other ketogenic amino acids (157). Based on this, we assumed a 5% contribution of ketogenic amino acids by dividing the simulated flux by 0.95;

5. the participants in this study had an average lean body weight (LBW) of 50 kg (28). Divide by 50 to normalise the flux to kg lean body weight.

Conversion:

$$\text{Ketogenic flux} = \frac{\text{NADHproduction} \times \text{hepatocyte.mg}-\text{mito}-\text{prot}^{-1} \times \text{hepatocytes.liver}^{-1} \times \text{mFAOtoKetones} \times \frac{1}{\text{fractionKetonesFromFats}}}{\text{LBW}}$$

$$\text{Ketogenic flux} = \frac{\text{NADHproduction} \times (150 \times 10^{-9}) \times (200 \times 10^{9}) \times 0.85 \times \frac{1}{0.95}}{50}$$

$$\mathbf{Ketogenic\ flux} = \mathbf{NADH\ production} \times \mathbf{536.842}$$

$$(\mathbf{\mu mol.kg_{LBW}^{-1}.min^{-1}}) = (\mathbf{\mu mol.min^{-1}.mg - mitochondrial - protein^{-1}}) \times \mathbf{536.842}$$

3 ACAD activity partitioning

Aoyama *et al.* (97) collected human liver samples (n=5) and prepared homogenates. In separate batches, anti-VLCAD and anti-MCAD antibodies were used to precipitate out the activity of each of these enzymes. The palmitoyl-CoA dehydrogenation rate was measured in the untreated and anti-VLCAD-treated samples, and the octanoyl-CoA dehydrogenation rate in the untreated and anti-MCAD-treated samples. To mimic these experimental conditions, the following adjustments were made to the default model (the resultant model, odendaal3, can be viewed and simulated on JWS Online (161)):

1. In the assay, the lytic procedure removed the barriers between the cytosolic and mitochondrial compartments. Therefore, we removed all cytosolic variables from the model, as well as the transport reactions facilitated by CACT. The assay compartment volume was then calculated based on Aoyama *et al.*'s (97) observation that 100g human liver crude extract contains 1533 mg total cellular protein. It bears mentioning that this observation was made on a crude homogenate during the purification of VLCAD, for which the tissue was homogenised differently than when the homogenate was generated for dehydrogenation rate measurements. Our assumption, however, is that cellular protein per liver weight remains constant irrespective of the homogenisation process. For the rate measurements, the authors report to have followed the protocol used by Furuta *et al.* (158): they made a crude extract from 2 mg of liver into a final volume of 200 μL; from this, they took 60 μL and diluted it to 1 mL for the acyl-CoA

dehydrogenation assays. Assuming a ratio of mitochondrial to total cellular protein of 1:4 (78), we then calculated a normalised assay volume of 0.4339 $L.mg\text{-}mitochondrial\text{-}protein^{-1}$. Finally, in the model, we merged the concentrations of total CoA, L-carnitine, NAD^+, and NADH in both compartments. To mimic the experimental conditions in the model, we used:

- L-carnitine = 0.02739 µM
- CoA = 30.02 µM (which includes the 30 µM of CoA ester added as substrate)
- NAD^+ = 0.001161 µM
- NADH = 0.0001786 µM

2. Furuta *et al.* (158) used 2,6-dichlorophenolindophenol (DCICP) as the final electron acceptor and phenazine methosulphate (PMS) as an intermediate carrier. Since PMS interacts directly with the ACAD enzymes, we chose to use PMS as cofactor for all VLCAD, MCAD, and SCAD reactions, instead of ETF. 1.6 mM of PMS was added to the assay, which we include in the model as:

$PMS_{ox}[0]$ = 1600 µM.

PMS has different kinetics from ETF when binding to the ACADs. Finocchiaro *et al.* (92) determined K_m values for human liver SCAD and MCAD interacting with oxidised PMS:

$K_{m,SCAD,PMS}$ = 179 µM

$K_{m,MCAD,PMS}$ = 1620 µM

These are considerably different from what they observed for the oxidised ETF:

$K_{m,ETF}$ = 4.1 µM

$K_{m,ETF}$ = 3.4 µM

Ikeda *et al*. (101) also determined K_m values for rat liver ACAD enzymes ranging from 270 to 1200 µM (they do not specify which enzyme yielded which K_m), which confirms that values in

this range are reliable. We assumed the same K_m for the oxidised and reduced PMS, as well as that MCAD and VLCAD have the same K_m values.

3. CPT1 activity was removed from the model entirely as 0.6% Triton X-100 was used to homogenise the tissue samples, and CPT1 is inhibited by detergents (84). This inhibition is probably not full, but we assume that its remaining activity would be negligible in the assay cocktail compared to that of CPT2, which catalyses the same reaction.

4. Since the cells were lysed harshly, all mitochondrial structure was assumed to be abrogated. Consequently, membrane- and matrix-localised metabolite concentrations would no longer play a role. To mimic this, we removed the metabolite partitioning attribute from the model. Partitioning was removed from the basal model by setting all partitioning factors to 1.

5. The model was made dynamic by making acetyl-CoA, NADH, NAD^+, PMS_{ox}, PMS_{red} variable and introducing the 30 µM substrate (C8- or C16-acyl-CoA) as initial values of the variables. The ODEs of these new variables were:

$$AcetylCoA'[t] = v_{mckatC16} + v_{mckatC14} + v_{mckatC12} + v_{mckatC10} + v_{mckatC8} + v_{mckatC6} + v_{mckatC4} + v_{mtpC16} + v_{mtpC14} + v_{mtpC12} + v_{mtpC10} + v_{mtpC8} + v_{mtpC6}$$

$$NADH'[t] = v_{mschadC16} + v_{mschadC14} + v_{mschadC12} + v_{mschadC10} + v_{mschadC8} + v_{mschadC6} + v_{mschadC4} + v_{mtpC16} + v_{mtpC14} + v_{mtpC12} + v_{mtpC10} + v_{mtpC8} + v_{mtpC6}$$

$$NAD^{+'}[t] = -(v_{mschadC16} + v_{mschadC14} + v_{mschadC12} + v_{mschadC10} + v_{mschadC8} + v_{mschadC6} + v_{mschadC4} + v_{mtpC16} + v_{mtpC14} + v_{mtpC12} + v_{mtpC10} + v_{mtpC8} + v_{mtpC6})$$

$$PMS'_{red}[t] = v_{vlcadC16} + v_{vlcadC14} + v_{vlcadC12} + v_{vlcadC10} + v_{vlcadC8} + v_{mcadC16} + v_{mcadC14} + v_{mcadC12} + v_{mcadC10} + v_{mcadC8} + v_{mcadC6} + v_{mcadC4} + v_{scadC6} + v_{scadC4}$$

$$PMS'_{ox}[t] = -(v_{vlcadC16} + v_{vlcadC14} + v_{vlcadC12} + v_{vlcadC10} + v_{vlcadC8} + v_{mcadC16} + v_{mcadC14} + v_{mcadC12} + v_{mcadC10} + v_{mcadC8} + v_{mcadC6} + v_{mcadC4} + v_{scadC6} + v_{scadC4})$$

4 Including HepG2 proteomics

Adjustments for differences in protein expression can be made by making relative adjustments to V_{max} values, which implicitly contain the enzyme concentration:

$$V_{max} = k_{cat} \cdot [E]$$

Note that this includes the assumption that the k_{cat} of the enzyme is not changed by its expression in a different cell type.

HepG2 proteomics was included when simulating O_2 consumption flux. Wiśniewski *et al.* (78) performed proteomics on both human hepatocytes and HepG2 cells. A dimensionless amount of each quantified protein *i* is represented by the formula (78):

$$Protein\ (E_i) = \frac{MS\text{−}signal(E_i)}{Total\ MS\text{−}signal}$$

Where E_i represents the amount a given protein *i*.

A hypothetical concentration could be calculated for each of these proteins using total cellular protein:

$$[E]_{cell} = \frac{E_i}{Cellular\ protein}$$

which would be given in *arbitrary-units.pg-cellular-protein*$^{-1}$. Total cellular protein content was retrieved from (78) as 600 pg in human hepatocytes and 170 pg in HepG2 cells.

Since we are interested in converting a V_{max} per mitochondrial protein, we calculate $[E]_{mito}$ from $[E]_{cell}$ using the fraction of cellular protein located in the mitochondrion according to (78) – 0.25 for hepatocytes and 0.12 for HepG2:

$$[E]_{mito} = \frac{[E]_{cell}}{fraction_{mito}}$$

which has dimensions: *arbitrary-units.pg-mitochondrial-protein*$^{-1}$.

A relative, dimensionless expression factor was calculated which could be directly applied to modify a given V_{max} from a human hepatocyte in terms of mitochondrial protein to a V_{max} from a HepG2 cell in terms of mitochondrial protein:

$$relExpress(E_i) = \frac{[E]_{mito,HepG2}}{[E]_{mito,hepatocyte}}$$

$$relExpress(E_i) = \frac{\frac{E_{i,HepG2}}{HepG2\ protein} \times \frac{1}{fraction_{HepG2,mito}}}{\frac{E_{i,Hepatocyte}}{Hepatocyte\ protein} \times \frac{1}{fraction_{Hepatocyte,mito}}}$$

$$relExpress(E_i) = \frac{E_{i,HepG2}}{E_{i,Hepatocyte}} \times \frac{Hepatocyte\ protein \times fraction_{Hepatocyte,mito}}{HepG2\ protein \times fraction_{HepG2,mito}}$$

$$relExpress(E_i) = \frac{E_{i,HepG2}}{E_{i,Hepatocyte}} \times \frac{600\ pg \times 0.25}{170\ pg \times 0.12}$$

$$relExpress(E_i) = \frac{E_{i,HepG2}}{E_{i,Hepatocyte}} \times \frac{1}{0.136}$$

Which can be calculated for each protein of interest using the $E_{i,HepG2}$ *and* $E_{i,Hepatocyte}$ from Wiśniewski *et al.* (78,159).

These can then be inserted into the model as follows:

$$V_{max,HepG2} = V_{max,Hepatocyte} \times relExpress(E_i)$$

Where multiple subunits from one enzyme were measured, the one with lower relative expression was chosen for the adjustment. This decision was based on the reasoning that one of each subunit would be necessary to form a fully functional protein. All proteins in the modelled pathway indeed contain maximally one of each subunit.

For ACOTcs, which represents the combined activity of ACOT7 and ACOT13, the average relative expression between the two was used. The CACT transporter was not adjusted for expression as it did not occur in the proteomics dataset.

Table S2.1. Calculating the HepG2 adjustment factors.

Protein (peptide)	Average total protein (hepatocyte)	Average total protein (HepG2)	Relative expression factor $\frac{Total\ HepG2\ protein\ (E)}{Total\ hepatocyte\ protein\ (E)} \times \frac{1}{0.136}$
**CPT1A**	0.000293191	7.4523E-05	1.868964192
**CPT2**	0.000941861	0.000157547	1.229941945
**CrAT**	0.000596653	0.00019745	2.433300066
**CACT ***	n/a	n/a	n/a
**VLCAD**	0.001643915	0.000461527	2.064329992
**MCAD**	0.001522881	0.000400706	1.934733932
**SCAD**	0.001492529	0.00023451	1.155311632
**CROT**	0.005800392	0.000769827	0.975881124
**MSCHAD**	0.002105547	0.000845139	2.951372559
**MCKAT**	0.005294834	0.0006484	0.900433693
**MTP (HADHA)**	0.004460333	0.003597818	5.931068919
**MTP (HADHB)**	0.002838857	0.001365954	3.537967351
**ACOTci (ACOT2)**	4.9766×10^{-5}	0	0
**ACOTcs (ACOT7)**	1.71559×10^{-5}	7.28325×10^{-5}	31.21562952
**ACOTcs (ACOT13)**	0.000543734	0.000185642	2.510449592
**ETF (alpha)**	0.002360304	0.001704044	5.308526703
**ETF (beta)**	0.002121134	0.001245063	4.316027441

Not adjusted.

References

Refer to reference list in main text.
