# Supplementary material for: Personalised modelling of clinical heterogeneity between medium-chain acyl-CoA dehydrogenase patients
Source: BMC Biol. 2023 Sep 4;21:184. doi: 10.1186/s12915-023-01652-9 (PMC10478272; doi:10.1186/s12915-023-01652-9)
Supplement: Supplementary file 4 — Additional file 4: Figure S1. Confirmation of MCAD-knockout. Western blotting and targeted proteomics. [file 12915_2023_1652_MOESM4_ESM.pdf]

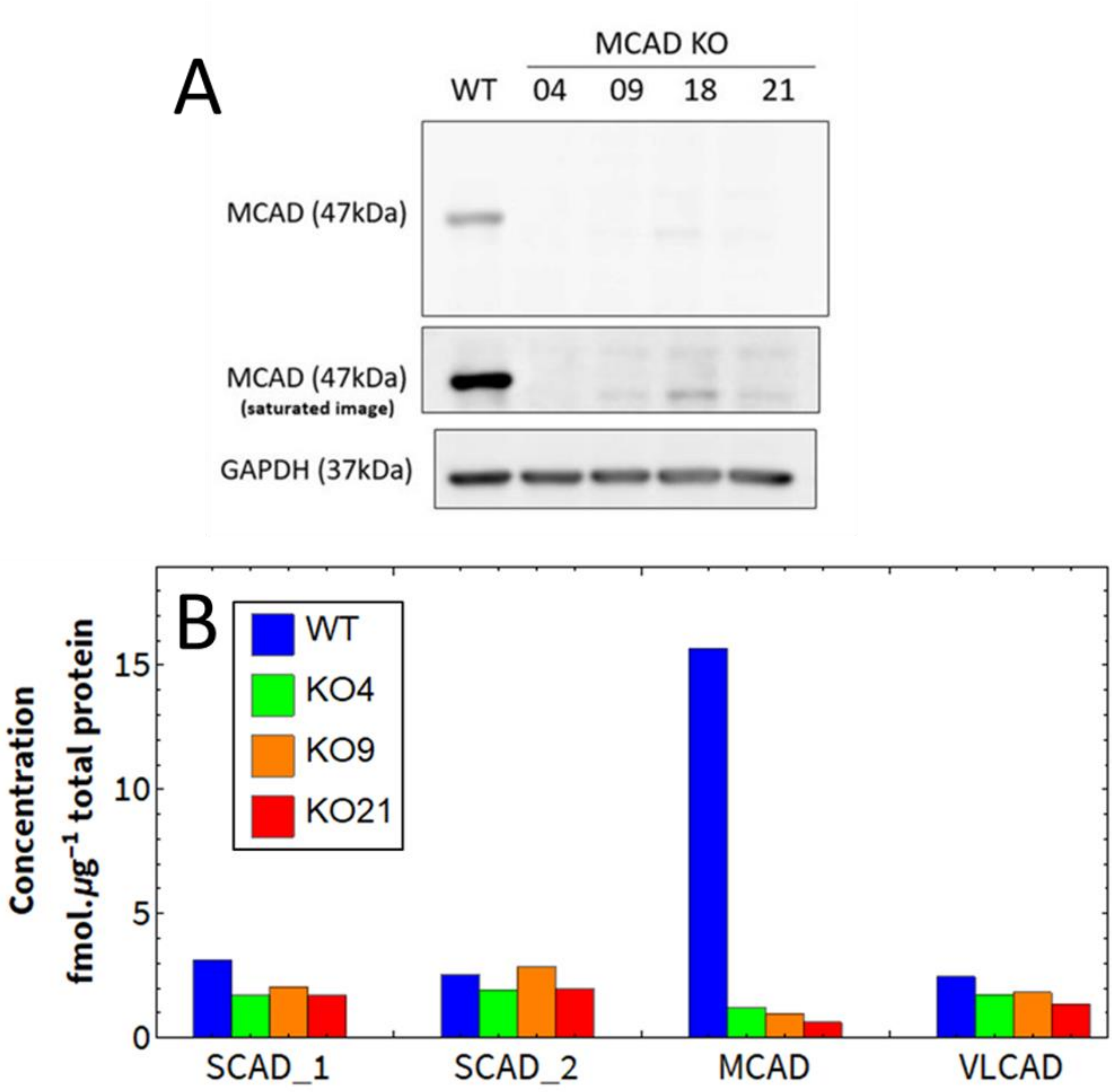

Figure S1. Confirmation of MCAD-knockout. A. Western blotting was performed on HepG2 cell lysates. GAPDH was the loading control. **B.** Targeted proteomics was performed on HepG2 cell lysates as described in the main text. Two SCAD peptides and one peptide each of MCAD and VLCAD are compared.
