# Supplementary material for: Personalised modelling of clinical heterogeneity between medium-chain acyl-CoA dehydrogenase patients
Source: BMC Biol. 2023 Sep 4;21:184. doi: 10.1186/s12915-023-01652-9 (PMC10478272; doi:10.1186/s12915-023-01652-9)
Supplement: Supplementary file 6 — Additional file 6: Figure S2. The simulated effect of MCADD on the acylation of CoA in the mitochondrion. Representation of the amount of the CoA pool that is free, or sequestered as acyl-CoAs of various chain lengths. [file 12915_2023_1652_MOESM6_ESM.pdf]

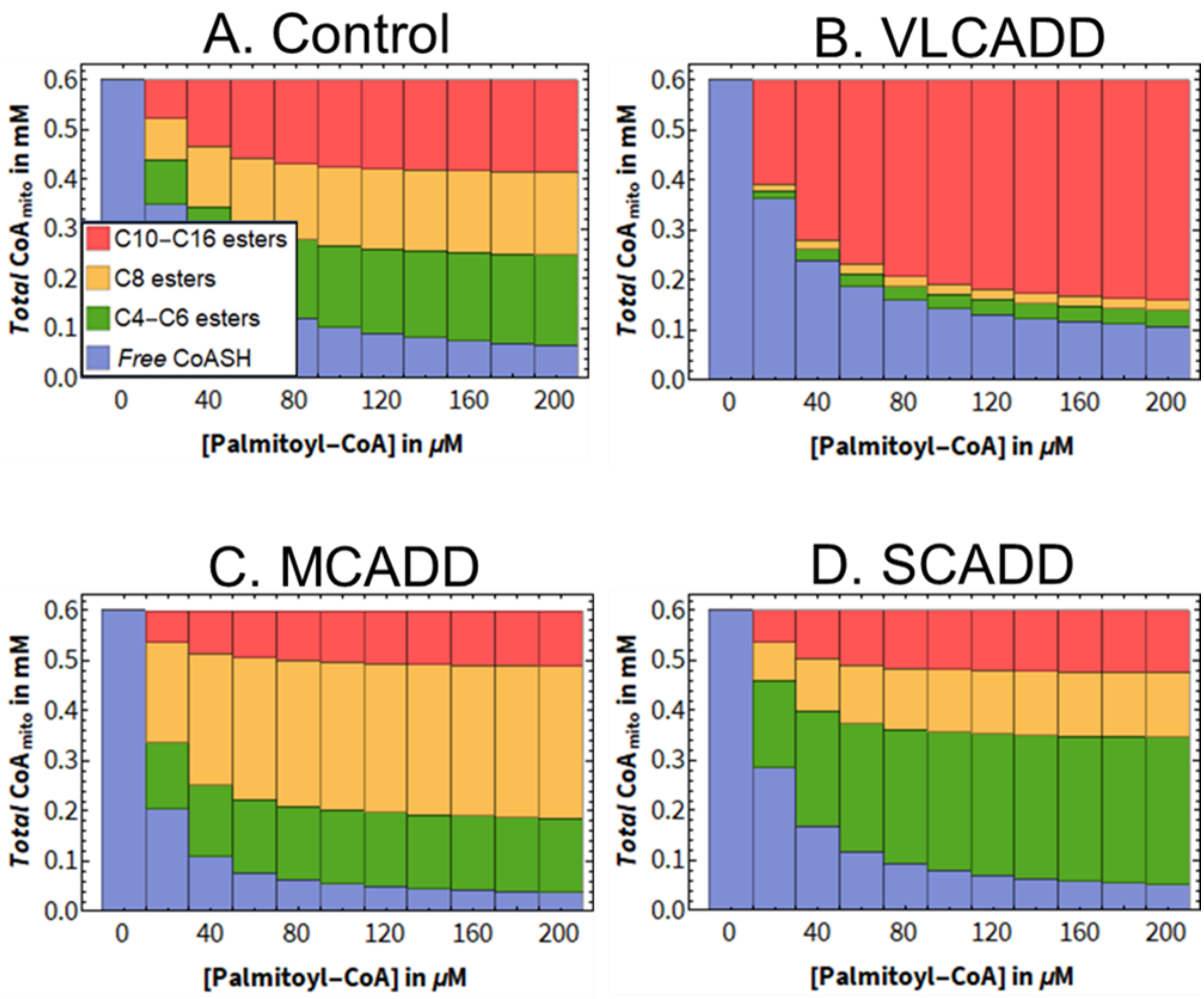

Figure S2. The simulated effect of MCADD on the acylation of CoA in the mitochondrion. In a computational model of human hepatic mFAO, total coenzyme A was simulated as the sum of mitochondrial CoASH (blue), short-chain CoA esters (C4, C6; green), C8 CoA esters (yellow), and long-chain CoA esters (C10, C12, C14, C16; red). The constant acetyl-CoA concentration was excluded from this figure, bringing the total concentration to 0.6 mM. **A.** Control model. **B.** VLCADD model (10% VLCAD activity). **C.** MCADD model (0% MCAD activity). **D.** SCADD model (0% SCAD activity).
