# Supplementary material for: Personalised modelling of clinical heterogeneity between medium-chain acyl-CoA dehydrogenase patients
Source: BMC Biol. 2023 Sep 4;21:184. doi: 10.1186/s12915-023-01652-9 (PMC10478272; doi:10.1186/s12915-023-01652-9)
Supplement: Supplementary file 7 — Additional file 7: Figure S3. The effect of different ACAD deficiencies in silico without metabolite partitioning. NADH production flux and mitochondrial CoASH concentration as a function of cytosolic palmitoyl-CoA concentration with metabolite partitioning removed. [file 12915_2023_1652_MOESM7_ESM.pdf]

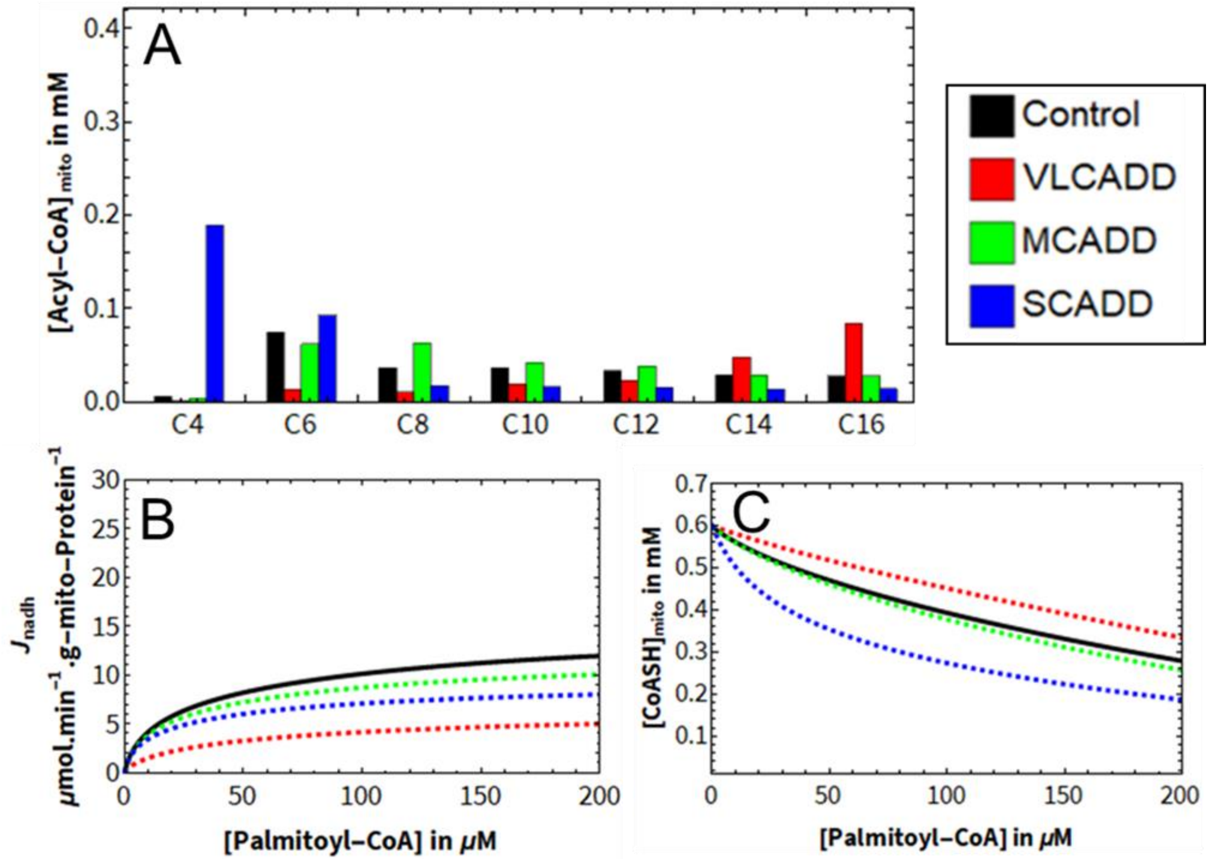

Figure S3. The effect of different ACAD deficiencies *in silico w*ithout metabolite partitioning. Using a computational model of human hepatic mFAO, a control and three different ACADD models were made. The residual activity reflects typical symptomatic patients: 0% for MCADD and SCADD, and 10% for VLCADD. Metabolite partitioning was included in these simulations. **A.** Mitochondrial acyl-CoA profile at 150 μM palmitoyl-CoA. **B.** NADH production flux. **C.** Steady-state mitochondrial CoASH.
