# Supplementary material for: Personalised modelling of clinical heterogeneity between medium-chain acyl-CoA dehydrogenase patients
Source: BMC Biol. 2023 Sep 4;21:184. doi: 10.1186/s12915-023-01652-9 (PMC10478272; doi:10.1186/s12915-023-01652-9)
Supplement: Supplementary file 8 — Additional file 8: Figure S4. The effect of different ACAD deficiencies in silico with fixed mitochondrial CoASH. NADH production flux and mitochondrial CoASH concentration as a function of cytosolic palmitoyl-CoA concentration with a constant CoASH of 600 μM. [file 12915_2023_1652_MOESM8_ESM.pdf]

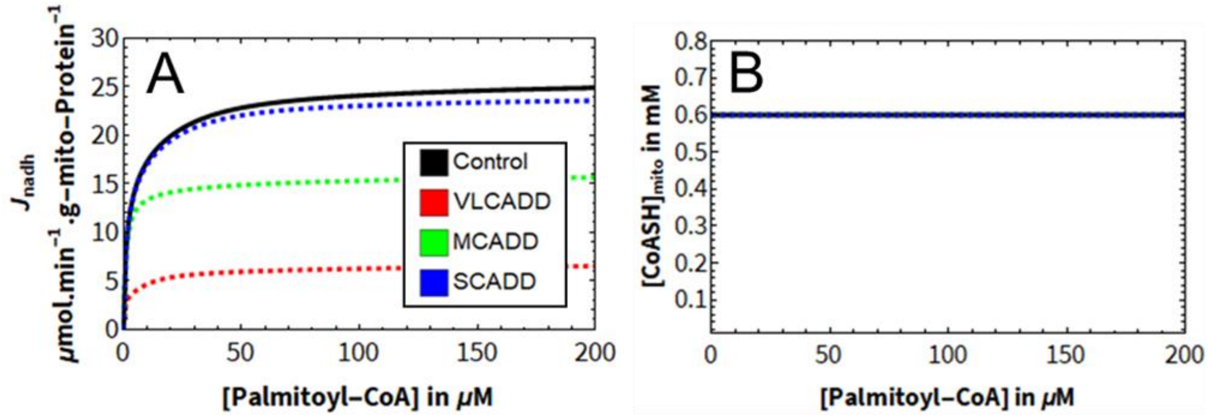

Figure S4. The effect of different ACAD deficiencies *in silico* with fixed mitochondrial CoASH. Using a computational model of human hepatic mFAO, a control and three different ACADD models were made. The residual activity reflects typical symptomatic patients: 0% for MCADD and SCADD, and 10% for VLCADD. All simulations were carried out at a constant mitochondrial CoASH concentration of 0.6 mM. **A.** NADH production flux. **B.** Steady-state mitochondrial CoASH.
