# Supplementary material for: Personalised modelling of clinical heterogeneity between medium-chain acyl-CoA dehydrogenase patients
Source: BMC Biol. 2023 Sep 4;21:184. doi: 10.1186/s12915-023-01652-9 (PMC10478272; doi:10.1186/s12915-023-01652-9)
Supplement: Supplementary file 9 — Additional file 9: Figure S5. Control analysis at low substrate, high malonyl-CoA concentrations, control MCAD activity. Metabolic control analysis showing a case where CPT1 has the highest flux control. [file 12915_2023_1652_MOESM9_ESM.pdf]

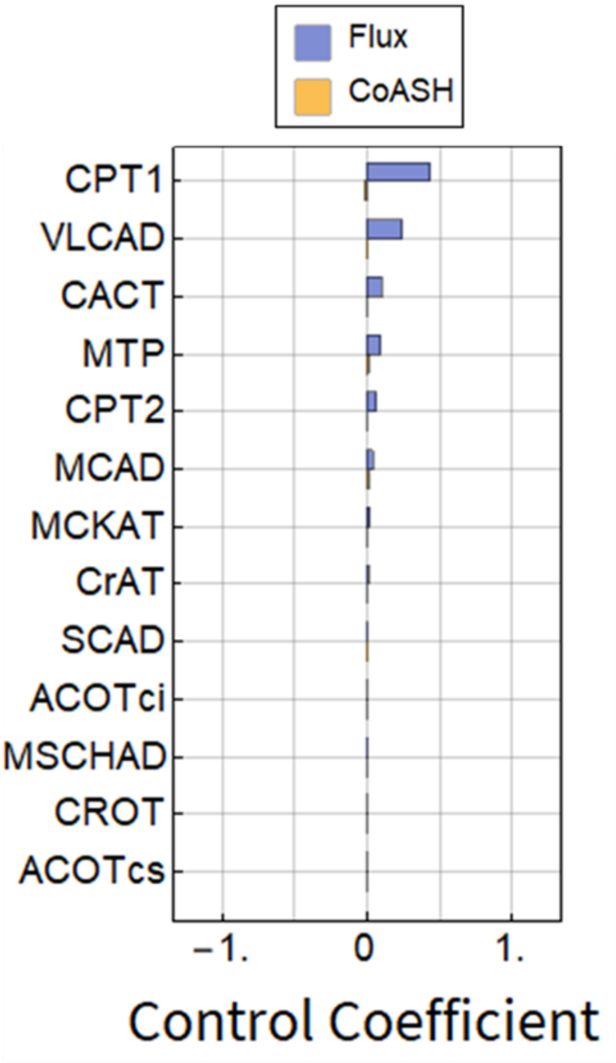


**Figure S5. Control analysis at low substrate, high malonyl-CoA concentrations, control MCAD activity.** Flux and mitochondrial CoASH concentration control coefficients (blue and yellow, respectively) in a control model. Enzymes are displayed in descending order according to absolute flux control. Model simulations were carried out at 2 µM cytosolic palmitoyl-CoA and a mitochondrial acetyl-CoA concentration of 120 µM (lowest value retrieved from literature). High malonyl-CoA (100 µM in the cytosol) and zero sequestration of total mitochondrial CoA by intermediates from other pathway are also assumed.
