# Supplementary material for: Personalised modelling of clinical heterogeneity between medium-chain acyl-CoA dehydrogenase patients
Source: BMC Biol. 2023 Sep 4;21:184. doi: 10.1186/s12915-023-01652-9 (PMC10478272; doi:10.1186/s12915-023-01652-9)
Supplement: Supplementary file 10 — Additional file 10: Figure S6. Control analysis at low acetyl-CoA. Metabolic control analysis showing high VLCAD flux control but negative CoASH concentration control. [file 12915_2023_1652_MOESM10_ESM.pdf]

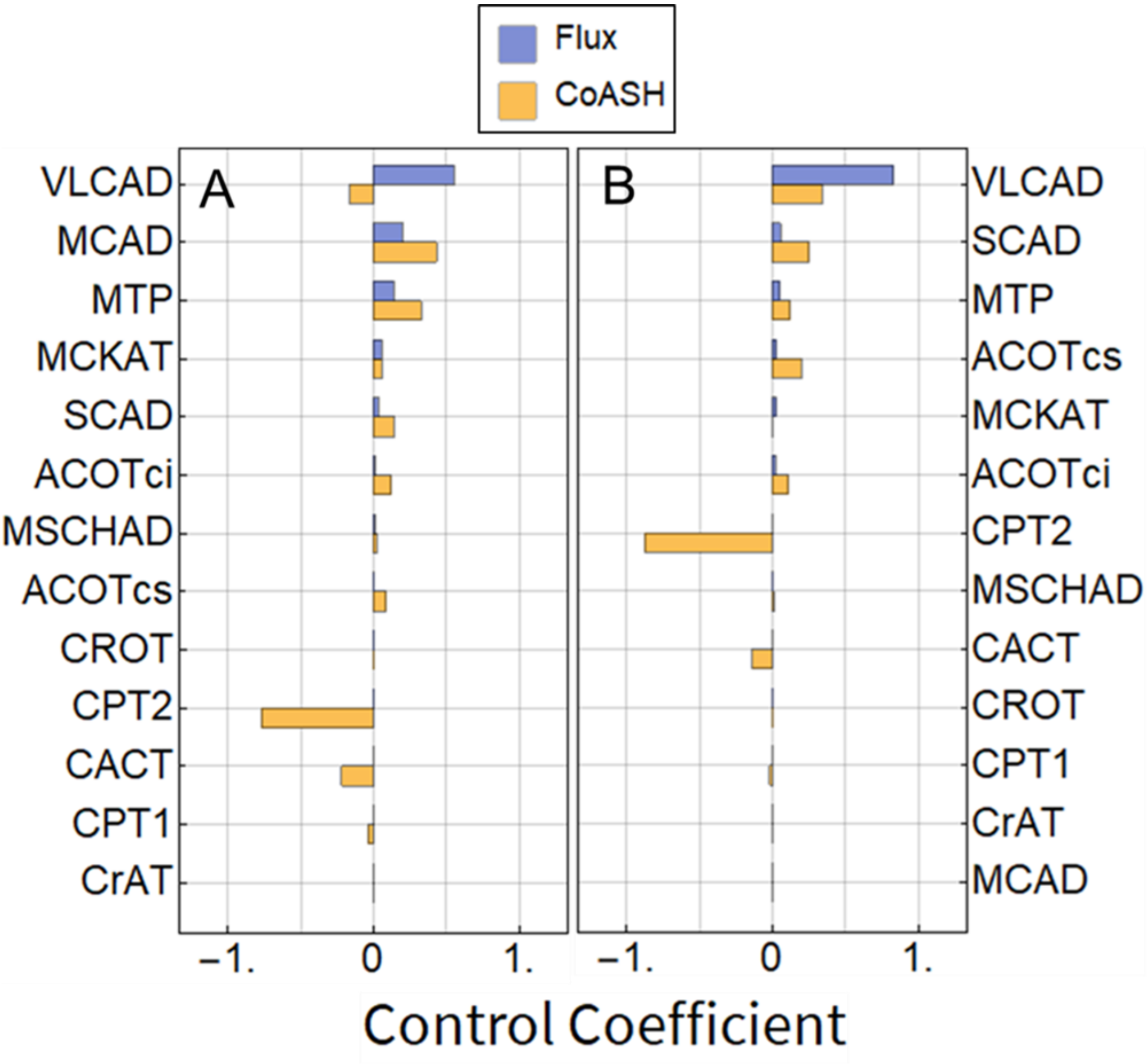

Figure S6. Control analysis at low acetyl-CoA. Flux and mitochondrial CoASH concentration control coefficients (blue and yellow, respectively) in a control (**A**) and MCADD (**B**) model. Enzymes are displayed in descending order according to absolute flux control. Model simulations were carried out at 150 µM cytosolic palmitoyl-CoA and a mitochondrial acetyl-CoA concentration of 120 µM (lowest value retrieved from literature).
