# Supplementary material for: Personalised modelling of clinical heterogeneity between medium-chain acyl-CoA dehydrogenase patients
Source: BMC Biol. 2023 Sep 4;21:184. doi: 10.1186/s12915-023-01652-9 (PMC10478272; doi:10.1186/s12915-023-01652-9)
Supplement: Supplementary file 12 — Additional file 12: Figure S7. Effect of possible rescues of mitochondrial CoASH and steady-state mFAO flux in a control model. Effect of incremental changes in VLCAD, SCAD, MTP, ACOT, and CPT2 in a control model activity on NADH flux and CoASH concentration. [file 12915_2023_1652_MOESM12_ESM.pdf]

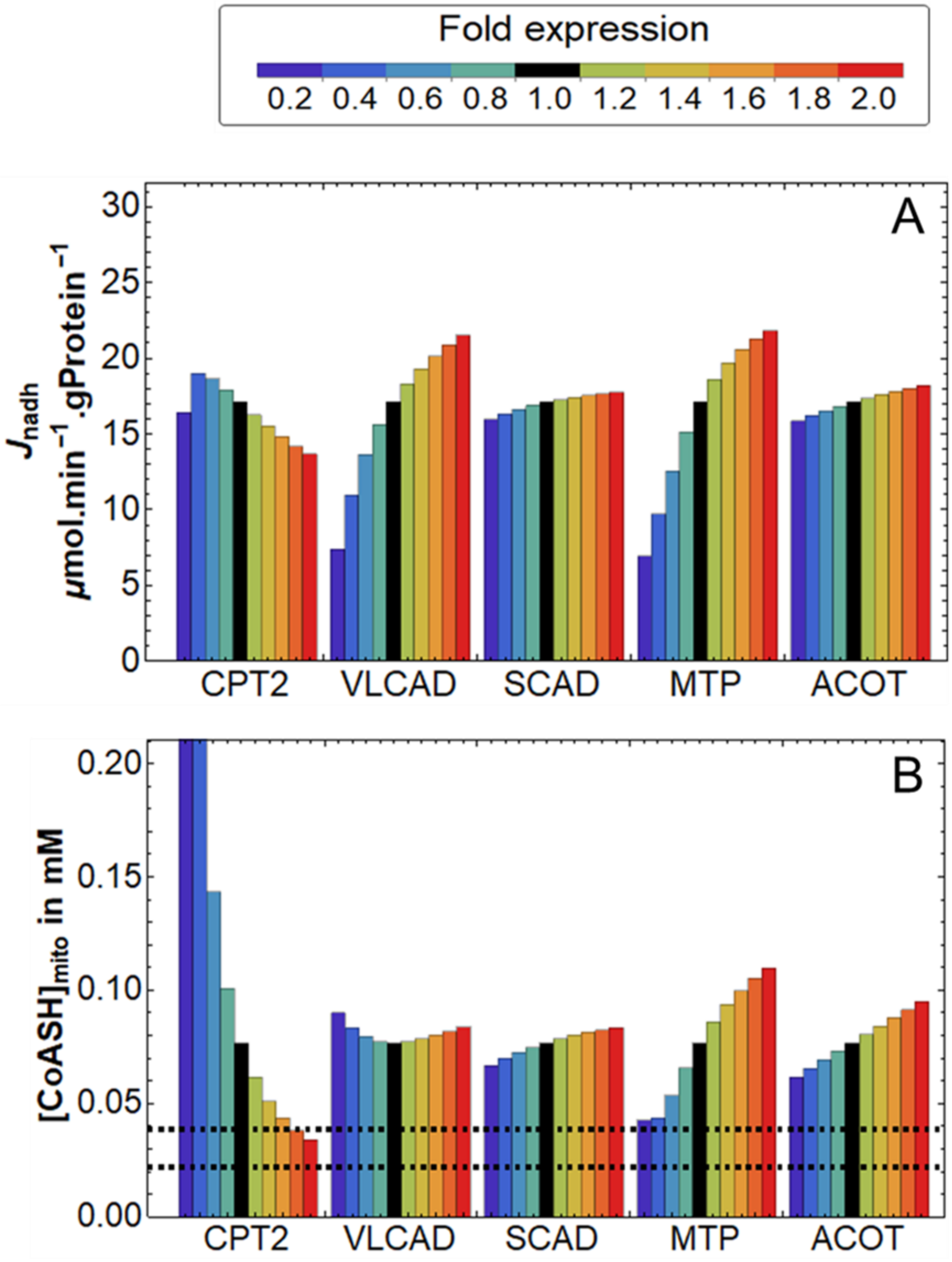

Figure S7. Effect of possible rescues of mitochondrial CoASH and steady-state mFAO flux in a control model. Using a control computational model of human hepatic mFAO, different expression levels of the enzymes identified as possible rescues by metabolic control analysis were incrementally varied. The reference value (1-fold expression) is given in black. All simulations were carried out at 150 μM cytosolic palmitoyl-CoA. The dashed lines in **B** indicate the K_m values of various mitochondrial enzymes that require CoASH as substrate (**Table 2**). CPT2, VLCAD, SCAD, MTP, and ACOT were varied between 20 and 200% of basal expression levels. ACOT was varied by simultaneously increasing the expression of both ACOTs in the model. **A.** NADH production flux. **B.** Steady-state mitochondrial CoASH.
