# Supplementary material for: Personalised modelling of clinical heterogeneity between medium-chain acyl-CoA dehydrogenase patients
Source: BMC Biol. 2023 Sep 4;21:184. doi: 10.1186/s12915-023-01652-9 (PMC10478272; doi:10.1186/s12915-023-01652-9)
Supplement: Supplementary file 13 — Additional file 13: Figure S8. Possible rescues of steady-state mFAO flux in an MCADD model with fixed mitochondrial CoASH. Effect of incremental changes in VLCAD, SCAD, MTP, ACOT, and CPT2 activity with a constant CoASH of 600 μM on NADH flux in a control and MCADD model. [file 12915_2023_1652_MOESM13_ESM.pdf]

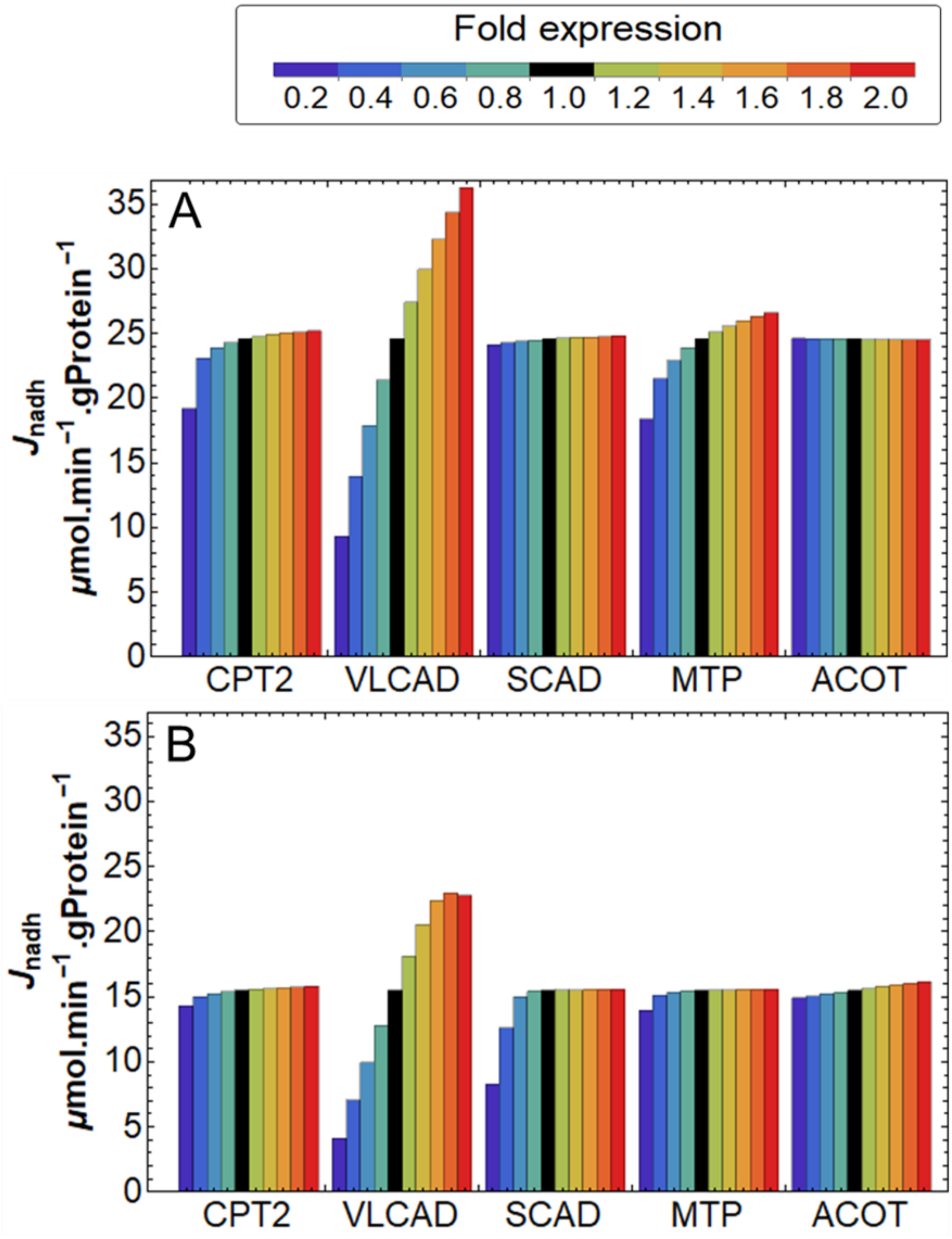

Figure S8. Possible rescues of steady-state mFAO flux in an MCADD model with fixed mitochondrial CoASH. An MCADD computational mFAO model was simulated at different expression levels of the enzymes identified as possible rescues by metabolic control analysis. Mitochondrial CoASH was fixed at 0.6 mM. The reference value (1-fold expression) is given in black. All simulations were performed at 150 µM cytosolic palmitoyl-CoA. CPT2, VLCAD, SCAD, MTP, and ACOT were varied between 20 and 200% of basal expression levels. ACOT was varied by simultaneously varying both ACOTs in the model. Both figures represent the NADH production flux. **A.** Control model. **B.** MCADD model.
