# Supplementary material for: Personalised modelling of clinical heterogeneity between medium-chain acyl-CoA dehydrogenase patients
Source: BMC Biol. 2023 Sep 4;21:184. doi: 10.1186/s12915-023-01652-9 (PMC10478272; doi:10.1186/s12915-023-01652-9)
Supplement: Supplementary file 14 — Additional file 14: Figure S9. Protein concentration per technical replicate. Concentration of mFAO proteins in MCADD and control fibroblasts. [file 12915_2023_1652_MOESM14_ESM.pdf]

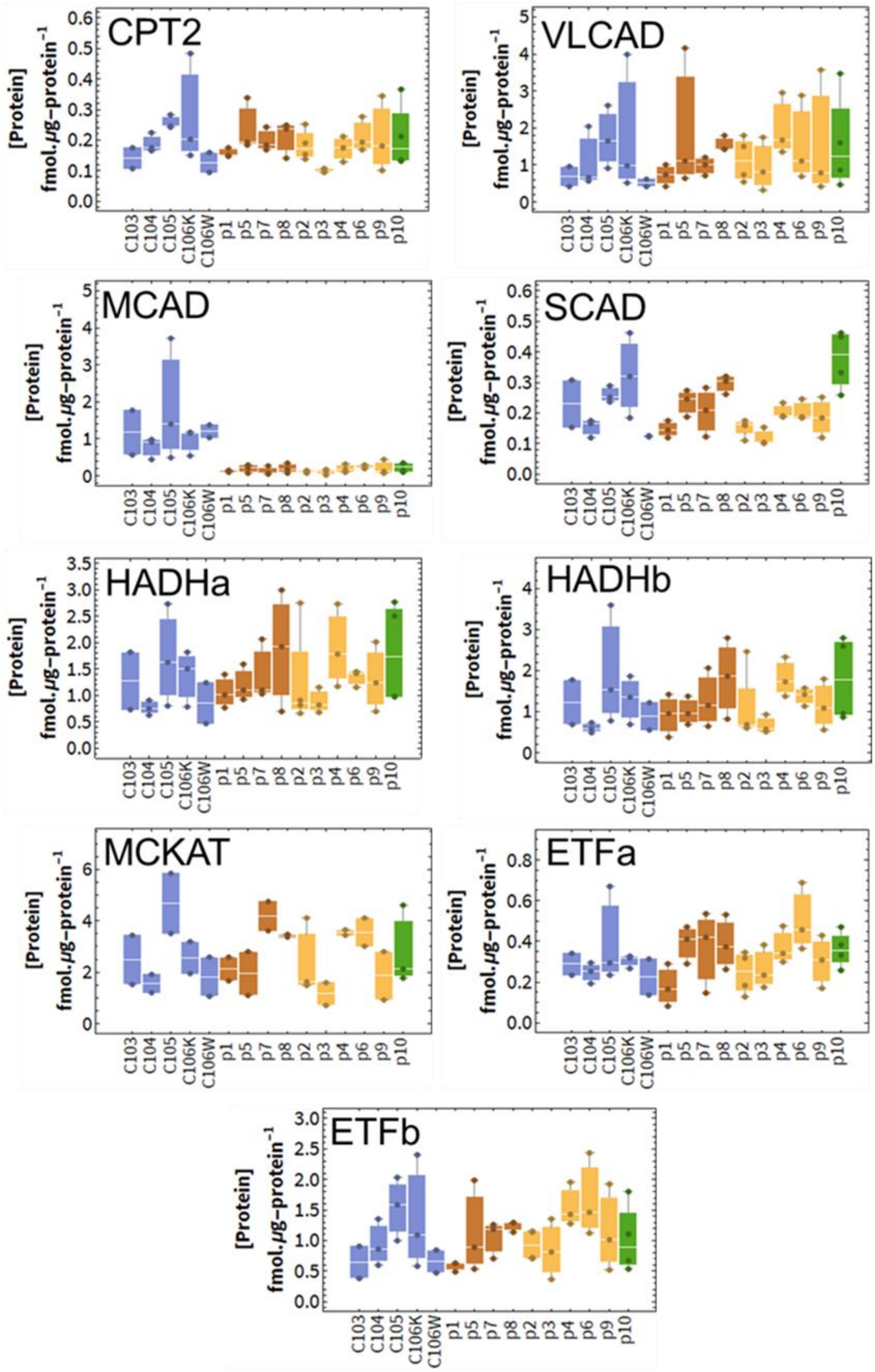

Figure S9. Protein concentration per technical replicate. The bars indicate the range of measurements per subject in the retrospective cohort. Each point within represents one fibroblast culture from a given patient (p2 and p10 are n=4, all others are n=3,). Colour codes indicate the

phenotypic classification of each individual: *control* (blue), *symptomatic* (brown), *early detection* (yellow), or *asymptomatic* (green). Case descriptions in **Additional File 15: Table S4**.
