# Supplementary material for: Personalised modelling of clinical heterogeneity between medium-chain acyl-CoA dehydrogenase patients
Source: BMC Biol. 2023 Sep 4;21:184. doi: 10.1186/s12915-023-01652-9 (PMC10478272; doi:10.1186/s12915-023-01652-9)
Supplement: Supplementary file 16 — Additional file 16: Figure 10. Western blots for confirming MCAD-KO. Unedited, uncropped Western blots used to confirm the absence of MCAD in the MCAD-KO cell lines. [file 12915_2023_1652_MOESM16_ESM.pdf]

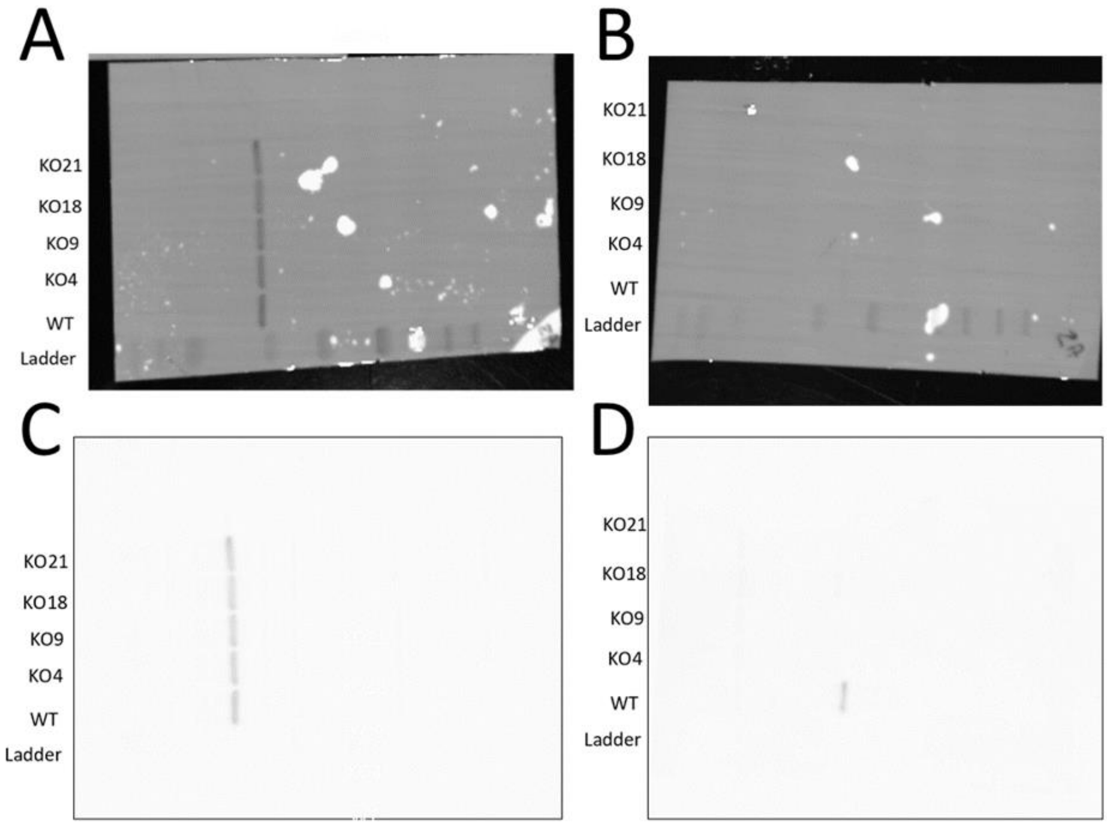

Figure S10. Western blots for confirming MCAD-KO. Original, uncropped Western blots used to confirm the absence of MCAD in the MCAD-KO cell lines. **A.** GAPDH and ladder. **B.** MCAD and ladder. **C.** GAPDH and ladder, saturated image. **D.** MCAD and ladder, saturated image.
